# Competition-driven selection in covalent dynamic networks and implementation in organic reactional selectivity†
†Electronic supplementary information (ESI) available: Experimental details, NMR spectra, synthetic protocols and mathematical model for formal dynamic combinatorial library description. See DOI: 10.1039/c5sc04924e


**DOI:** 10.1039/c5sc04924e

**Published:** 2016-02-10

**Authors:** P. Kovaříček, A. C. Meister, K. Flídrová, R. Cabot, K. Kovaříčková, J.-M. Lehn

**Affiliations:** a Institut de Science et d'Ingénierie Supramoléculaires , Université de Strasbourg , 8 allée Gaspard Monge , 67000 Strasbourg , France . Email: lehn@unistra.fr

## Abstract

Competition among reagents in dynamic combinatorial libraries of increased complexity leads to reactional self-sorting (improved regioselectivity) in mixtures of aldehydes and oligoamines. High selectivity of a given library component is transferred to a different reacting component of low selectivity through a network of underlying equilibrating reactions which provide component exchange between all species. The selectivity of various carbonyl compounds in reactions with amines was also assessed towards the formation of defined sequences of residues along oligoamine chains. The approach was further exploited for defining selective dynamic protecting groups (DPGs), based on the reversible linkage between the substrate and the protecting group. They represent an intermediate approach between the conventional protecting groups and the protecting-group-free approach in organic synthesis. Removal of the protecting group is effected *via* dynamic exchange trapping by formation of a more stable product. The establishment of equilibrium eliminates the need for isolation and purification of the dynamically protected intermediate(s) and enables as well the selective sequential derivatisation of oligoamines. The DPG concept can be generalised to other reversible reactions and can thus represent a valuable alternative in the design of total synthesis of complex molecules.

## Abstract 2

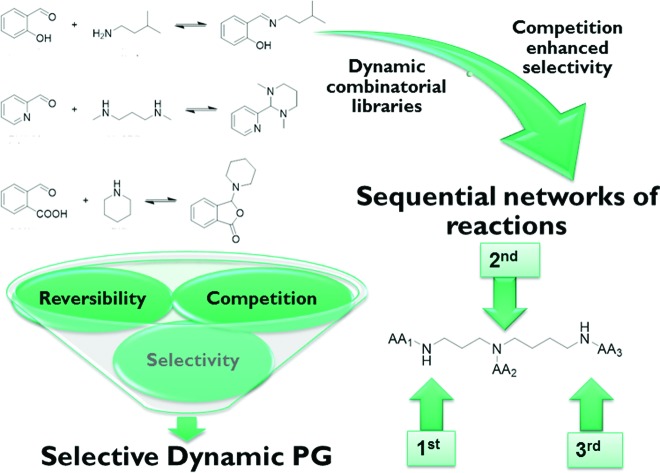
Competition among reagents in dynamic combinatorial libraries of increased complexity leads to reactional self-sorting. This fundamental principle allowed development of selective dynamic protecting groups for controlled sequential derivatization of polyamines.

## Introduction

Dynamic covalent libraries (DCLs)^1^–^11^ are formed by reversible combinatorial linkage of molecular components generating an equilibrium mixture of constituents under thermodynamic control. The reversibility of the linkage allows for constitutional exchange giving access to all possible combinations of components. By application of a stimulus, the DCL is able to undergo constitutional adaptation whereby some species are amplified at the expense of others that are depleted. These features make dynamic covalent chemistry (DCC) attractive for application in material science,^12^–^20^ surface modification,^21^ synthesis of nanoarchitectures,^22^–^25^ design of receptors^7^ and sensors^26^ as well as in the search for biologically active compounds.^2^,^27^–^32^ In fact, only “virtual presence”^1^ of, *i.e.* access to, all combinations is required as the amplified species can be formed upon the application of the stimulus – in the terms of the “lock and key” principle, one may say that the lock assembles its key.

Imines, resulting from the reversible condensation of a carbonyl component with an amine, are of particular interest for setting up DCLs, due to the ease of formation and exchange, usually under mild conditions. The heteroatomic imine bond is also satisfactorily orthogonal^33^–^36^ to many other reversible bonds and its dynamic nature can be “frozen” by reduction or other transformations.^37^ Simplification of the complex mixture of species in the library is achieved through adaptive sorting,^38^–^41^ for example in course of crystallisation,^42^–^44^ oxidation,^45^ distillation^46^,^47^ or coordination.^48^–^52^

Herewith we report the operation of a *reactional self-sorting* process in DCLs of imines of increasing complexity, driven by competition among the reagents in mixtures of aldehydes and oligoamines components and leading to improved selectivity in product formation and/or in site of reaction. The aldehydes in the studied DCLs are moderately selective in reactions with different amines to form various products such as imines, aminals or amino-lactones in the case of *o*-carboxybenzaldehyde.^53^,^54^ Competition between an increasing number of aldehydes for a given mixture of amines leads to increased selectivity of each of them. It leads to a simplification of the final composition resulting from the complexity of the DCL and its collapse into a system of reduced multiplicity (and lower entropy), amounting to a state that may be termed “simplexity” in a process of general type from chemical to biological systems.^55^,^56^ In fact, any recognition process, be it reactional or interactional (see Fig. 3 in ref. 57) results in the reduction of the complexity of a mixture, of its simplification, through the operation of competitive selection. On the supramolecular/interactional level it is expressed in the “instructed mixture paradigm”, whereby the behaviour of mixtures is driven by the instructions (molecular information) present in its members resulting in self-recognition (or self-sorting), a self-process,^58^ belonging to the general phenomenon of self-organization.^9^,^59^ The term “simplexity” does not carry the meaning that the system in itself is less complex, as there is in fact an increased complexity (increased number of reacting species) underlying and resulting in the simplification (increased selectivity) observed. We also propose a formal approach for the representation and comparison of DCLs (see below and ESI†) which has been used for evaluating the efficiency of selection in the self-sorting processes investigated here within the realm of dynamic covalent chemistry (DCC).^1^–^5^,^28^–^30^,^57^

## Aldehyde–amine dynamic covalent libraries

In view of the ubiquity of amines and imines in organic chemistry and in biochemistry, as well as the ability to control each partner in the reaction, condensations between aldehydes and amines were taken as model reactions in order to probe reaction selectivity in competitive dynamic reversible systems.

**Fig. 1:**
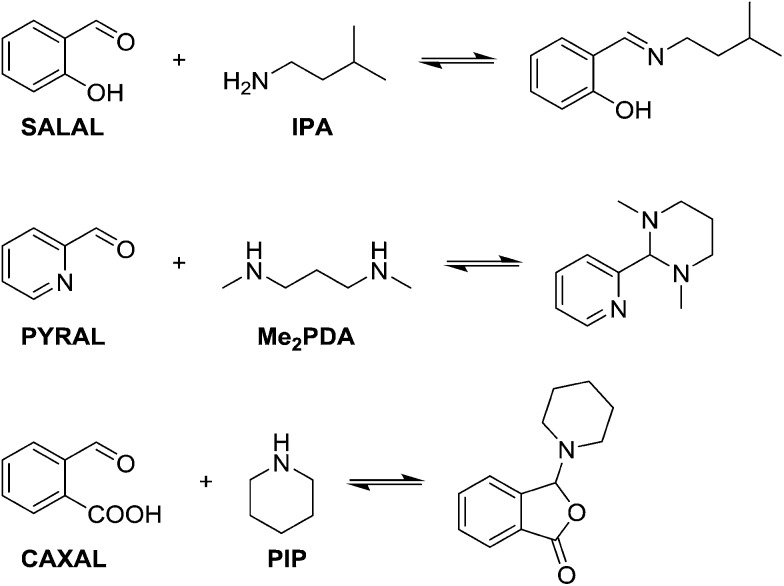
Schemes of anticipated reaction outcome of different aldehydes and amines with their acronyms used in the study. **SALAL** forms preferentially imines and **PYRAL** cyclic aminals. **CAXAL** is capable of reacting at a secondary amine site with formation of a lactone ring.

At first, different aldehydes were reacted with different amines in order to test their differences in reactivity and the nature of the product formed. Salicylaldehyde (**SALAL**), pyridine-2-carboxaldehyde (**PYRAL**) and 2-carboxy-benzaldehyde (**CAXAL**) were selected as prototypical aldehydes. On the other hand, isopentylamine (**IPA**), *N*,*N*′-dimethyl-1,3-diaminopropane (**Me_2_PDA**) and piperidine (**PIP**) were selected as their amine counterparts. As solvent a mixture of *d*_6_-DMSO with 1% of D_2_O was used to guarantee solubility of all partners, in which the water addition maintains constant water content during the aldehyde–amine condensation. On the basis of previous studies, differences in reaction outcome was expected for the various aldehyde–amine pairs,^53^ anticipating that: (a) **SALAL** would have a preference for primary amines to form imines,^53^ (b) **PYRAL** would react with diamines to provide a cyclic aminal^53^ and (c) **CAXAL** would react with secondary amines to afford amino-lactones through trapping of the intermediary iminium by the neighbouring carboxylate group.^54^ These anticipated selectivities towards formation of different condensation products were verified in separate experiments: **SALAL** reacted with **IPA** to provide the expected imine, **PYRAL** gave the six-membered cyclic aminal with **Me_2_PDA**, and **CAXAL** indeed reacted with **PIP** to form the amino-lactone. These reactions proceeded with quantitative conversion and the resulting preferentially formed products may be considered as “matching pairs”, effecting reactional recognition, like supramolecular systems effect interactional recognition (Fig. 1).^4^,^57^

### [2 × 2] aldehyde–amine mixtures

#### Selection by preferential imine/lactone formation

In order to explore selection in a [2 × 2] competition, the case of a mixture of the two aldehydes **SALAL–CAXAL** and the two amines **IPA–PIP** was examined in detail.

Checking first the combinations opposite to the “matching pairs” (see above) showed that both aldehydes reacted with both amines: when **CAXAL** was mixed with 1 eq. of **IPA**, equilibrium was reached almost instantaneously giving a dynamic mixture of the imine and lactone formed with **IPA** (details in ESI, Section 3.2†); on the other hand, **SALAL** reacted with **PIP** in approximately 30% conversion to the aminal formed from two amine molecules and one aldehyde. In subsequent experiments, the propensity towards the formation of the “matching pair” product was tested in presence of a competing amine, *i.e.* each aldehyde was reacted with an equimolar mixture of the two amines. **SALAL** showed a very high preference for imine formation reacting solely with **IPA** and leaving **PIP** unreacted in the solution. However, the conversion of the aldehyde in this case was not complete, approximately 10% remaining unreacted. In contrast, **CAXAL** under the same reaction conditions provided two products: the lactone on the **PIP** ring was formed in about 39% yield and the dynamic imine–lactone product from **IPA** was present in about 61% yield (details in the ESI, Section 3.2†). Finally, the full [2 × 2] library was considered: **SALAL** and **CAXAL** were mixed with both **IPA** and **PIP** in equimolar amounts and the resulting product mixture was examined using NMR spectroscopy. Equilibrium was reached after 5 hours and the mixture contained only the two “matching pair” species, the imine **SALAL–IPA** and the lactone **CAXAL–PIP**, both in quantitative conversion.

**Fig. 2:**
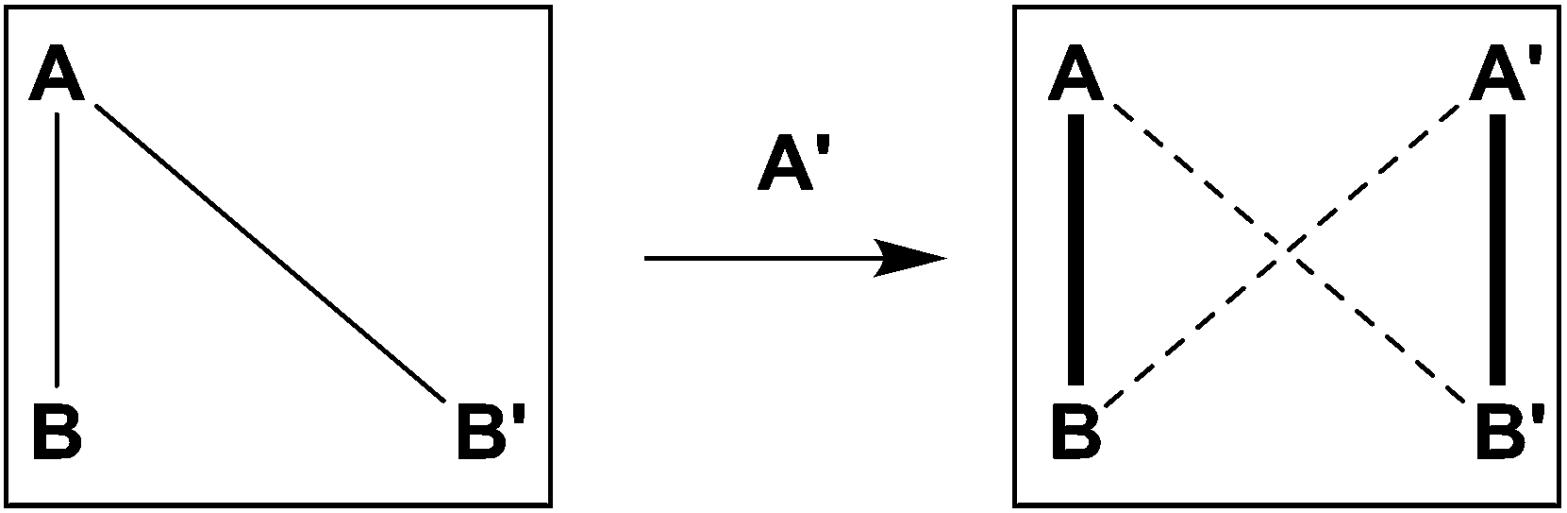
Schematic representation of the process of competitive amplification (enhanced selection) induced by increasing the complexity of the mixture: while **A** is capable of reacting with both **B** and **B′**, addition of **A′** leads to enforced formation of the **A′B′** product which traps the **B′** component and therefore depletes the **AB′** constituent. Due to the underlying network of equilibrating reactions this also leads to amplified formation of the **AB** constituent in a type of agonist amplification process of **AB** by the formation of **A′B′**. The amplified agonistic species are represented by thick lines on the vertices of the square while the depleted species are denoted by the striped diagonals.

This remarkable simplicity achieved in the comparatively complex mixture, contrasts with the ability of both aldehydes to react with both amines, and *vice versa* each amine with the two aldehydes. It demonstrates the occurrence of a selectivity enhancement of a poor selector by competition with a strong selector. Specifically, a species reacting non-selectively with several compounds is brought to become selective towards a given product, when put in presence of a competing species of high selectivity which monopolises one of the components. The process amounts to a *competitive selectivity enhancement*. The formation of the matching pair products can also be seen as resulting from agonist amplification^9^,^57^ by competition *via* component exchange through an underlying network of equilibrating reactions (Fig. 2).

Stoichiometry is of course a crucial parameter providing the enhanced selection of the inherent self-sorting process. In the present case, the components react in 1 : 1 ratio to form the preferred product. Therefore, the initial mixture must contain the components in this ratio so that the condensation of the matching pair in quantitative conversion depletes all of the starting materials, which in turn do not interfere with the formation of the second matching pair in the complex system. This is clearly demonstrated by the fact that whereas the aforementioned mixture **SALAL–CAXAL–IPA–PIP** can give, in principle, 14 products (accounting for 2 imines, 4 homo- and 2 heteroaminals, 4 hemiaminals and 2 lactones), only two products are observed in the mixture containing all four species in equimolar amounts.

#### Selection by aminal formation

**PYRAL** offers the opportunity of considering selection by the formation of aminal species, different from imine and lactone. Indeed, **PYRAL** efficiently forms both five- and six-membered-ring aminals with *N*,*N*′-dimethyl-1,2-diaminoethane (**Me_2_EDA**) or *N*,*N*′-dimethyl-1,3-diaminopropane (**Me_2_PDA**), reaching quantitative conversion when reacted separately with either of the two diamines. On the other hand, when it was reacted with an equimolar mixture of **Me_2_EDA** and **Me_2_PDA**, the five-membered ring was formed preferentially (77% conversion to the aminal of **Me_2_EDA**). Both **SALAL** and **CAXAL** reacted as well with these diamines giving the corresponding aminals in high conversion (about 90% for **SALAL** and quantitative for **CAXAL**). Libraries similar to the previous one were examined using **PYRAL** and its matching diamine (**Me_2_EDA** and **Me_2_PDA**) combined either with the **SALAL–IPA** or the **CAXAL–PIP** pair. The experiments (described in detail in the ESI, Section 3.2 and 3.3†) revealed that although all aldehyde–amine combinations were forming efficiently, high selectivity for the matching pairs was observed when all constituents of the library were mixed in equimolar ratio. In particularly, for the **SALAL–PYRAL–IPA–Me_2_PDA** mixture the **SALAL–IPA** imine and the **PYRAL–Me_2_PDA** aminal were formed in 80% conversion (and the non-matching pairs **SALAL–Me_2_PDA** and **PYRAL–IPA** in 20%), while in the case of the **PYRAL–CAXAL–Me_2_EDA–PIP** mixture the **PYRAL–Me_2_EDA** aminal and the **CAXAL–PIP** lactone were present in 97% conversion (and the non-matching pairs were formed only in 3%).

#### Selection in extended aldehyde–amine dynamic combinatorial libraries

**Fig. 3:**
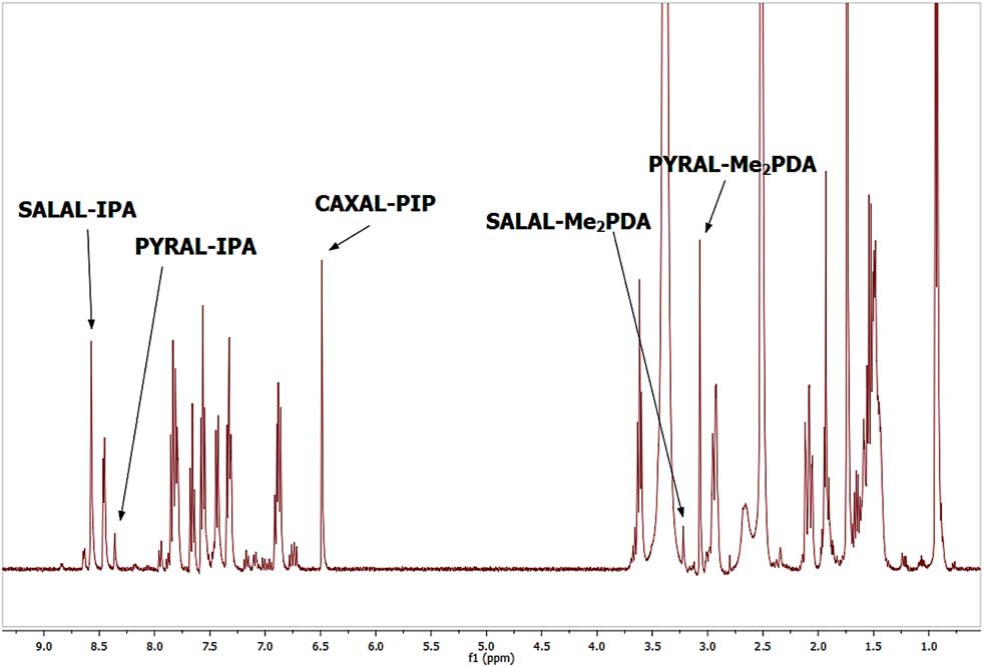
^1^H-NMR spectrum of the [3 × 3] aldehyde–amine library with assignment of the peaks. Three major species are formed: the imine of **SALAL** and **IPA**, the aminal of **PYRAL** and **Me_2_PDA**, and the lactone of **CAXAL** and **PIP**, the latter being the only product of this aldehyde.

The three [2 × 2] libraries described above form, when combined, a [3 × 3] dynamic library composed of three aldehydes and three amine partners. In this vein, a mixture of **SALAL**, **PYRAL** and **CAXAL** was combined with a mixture of **IPA**, **Me_2_PDA** and **PIP** (all equimolar) and the NMR spectra were recorded (Fig. 3). Equilibration of the mixtures was accelerated by heating at 60 °C overnight. The equilibrated samples contained the imine of **SALAL** and **IPA** in 88% conversion and the aminal of **SALAL** and **Me_2_PDA** in 12%, whereas the imine to aminal ratio of **PYRAL** was exactly the opposite, and **CAXAL** provided quantitatively the lactone with **PIP**. The system thus displayed selectivity in product formation in each case as well as a remarkable selectivity inversion when comparing the two cases, which display imine/aminal ratios of 88/12 = 7.

As the final concentration of species can play a role in the selection process,^60^ we repeated the library experiments at several concentrations: 4, 10, 20, 60 and 100 mM of the components. The equilibrium composition of the library in this range was constant showing no concentration dependency.

The library was also assessed for the effect of pH. To this end, the pre-equilibrated library was examined on addition up to 4 equivalents of acid (MeSO_3_H) or up to 2 eq. of base (*t*-BuOK). Addition of 1 eq. of acid leads to almost complete hydrolysis of the aminals of both **SALAL** and **PYRAL**, while on addition of the second equivalent of MeSO_3_H the imine hydrolysis reached 60%. Continued titration by acid led to complete hydrolysis of the imines and extensive hydrolysis of the **CAXAL** derived lactone, finally giving only the hydrolysed products, after addition of 4 equivalents of acid. In contrast, upon titration by base, disappearance of the lactone signals was observed. Interestingly, this lactone depletion was not accompanied by the emergence of the aldehyde peak of **CAXAL**, but rather of a new imine of **CAXAL** with **IPA** and liberation of **SALAL** was observed. This example clearly displays the operation of the underlying network of interconnected equilibrating reactions which leads to the complex adaptation of the dynamic library to a stimulus.

The selectivity enhancement displayed here for [*n* × *n*] dynamic libraries represents a process of competitive selection, whereby simplification results from competition within the set of equilibrating constituents undergoing component exchange. This behaviour relates also to the process of co-evolution in a dynamic library, leading to the synergistic expression of given constituents.^9^,^61^

We have developed a formal method for quantification of selection in dynamic combinatorial libraries and especially for (self)-sorting processes which involve coupled reactions. It is based on a matrix representation of the combinatorial library including the particular case of the selection induced by increased complexity of the mixture (see ESI, Section 2,† for detailed description).

### Kinetics in dynamic reaction selectivity

The key aspect of the field of dynamic covalent chemistry (DCC) is that it is governed by thermodynamics and therefore relates to the equilibrium composition. However, the equilibration of the mixture proceeds *via* component exchanges, each with a given rate constant, and the equilibrium is thus established when the sum of all reaction rates is zero. Kinetics are therefore intimately involved in the status of the composition of a dynamic set at a given time.^62^ It is in particular the case when considering the vastly different rates of C=N double bond formation between different carbonyl groups and different types of amino compounds.^63^ Such kinetic features can be briefly illustrated on the library consisting of **SALAL**, **CAXAL**, **IPA** and **PIP**.

**Chart 1:**
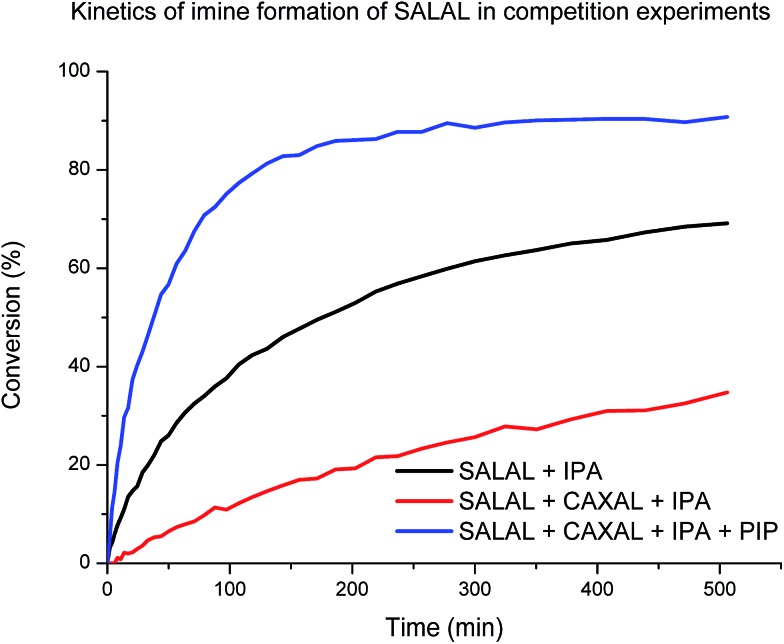
Relative percentage of the imine formed by the reaction of **SALAL** + **IPA** plotted as a function of time. The rate is significantly lower when **CAXAL** is added to the mixture as a result of competition of two aldehydes for one amine. Interestingly, when **PIP** is also added, the rate is higher than the previous two rates, indicating catalysis due to **PIP**. Kinetic experiments were performed in solution 20 mM for each compound in *d*_6_-DMSO + 1% D_2_O with 0.8 M triethanolamine buffer.

When **SALAL** was reacted with **IPA** (20 mM, buffered *d*_6_-DMSO, see ESI, Section 3,† for details) the formation of the imine followed second order kinetics with the rate constant of 0.01 M^–1^ s^–1^ and 90% overall conversion to the imine (note that in the reaction without a buffer the conversion was quantitative). When the experiment was repeated in presence of 1 eq. of **CAXAL**, significantly slower rate (*k* = 0.001 M^–1^ s^–1^) and only 77% conversion of **SALAL** was observed (Chart 1), showing that competition of the two aldehydes for one amine affects both the equilibrium (conversion) and the kinetics (rate of formation). Finally, when the experiment was performed in presence of 1 eq. of both **CAXAL** and **PIP**, forming the corresponding lactone, the original **SALAL** conversion of 90% was restored and the rate of formation was even accelerated (*k* = 0.03 M^–1^ s^–1^) due probably to the catalytic effect of the secondary amine.^64^ Thus, as expected, on the way towards the simplexity state, the evolution of the fractions of the different library constituents depends on the kinetics until equilibrium has been reached.

### Reactional organization along oligoamine amine chains

**Fig. 4:**
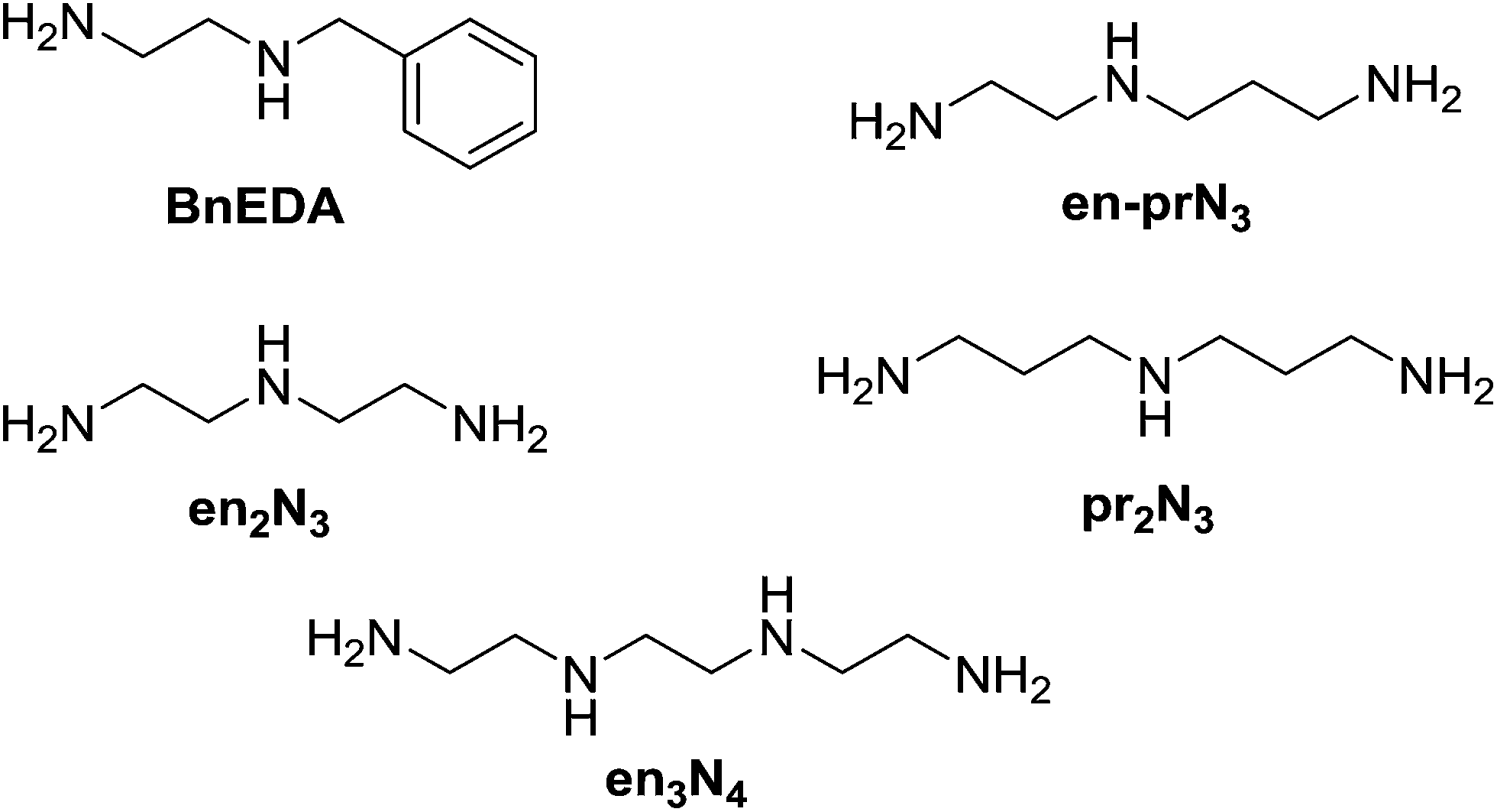
Structures and acronyms of polyamines used in the multivalency-based sorting experiments.

Selection in aldehyde–amine DCLs described above arises from the preference of a given aldehyde for its matching amine partner through equilibration involving reaction both with different amines and with different sites in an oligo(poly)amine. Combining different amine structural motives within one molecule opens the way to intramolecularly organise aldehyde residues along an oligo(poly)amine chain in a given sequence. Thus, using the described “matching pairs” a multivalent polyamine molecule containing both primary amine end groups and secondary amines along the chain could serve as the organisational scaffold for the positioning of aldehyde residues under functional recognition (Fig. 4).^4^,^57^,^65^

**Fig. 5:**
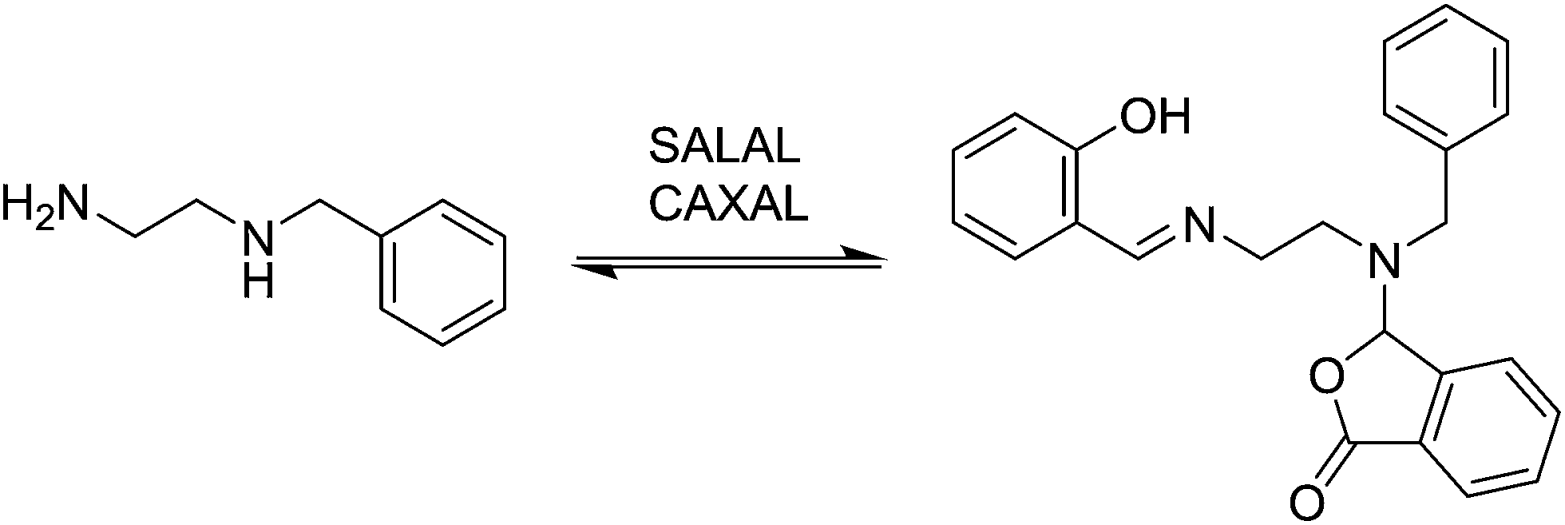
Simultaneous reaction of **BnEDA** with the imine-forming **SALAL** and the lactone-forming **CAXAL** with formation of the expected imine–lactone product in about 70% conversion. The imine is formed on the primary amine group and the lactone on the secondary one.

In the simplest case of *N*-benzylethylenediamine (**BnEDA**), the chain has one primary and one secondary nitrogen, expected to represent the reactivity of both **IPA** and **PIP** respectively. When an equimolar mixture of **SALAL** and **CAXAL** was reacted with 1 eq. **BnEDA** the dominant species in the solution (70%) was the expected imine–lactone: the imine of **SALAL** formed on the primary nitrogen and the lactone of **CAXAL** closed on the secondary nitrogen (Fig. 5, see ESI, Section 3.5,† for details). This predominant formation of a single product is remarkable given the fact that the mixture can in principle generate a large number of other species.

**Fig. 6:**
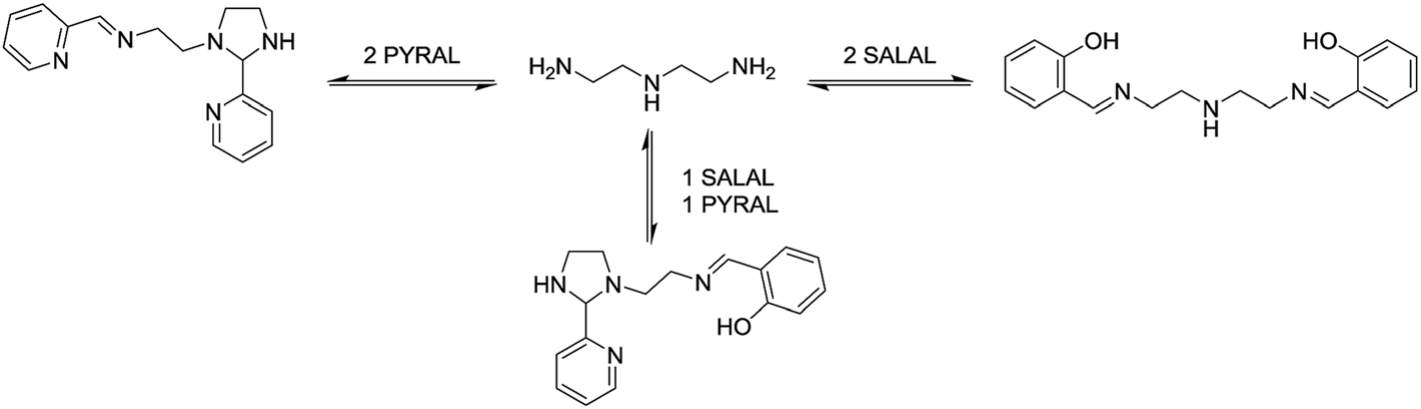
Selective intramolecular organization of two aldehyde residues *via* imine formation of **SALAL** and aminal formation of **PYRAL** affording the imine–aminal (bottom). Similar results are obtained with ethylene or propylene spacers between nitrogen atoms.

With extended chains with three nitrogen sites, such as diethylenetriamine (**en_2_N_3_**) or bis(3-aminopropyl)amine (**pr_2_N_3_**), selective formation of aminals with **PYRAL** can be assessed. Closure of the aminal ring bridges two nitrogen sites, terminal and central, while the third one, the other primary amine, is available for imine formation. Thus, when either of the triamines was reacted with 2 eq. of **PYRAL**, the imine–aminal was the major product formed. In contrast, when the triamines were reacted with 2 eq. of **SALAL**, the terminal bis-imine was the only product. Remarkably, reaction of the two aldehydes mixed in 1 : 1 ratio with the two triamines, resulted in selective formation of the expected products, the **SALAL**-imine and **PYRAL**-aminal, with respectively 77% and 84% conversion for **en_2_N_3_** and **pr_2_N_3_** (Fig. 6).

**Fig. 7:**
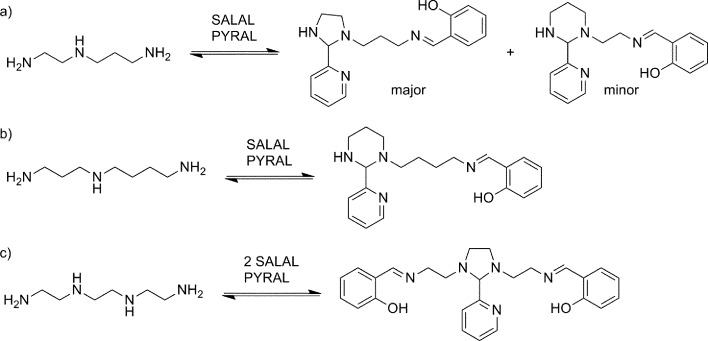
Reactional selectivity with multivalent polyamines: (a) reaction of **en-prN_3_** with a mixture of **SALAL** and **PYRAL** (1 eq. each) gave a mixture of two imine–aminal isomers; (b) spermidine afforded the expected imine–aminal in 71% conversion; (c) **en_3_N_4_** gave with about 60% conversion, the product bearing imines at the termini of the chain, accompanied by the aminal of **PYRAL** bridging the two central secondary amine sites.

The non-symmetric triamines **en-prN_3_** and spermidine were also examined in the reaction with the two aldehydes. When **en-prN_3_** was reacted with 1 eq. of **PYRAL** and 1 eq. of **SALAL** the desired imine–aminal species with five membered aminal ring formed in 85% conversion, with trace amounts of six-membered isomer. When spermidine was reacted under the same conditions, only one size of the aminal ring can be formed as seven-membered rings are much more difficult to form compared to the six-membered ones, and indeed the NMR spectrum revealed that the six-membered **PYRAL** aminal bearing the **SALAL**-imine on the C_4_-arm was formed in overall conversion of 71% (Fig. 7a, see ESI, Section 3.5,† for details).

In a further extension to four amino sites, the reaction of triethylenetetramine (**en_3_N_4_**) with 2 equivalents of **SALAL** gave a complex mixture of products: four different imine signals (three sharp and one broad) and three aminal peaks were observed in the NMR spectrum, corresponding to all possible combinations of imine–aminal structures formed in an essentially statistical fashion. However, when 1 equivalent of **PYRAL** was added to the mixture, preferential formation of its aminal on the middle secondary amino groups enhanced the formation of the imines of **SALAL** at the extremities leading to an equilibrium conversion of about 60% (Fig. 7c).

These experiments clearly demonstrate that despite the ambiguous reactivity of both the amine sites and the aldehydes (imine–aminal-lactone equilibria), the system of higher complexity involving all components led to a pronounced competition-enhanced selectivity towards a preferred species, as compared to less complex mixtures. These results provide a remarkable illustration of selectivity amplification, *i.e.* simplification induced by an increase in complexity.

## Selective dynamic protection of amino groups by reaction with aldehydes

Specific reversible reaction of different carbonyl reagents with different amino groups may offer a strategy that can be exploited for the selective dynamic protection of amines. The synthesis of complicated multifunctional molecules often requires to perform a selective reaction with a given functional group in presence of other similar groups. Such specific addressing of a given group in complex molecules is enabled by the use of protecting groups (PG).^66^,^67^ However, introduction and removal of a PG necessitates two more synthetic steps accompanied by purification procedures. It is therefore of much interest to develop “protecting-group-free” synthetic methodologies.^68^,^69^ Selective dynamic protecting groups (DPGs) could in principle offer an attractive intermediate approach whereby the desired functional group would be protected directly in the reaction mixture under thermodynamic control without isolation and purification, yet the dynamic nature provides reversibility for comparatively easy dynamic deprotection, for instance *via* transimination. Selective DPGs can be fully complementary to traditional PGs in organic synthesis^66^ as there are several types of dynamic linkages which have been shown to be orthogonal to each other,^33^–^36^,^70^ thus providing a pool of reagents for different functional groups.^71^,^72^ Together with dynamic kinetic resolution,^45^ the present work demonstrates the contribution that the implementation of Dynamic Covalent Chemistry (DCC) can make to the field of organic synthesis.

Amine protection with carbonyl compounds enables C-alkylations,^73^–^76^ O-alkylations^77^,^78^ or C=C double bond dihydroxylation.^79^ As protecting aldehydes, salicylaldehyde^80^–^83^ and formaldehyde^84^–^86^ have been used for selective imine or aminal formation respectively followed by the typical acidic hydrolysis work up.^73^–^76^ Deprotection can be effected by transimination on application of hydrazides^87^ or hydroxylamine derivatives.^82^,^88^

### Selective derivatisation of amines

#### Selective dynamic protection of primary amino groups

Common amine protection groups such as Boc, Cbz, Fmoc, *etc.* are introduced by reactions presenting often low selectivity among different amine sites using the corresponding chloroformate or anhydride. A selective version of this protocol employs trifluoroacetate protection of primary amine,^89^ but removal of the CF_3_CO group may be problematic and therefore lowers overall yields in these reactions. To explore the application of a DPG strategy, we first examine the case of *N*-methyl-1,3-diaminopropane (**MeDAP**). It contains both a primary and a secondary amino group and can, in principle, form aminal with an aldehyde thus comprising possible challenges in selective derivatisation of polyamines. Previous results have shown that **SALAL** forms selectively imines as the thermodynamic product with primary amines, as was confirmed also for **MeDAP** (quantitative conversion in less than 2 hours). The imine formation strongly differentiates the two nitrogen sites in their reactivities: while the secondary amino group is not altered, the primary one is engaged in the imine, which presents in addition a hydrogen bond with the neighbouring OH group. As a result, only the secondary amino group is free to react effectively with electrophiles such as acyl chlorides or isocyanates giving the corresponding amides or ureas while the imine function is not affected.

**Fig. 8:**
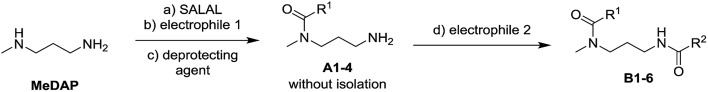
Illustration of the use of the DPG strategy for selective derivatization. Selective acylation of **MeDAP** is enabled by the selective imine formation using **SALAL** to protect the primary amine in presence of a secondary one. The reaction is under thermodynamic control which eliminates the need for purification of the protected intermediate. The dynamic nature of the imine bond also allows for easy protecting group removal and the resulting mono-derivatised **A1–4** can be reacted “one pot” with a second electrophile to give doubly derivatized products **B1–6**. The imine formation of **SALAL** directs the selectivity of the first acylation and thus the reaction sequence. Complete conversion of the imine exchange reaction (used in the deprotection step) provides acylhydrazone and oxime derivatives of **SALAL**, which are under given conditions benign byproducts. The sequence of operations allows in principle for multi-step reaction performed “one pot” and requiring purification only after the last step.

The dynamic nature of the reversible imine bond forming reaction gives the opportunity for facile protecting group removal in conditions which do not affect acylated amines or similar derivatives. Imine hydrolysis under acidic conditions can be replaced by much milder thermodynamically driven imine exchange. To this end, addition of a hydrazine, a hydrazide or an alkoxyamine leads to cleavage of the imine to give a hydrazone, an acylhydrazone or an oxime (respectively) of the carbonyl partner in quantitative conversion with liberation of the free primary amino group. The double implementation of the features of DCC gives the advantage to perform the three-step reaction sequence, protection–derivatisation–deprotection, in a “one pot” fashion without the need for isolation and purification of the intermediates (Fig. 8). A number of such processes have been explored (Fig. 8; Table 1). To be successful, this selective DPG approach requires of course high imine formation, high transimination on deprotection, as well as stability of the imine in the conditions used for the derivatization. Comprehensive method optimisation, synthetic details and full characterisation of all products are provided in the ESI, Section 4.† In the following text, the reported yields represent isolated amount of pure products.

**Table 1:** Products and isolated yields of the selective derivatisation of **MeDAP** using **SALAL** as protecting group for the primary amine group. Deprotection was effected by imine exchange and the reactions were conducted in “one pot” fashion followed by a single isolation and purification step. Cbz-GlyONp = *N*-benzyloxycarbonyl-glycine-*p*-nitrophenyl ester; Boc-LeuOSu = *N-t*-butyloxycarbonyl-leucine-*N*′-hydroxysuccinimide ester; Boc-AlaOSu = *N-t*-butyloxycarbonyl-alanine-*N*′-hydroxysuccinimide ester; CbzCl = benzyl chloroformate

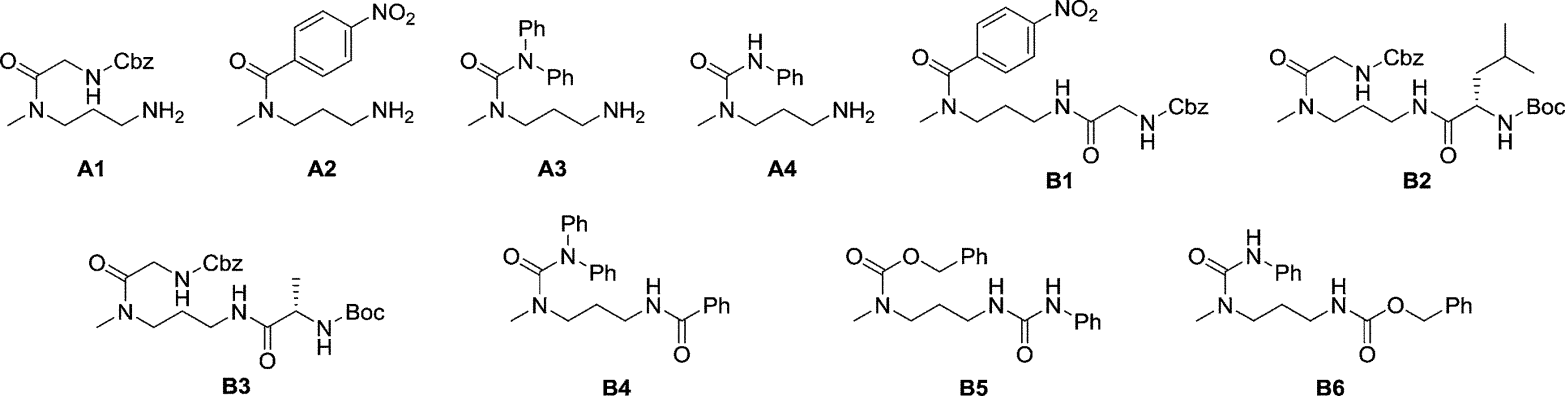					
Ref.	Electrophile 1	Electrophile 2	Solvent	Deprotecting agent	Yield
**A1**	Cbz-GlyONp	—	CH_3_CN	BnONH_3_Cl	82%
**A2**	*p*NO_2_PhCOCl	—	CH_3_CN	BnONH_3_Cl	69%
**A3**	Ph_2_NCOCl	—	CH_3_CN	PhCONHNH_2_	70%
**A4**	PhNCO	—	CH_3_CN	BnONH_3_Cl	86%
**B1**	*p*NO_2_PhCOCl	Cbz-GlyONp	CH_3_CN	PhCONHNH_2_	78%
**B2**	Cbz-GlyONp	Boc-LeuOSu	CH_3_CN	PhCONHNH_2_	83%
**B3**	Cbz-GlyONp	Boc-AlaOSu	CH_3_CN	PhCONHNH_2_	82%
**B4**	Ph_2_NCOCl	PhCOCl	EtOH	BnONH_3_Cl	75%
**B5**	CbzCl	PhNCO	CH_3_CN	PhCONHNH_2_	71%
**B6**	PhNCO	CbzCl	EtOH	BnONH_3_Cl	82%

In the reaction of **MeDAP** with the *p*-nitrophenyl activated ester of protected glycine (Cbz-GlyONp) using dynamic protection by 1 eq. **SALAL**, the *N*-Cbz-glycyl substituent was introduced on the secondary amine, giving isolated yield of 82% for the full three-step one-pot reaction sequence. Importantly, when the reaction was performed without the addition of **SALAL**, it afforded 85% isolated yield of a product consisting of a mixture of regioisomers due to unsufficient difference in reactivity of the two nitrogen atoms. The regioisomers were present in 2 : 1 ratio in favour of the product of acylation on the secondary amine. The product of double acylation of **MeDAP** was isolated as well in about 6% yield. We explored the versatility of the protocol by varying the reagents, the solvent and also the nature of the deprotecting agent. The results are summarized in Table 1 (entries **A1–A4**). Good isolated yields in the range of 69–86% of the desired products were obtained after the complete three-step reaction sequence.

The operation of DCC under thermodynamic control has been exploited here for the protection and deprotection steps. The establishment of equilibrium offers the possibility to perform sequences of reactions in systems of increasing complexity. Thus, the equilibrium mixtures in the previous experiments before isolation of products consist of the oxime or acylhydrazone of **SALAL** together with a **MeDAP** derivative displaying a free primary amino group (complete conversion in deprotection). We have therefore investigated the possibility to perform a controlled sequential derivatisation in a “one pot” fashion (Fig. 8), in which the imine formation by **SALAL** directs the sequence of derivatization to give products bearing the residue introduced first on the secondary amine group and the second one on the primary amine site. The reaction was repeated for various combinations of electrophiles, in two solvents and with two deprotecting agents giving yields of the desired products in the range of 71–83% (92–95% per step, Table 1 entries **B1–B6**). In contrast, when the reaction was performed in absence of the **SALAL**-imine protecting group using *N*,*N*-diphenylcarbamoyl chloride and benzoyl chloride as electrophiles, a mixture of all four possible products was obtained in yields from 16 to 34% (details in the ESI, Section 4.1.1.9†).

#### Inverted sequence of derivatisation

**Fig. 9:**
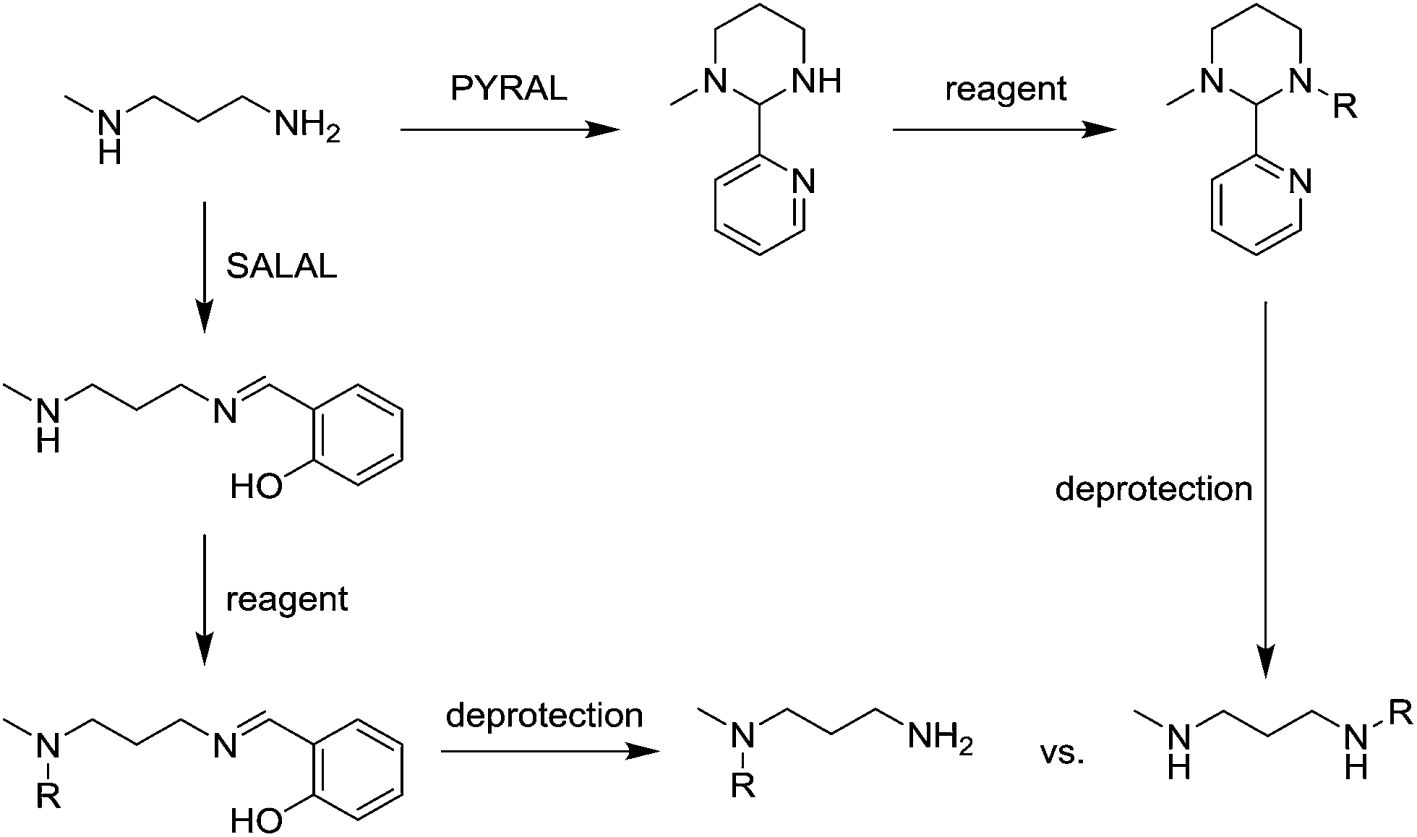
Representation of the generation of opposite sequences of substitution on consecutive double acylation of **MeDAP** when either **SALAL** or **PYRAL** is used as dynamic protecting group. Opposite regioisomers are obtained as a result of the preferential formation of imine and aminal condensation products by **SALAL** and **PYRAL** respectively. Both imines and aminals are formed reversibly which allows for mild deprotection with hydroxylamine or hydrazide derivatives.

In contrast to **SALAL**, which yields selectively imines, **PYRAL** preferentially gives aminals.^53^ Aminal formation between a primary and a secondary amine site transforms the latter into a tertiary amine and the former into a secondary amine site, which thus is available for reaction with an electrophile. As a consequence, acylation leads to a regioselectivity opposite to that obtained when **SALAL** is used, resulting in an opposite sequence of acyl groups in the case of sequential double acylation. Such a process has been performed with **MeDAP** as shown in Fig. 9.

When **PYRAL** was mixed with **MeDAP**, efficient formation of the aminal within 2 hours was confirmed by NMR. The equilibrated solution was then treated with phenyl isocyanate forming the urea moiety and the protecting group was removed by the exchange reaction with *O*-benzylhydroxylamine which converts the aminal of **PYRAL** completely to the corresponding oxime. The resulting product **C1** was isolated in 74% yield, accompanied with 2% of the opposite regioisomer (Table 2). It is thus possible to perform a double derivatisation of the diamine in a “one pot” fashion by addition of a second electrophile after deprotection. To this end, we have used phenyl isocyanate as the first electrophile and the activated ester of *N*-protected phenylalanine or benzyl chloroformate in the second acylation (isolated yields 76 and 74%, respectively), demonstrating the potential of the approach implementing dynamic protecting groups and mild deprotection procedures compared to conventional protecting groups used in peptide synthesis.

**Table 2:** Inverted selectivity of derivatisation of **MeDAP**. Formation of the six-membered aminal ring by the condensation with **PYRAL** leaves only the terminal amino group available for the reaction with electrophiles. Isolated yields for one pot multi-step procedures. Boc-PheOSu = *N-t*-butyloxycarbonyl-phenylalanine-*N*′-hydroxysuccinimide ester; CbzCl = benzyl chloroformate

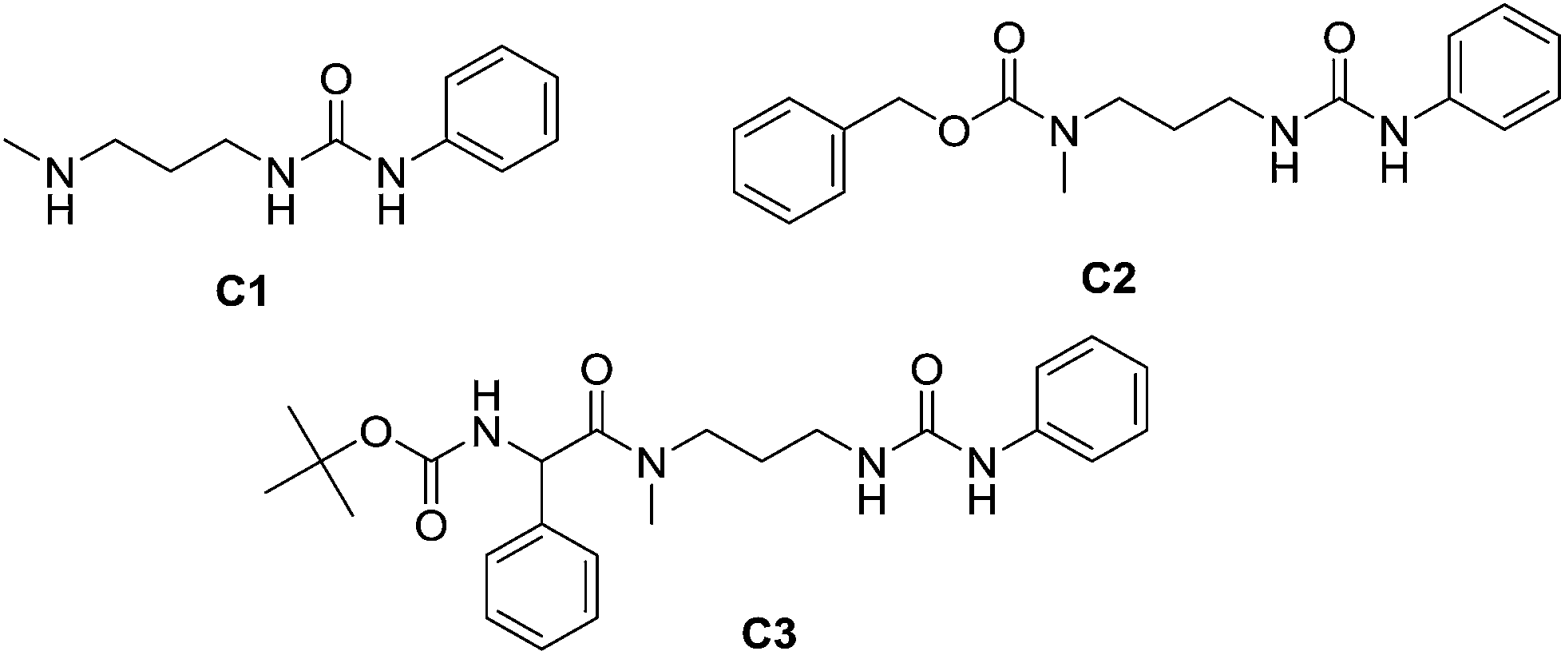					
Ref.	Reagent 1	Reagent 2	Solvent	Deprotecting agent	Yield
**C1**	PhNCO	—	CH_3_CN	BnONH_3_Cl	74%
**C2**	PhNCO	CbzCl	CH_3_CN	BnONH_3_Cl	74%
**C3**	PhNCO	Boc-PheOSu	CH_3_CN	BnONH_3_Cl	76%

**Fig. 10:**
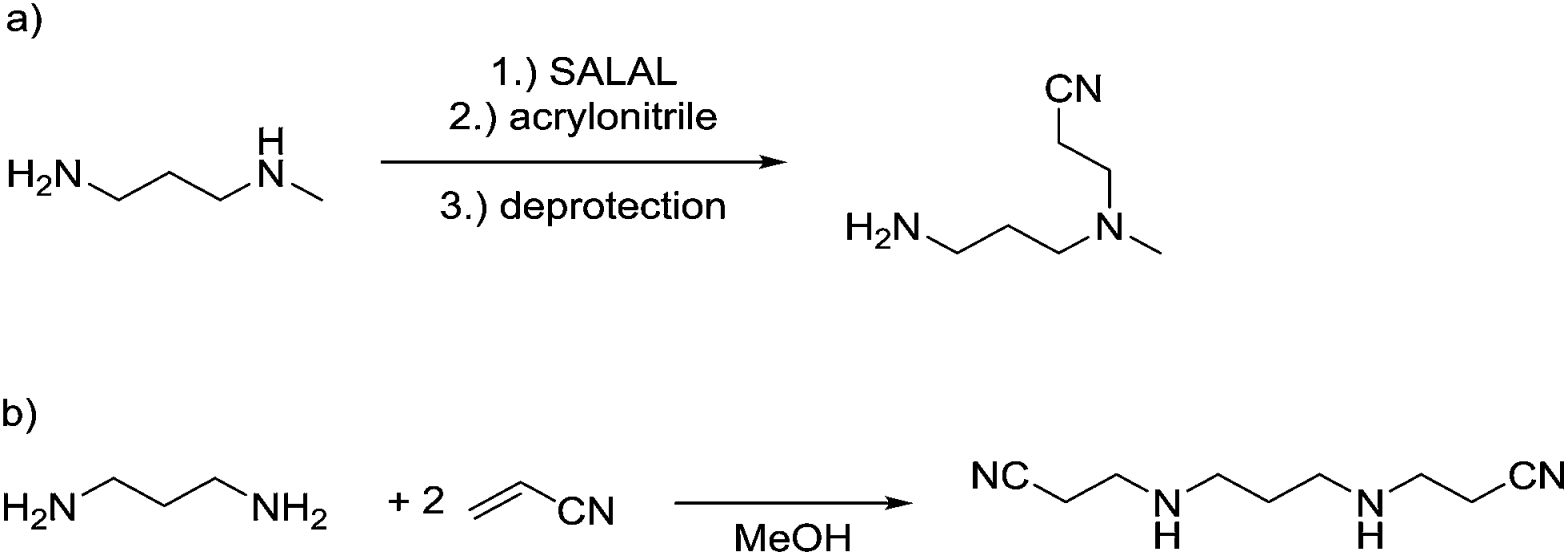
(a) Michael addition of acrylonitrile proceeds faster on the primary amino group. Selective reaction of the secondary amino group can be achieved by protection with **SALAL**. (b) Double Michael reaction of acrylonitrile (2 eq.) with 1,3-diaminopropane proceeds with complete selectivity on primary amino groups in full conversion without any protecting group.

The reaction employing **PYRAL** protection *via* aminal formation was studied with several different electrophiles used in the first acylation, but the isolated product always consisted of the diamine derivatised on the secondary amine (due to aminal–imine equilibrium, see above). Even after extensive optimisation of reaction conditions (details in the ESI, Section 4.1.3†) and replacement of **PYRAL** with formaldehyde, reported in the literature as aminal forming reagent,^84^,^85^ the terminal regioisomer was only obtained with phenyl isocyanate. Literature reports describe the use of aminal forming aldehyde to drive the selectivity of the Michael addition of acrylonitrile to polyamines.^85^,^90^ In this vein, **MeDAP** was reacted first with 1 eq. of **PYRAL** (or **SALAL**) and then 1 eq. of acrylonitrile was added. Formation of the product of Michael addition (Fig. 10a) was followed by NMR (at r.t. in *d*_4_-methanol) revealing that the reaction time in presence of **PYRAL** was much longer (35% conversion after 24 hours) than in the case of **SALAL** protected version (quantitative in 24 hours). Moreover, while **SALAL** drove the reaction with full selectivity for the addition on the secondary nitrogen, the reaction mixture with **PYRAL** consisted of both regioisomers in ratio 1 : 2.5 in favour of the same product as with **SALAL** (due to the aminal–imine dynamic interconversion). When the acrylonitrile Michael addition was repeated without any protecting aldehyde, again a mixture of products was obtained, but in this case favouring the addition on the primary amine. This finding indicates kinetic resolution between primary and secondary amines in the acrylonitrile Michael addition, which was supported by the reaction of 1,3-diaminopropane with 2 eq. of acrylonitrile providing a quantitative yield of the *N*,*N*′-bis(2-cyanoethyl)-1,3-diaminopropane (Fig. 10b, details in the ESI, Section 4.4†).^91^ In conclusion, the **PYRAL** protection is a suitable approach in the case of highly reactive isocyanate species, presumably because it reacts faster than is the rate of intramolecular aminal–imine interconversion. In the case of acyl chlorides this equilibrium, although strongly shifted towards the aminal, leads to non-selective derivatisation of both amino groups. Acrylonitrile, reacts preferentially with primary amines and **SALAL** protection is needed to achieve the selective reaction on the secondary nitrogen.

### Selective derivatisation of oligoamines

**Fig. 11:**
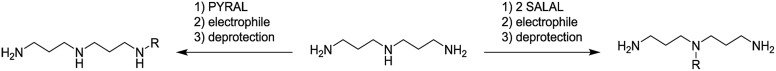
Selective derivatisation of polyamines in the case of **pr_2_N_3_**. **en_2_N_3_** gives similar results. (Right) End-capping of the polyamine chain with **SALAL** protection drives the reaction to the central secondary nitrogen, whereas (left) linking two nitrogens in the aminal form of **PYRAL** leads to selective derivatisation of only one of the terminal primary amine.

Selective dynamic protection of amino groups was further explored with challenging polyamine substrates. To demonstrate the principle and to draw a comparison with known alternative protocols, we have examined the monoderivatisation of diethylenetriamine **en_2_N_3_** at the central secondary amine group. The traditional protection/reaction/deprotection approach^89^ employs trifluoroacetyl protection of the terminal NH_2_ functions, followed by reaction with Boc_2_O and removal of the trifluoroacetate groups by reflux in ammonia solution with overall 63% yield including at least two chromatographic purification steps. In the present case, **SALAL** is used as the protecting agent (2 eq.) and the reaction is performed at room temperature, in “one pot” fashion, with a single isolation and purification step giving the desired product in an overall yield of 80% (see ESI for the synthetic protocol†). Selective DPGs offer large versatility which is not easily available with conventional protecting groups. If **pr_2_N_3_** is protected by 1 eq. of **PYRAL** forming the aminal and then reacted with an electrophile, selective monoderivatisation of one of the termini is achieved in good yields (70%, Fig. 11, see ESI, Section 4.5,† for synthetic details). In this case, the **PYRAL** protection is crucial since reproducing the reaction without the protecting aldehyde led to a complex mixture of all possible products in essentially statistic ratio.

**Fig. 12:**
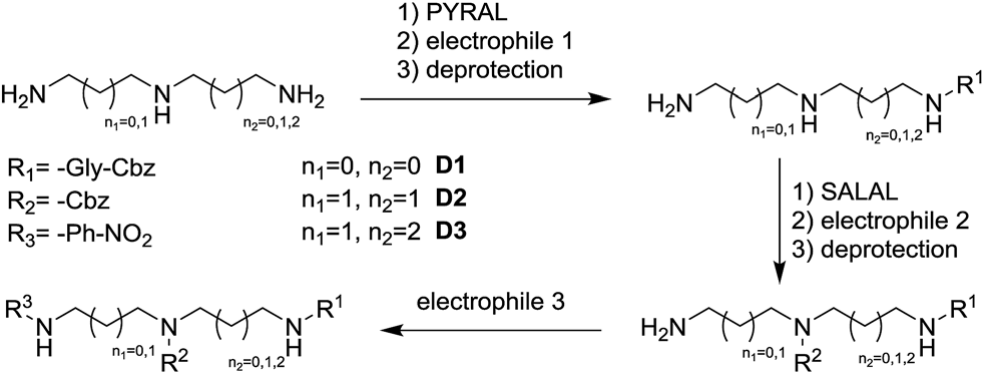
Selective derivatisation of oligo(poly)amines is enabled by preferential product formation of a given aldehyde with the amine under thermodynamic control. Mild deprotection by imine exchange reaction, again thermodynamically driven, allows performing a sequence of three derivatisation steps without isolation and purification of intermediates.

Finally, the sequential derivatisation shown above for diamines was also investigated with the biologically relevant triamine spermidine. In this case, the reaction sequence started with protection by 1 eq. of **PYRAL** forming the aminal and thus leaving only one primary amino group available for the subsequent reaction with the activated ester of an amino acid (Cbz-GlyONp). The protecting group was removed by exchange reaction with benzyloxyamine and without isolation, the subsequent protection by 1 eq. of **SALAL** was introduced. The selective imine formation at the other terminal NH_2_ group of the starting polyamine left the central secondary amine free, thus allowing for selective derivatisation in the middle of the chain by benzyl chloroformate. Deprotection was again performed by imine exchange with benzyloxyamine and the last remaining nitrogen atom was thereafter derivatized using *p*-nitrobenzoyl chloride (Fig. 12). This 7-step, one-pot reaction sequence performed at r.t. under ambient atmosphere afforded after a single purification step the desired triply derivatized product in 31% yield (85% calc. per step), demonstrating the potential of dynamic selective protecting groups in organic synthesis.

The presented strategy of selective DPGs showed remarkable versatility when **SALAL** was used as the protecting agent. On the other hand, inverted sequence of derivatisation by employing **PYRAL** was limited to cases in which the acylating reagent reacts faster than the rate of equilibration of the protecting group between several species. These two approaches are thus complementary and when combined, provide a powerful tool to perform selective and/or sequential functionalization of polyamines.

## Conclusions

Dynamic covalent chemistry (DCC) operates at thermodynamic equilibrium achieved by component exchange through reversible covalent reactions. The condensation of carbonyl compounds with amines is of special interest as it can result in the formation of N–C–N aminals in addition to the usual C=N imines, depending on the nature of the carbonyl component. It offers thus a richer palette of constituents that may be exploited towards the design of selective reaction pathways of interest for organic synthetic strategies. It allows for substrate selectivity in mixtures of amines as well as for the programming of reaction sequences towards the control of regioselective positioning of carbonyl residues along an oligo(poly)amine chain. These features lead to the concept of *selective dynamic protecting groups*, whereby primary and secondary amine functional groups may be reversibly derivatized in processes presenting selectivity between different amine compounds as well as between different amino sites within a polyamine *via* a network of underlying equilibrating reactions.

The selective DPG concept has been exploited towards the regioselective derivatization of various amines with different reagents ranging from isocyanates to activated esters of amino acids. The establishment of equilibrium in the reaction of the substrate with the protecting group as well as in the deprotection step eliminates the need for isolation and purification of intermediates and allows to run reactions in “one-pot” fashion. Furthermore, the thermodynamic control over the reversible condensations opens the possibility to perform sequences or networks of reactions in systems of increasing complexity, as it was shown in the case of selective sequential derivatisation of polyamines. Exploitation of other reversibly formed species, such as acetals or boroxines, can provide useful alternatives in total synthesis of complex products. Altogether the present work represents an extension of the thermodynamically driven DCC, implementing *dynamic organic reactivity*, into the traditionally kinetically governed realm of organic synthesis and provides ground for further exploration of its potential.

Finally, in a broad perspective, the data presented provide a remarkable illustration of simplification within a given constitutional dynamic library, induced by an increase in complexity, which leads to enhanced competition within the set of equilibrating constituents undergoing component exchange *via* a network of interconnected reactions engaged in agonistic and antagonistic relationships with feedback loops. In general terms, higher complexity results in simplification through competition.

## Supplementary Material

Supplementary information
