# Supplementary material for: Competition-driven selection in covalent dynamic networks and implementation in organic reactional selectivity
Source: Chem Sci. 2016 Feb 10;7(5):3215–26. doi: 10.1039/c5sc04924e (PMC6005339; doi:10.1039/c5sc04924e)
Supplement: Supplementary file 1 [file SC-007-C5SC04924E-s001.pdf]

Electronic Supplementary Material (ESI) for Chemical Science.
This journal is © The Royal Society of Chemistry 2016

SUPPLEMENTARY INFORMATION

COMPETITION-DRIVEN SELECTION IN COVALENT DYNAMIC NETWORKS AND IMPLEMENTATION IN ORGANIC REACTIONAL SELECTIVITY

*Dr. Petr Kovaříček,[‡] Dr. Anne Claire Meister,[†] Dr. Karolína Flídrová, Dr. Rafel Cabot,[$] Kateřina Kovaříčková,[§] Prof. Jean-Marie Lehn**

Institut de Science et d'Ingénierie Supramoléculaire, Université de Strasbourg, 8 allée Gaspard Monge, 67000 Strasbourg, France

Tel. : +33 (0)3 68 85 51 44, Fax : + 33 (0)3 68 85 51 40

**Email:* lehn@unistra.fr

[‡] Current address: Laboratory of Organic Chemistry and Functional Materials, Department of Chemistry, Humboldt-Universität zu Berlin, Brook-Taylor-Straße. 2, 12489 Berlin, Germany

[†] Current address: Merck KGaA, Frankfurter Straße 250, 64293 Darmstadt, Germany

[$] Current address: Department of Chemistry, University of Cambridge, Lensfield Road, CB2 1EW Cambridge, United Kingdom

[§] Current address: Institute of Chemical Technology Prague, Department of Research and Development, Technická 5, 160 00 Prague, Czech Republic

CONTENTS

1. Instrumentation ... 2
2. Selection in dynamic imine libraries ... 2
   2.1 Quantification of selection ... 4
   2.2 Simplexity: selection in dynamic reaction networks ... 6
      2.2.1 Example of quantification of simplexity selection ... 7
3. Dynamic combinatorial libraries ... 8
   3.1 The [1x1] aldehyde-amine mixtures ... 8
   3.2 The [1x2] and [2x1] aldehyde-amine mixtures ... 12
   3.3 The [2x2] aldehyde-amine mixtures ... 14
   3.4 Selection matrices for the full [3x3] library ... 21
   3.5 Multivalency experiments ... 24
4. Dynamic selective protecting groups ... 27
   4.1 Optimization of reaction conditions – general tendencies and results ... 27

4.1.1 Bis-reaction with SALAL 28
4.1.2 Mono-reaction with SALAL 30
4.1.3 Bis-reaction with PYRAL 31
4.1.4 Mono-reaction with PYRAL 32
4.2 Syntheses 35
4.2.1 Examples of the SALAL protection procedure 35
4.2.2 Examples of the PYRAL protection procedure 35
4.2.3 Synthetic protocols 36
4.3 NMR spectra of key intermediates and experiments 44
4.3.1 Intermediates in SALAL and PYRAL protection 44
4.4 Acrylonitrile Michael addition 46
4.5 Selective derivatisation of triamines 48
4.5.1 NMR equilibration study 48
4.5.2 Syntheses 53
5. References 58

1. Instrumentation

NMR spectra were recorded on Bruker Avance 400 (400.14 MHz for ^{1}H and 100.62 MHz for ^{13}C), Bruker Avance III plus 400 (400.34 MHz for ^{1}H and 100.67 MHz for ^{13}C), Bruker Avance I 500 (500.13 MHz for ^{1}H and 125.61 MHz for ^{13}C) and Bruker Avance III 600 (600.13 MHz for ^{1}H and 150.90 MHz for ^{13}C) NMR spectrometers. Spectra measured in aqueous solutions were referenced on 2,2,3,3-tetradeutero-3-trimethylsilylpropionic acid (δ=0). The other spectra were referenced on residual solvent signal according to Nudelman et al.[1]

Deuterated solvents were purchased from Euriso-TOP and used without further purification.

Reagents and solvents were purchased from Sigma-Aldrich, Alfa Aesar, Bachem, ABCR, STREM and Carlo Erba and used without further purification.

pH measurements were done on a Mettler Toledo Seven multimeter with glass electrode calibrated by standard buffer solutions from Sigma-Aldrich (4.01, 7.00 and 10.00). The values were not corrected for the deuterium isotope effect.[2]

Mass spectra were obtained on Bruker MicroTOF and HRMS on Bruker MicroTOF-Q, both with electrospray ionization. Nominal precision of the HRMS analysis is 10 ppm.

2. Selection in dynamic imine libraries

Sorting of dynamic combinatorial libraries is a common feature in the field of DCC, however the results are not directly comparable between different systems. We hereby offer an algebraic model

which represents the library in form of a matrix and we have also developed a system for quantification of the efficiency of the selection process. The model will be explained through the example of distillation sorting of an imine library, while extensions to different systems and generalizations will be introduced at the corresponding points.

The imine library is formed from $\boldsymbol{m}$ derivatives of aldehydes (which can be algebraically represented as vector $\mathbf{X}$) and the same number of amine derivatives (vector $\mathbf{Y}$) in equimolar amounts of all components. If the aldehydes are distributed as columns and amines as rows, a square matrix $\mathbf{XY}$ of possible products $\boldsymbol{x_i y_j}$ is obtained (Table 1). In principle, every possible combination in this matrix has the same mole fraction (population). When a selector is applied, such as distillation, redistribution of the product populations takes place, giving in the ideal case only a number $\boldsymbol{m}$ of products, while all other species are depleted (in the case of a total selection). If the aldehydes $\mathbf{X}$ and amines $\mathbf{Y}$ are initially sorted on the basis of boiling points, the matrix obtained after the selector is applied is an identity matrix, or it can be transformed to a diagonal identity matrix by swapping its rows/columns (Table 1).

	X_1	X_2	…	X_m
Y_1	1/m	1/m	…	1/m
Y_2	1/m	1/m	…	1/m
…	…	…	…	…
Y_m	1/m	1/m	…	1/m

selector →

	X_1	X_2	…	X_m
Y_1	1	0	…	0
Y_2	0	1	…	0
…	…	…	1	…
Y_m	0	0	…	1

*Table 1 A hypothetical selection experiment: matrix (formed from vectors **X** and **Y**) of initial populations (**I**) is transformed into the matrix of final products (**F**) by the action of a selector. The selector is such that it enhances the formation of $\mathbf{x_i y_i}$ adducts and disfavours formation of $\mathbf{x_i y_{j \neq i}}$ adducts. Typical example of such a selector is the distillation driven self-sorting of dynamic libraries.*

The selection process can be generalized. In an $\boldsymbol{n}$-component reaction,[1] each component is represented by a vector $\vec{R}_n$ of $\boldsymbol{m}$ reagents $\mathbf{X}$. By mixing the components in equimolar ratio we create a tensor $T_{R_{1\to n},m}$ of order $\boldsymbol{n}$, normalized such that $\sum_{R_{1\to n},m} T_{R_{1\to n},m} = m$. By the action of a selector, this tensor changes values of its elements (redistribution of reaction products) to give an identity tensor (in case of total selection) or a tensor that can be transformed to identity tensor by swapping the vectors, *i.e.* a tensor in which diagonal elements $T_{i_1 = i_2 = \ldots i_n} = 1$ and off-diagonal elements $T_{i_j \neq i_k} = 0$. It is important to point out:

[1] For heteromeric linkages, such as imine bond, typically n=2, like in the case of aldehyde-amine libraries, but examples of up to n=4 are demonstrated in the literature.[3] Generalization of the approach for homomeric linkages, such as disulfides, is straightforward.

1. If the selector is not an inherent part of the tensor (such as the distillation or oxidation process) reduction of the size (eliminating one reagent) of the tensor does not in principle affect the selection.
2. The size x of the vectors $\vec{R}_n$ can be in principle different giving a rectangular instead of a square tensor.
3. The selector might apply only on some of the $\vec{R}_n$ vectors, *e.g.* metal cation can change imine distribution without touching distribution of disulfides while pH changes the disulfide distribution without redistributing imine populations, etc.
4. The aforementioned algebraic description of the selection phenomenon can also be interpreted using graph theory, where each component vector would be represented as a column of compounds and each of its elements would have a connection to all compounds in neighbouring column. The thickness of the connection would represent the relative population in the mixture. This representation makes the agonistic-antagonistic relationships more obvious, but on the other hand quantification and deconvolution is less evident.

An important process is so-called "deconvolution" of a dynamic library. Deconvolution simply relates to removal of a given component from the mixture, e.g. removing one aldehyde and one amine and repeating the distillation. Thus to fully deconvolute the imine library, it is necessary to perform the distillation experiments on the libraries $m*m$, $(m-1)*(m-1)$,… to $1*1$, giving in total m independent experiments. It is noteworthy that for the general case of n-dimensional tensor this value is always m, regardless the dimensionality n. Also, it is possible to remove just one of the reacting partners, e.g. an aldehyde, and keep its amine counterpart in the distillation mixture. Such an experiment would be represented by a rectangular instead of a square matrix. In the following Sections, only the simplest case of a square 2D matrix is discussed for clarity. However, the extensions to the general case of a rectangular n-dimensional tensor can be envisaged.

2.1 Quantification of selection

Using the same example of the distillation sorted imine library, a system for quantification of the selection in two-component libraries can be proposed. The imines are formed by the general scheme A+B → C. Taking, for example, three aldehydes A and three amines B a [3x3] matrix of imines is created. This matrix before the distillation represents the initial composition of the library, and is therefore called I, and its elements $i_{i,j}$ represent the molar fraction (population) of each imine. After the distillation, we get a new matrix representing the molar fractions of obtained imines, called the final matrix F. The selection process can now be seen also as a matrix called the selector matrix S, defined by the formula

$$F = I + S$$

The selector matrix S can be used to quantify the selection. As seen in the schematic representation in Table 1, the elements of the diagonal have increased (by $(m-1)/m$) and the off-diagonal elements have decreased (by $-1/m$). In practise, the elements $s_{i,j}$ of the selection matrix S are obtained as the difference in mole fraction (population) of the products before and after the selector is applied. In principle, due to the mass conservation law, the sum of the elements of the selector matrix is 0. However, in practice all chemical processes lead to some loss (residue after distillation, incomplete reactions etc.), and therefore the sum of the elements $s_{i,j}$ of matrix S is a small negative number. Now, the selection index σ can be defined as follows

$$\sigma = \frac{\sum_{i,j}|s_{i,j}|}{2}$$

where the factor of 2 compensates for the fact that each decrease in population of a given product is accompanied by an increase of a different one. If the species in rows and columns are sorted such that after selection the positive (amplified) species are on the diagonal, then the positive elements (amplified species) are on diagonal, then the negative elements (depleted species) are off-diagonal, and the selection index is simply the sum of elements on the diagonal.

It is important to consider the possible values of $\boldsymbol{\sigma}$ by examining the limiting cases. In the case of a selector which doesn't change the population of the species, the elements of the selector matrix $\boldsymbol{s_{i,j}}$ are all equal to zero and thus also the value of the selection index $\boldsymbol{\sigma}$ is zero. In the case of an ideal selection, i.e. initial [3x3] matrix consists of equal amounts of all species and after selection only three products are obtained (Table 2), the selection index is equal 2 (or in general $\boldsymbol{m-1}$). In another case, where only three products form in the initial population but redistribute upon action of a selector to give three different products, the value of the selection index is 3 (or $\boldsymbol{m}$ for the general case).

	X_1	X_2	X_3		selector					X_1	X_2	X_3
Y_1	1/3	1/3	1/3		2/3	-1/3	-1/3		Y_1	1	0	0
Y_2	1/3	1/3	1/3	+	-1/3	2/3	-1/3	→	Y_2	0	1	0
Y_3	1/3	1/3	1/3		-1/3	-1/3	2/3		Y_3	0	0	1

Table 2 A schematic representation of an ideal selector and its corresponding selector matrix. An equally populated mixture of all possible products is transformed to only three selected species.

It is obvious that value of the selection index is a function of the size of the initial matrix, for a [2x2] matrix it takes values from 0 to 2, for a 4x4 it can be up to 4. To generalize the selection descriptor, the selection index can be transformed into a percentage value called the selection strength $\boldsymbol{\varepsilon}$ as follows: for $\boldsymbol{m}$ number of derivatives A or B, the values of the selection index $\boldsymbol{\sigma}$ from $\boldsymbol{0}$ to $\boldsymbol{(m-1)}$ define a range from 0 to 100 %, and values from $\boldsymbol{(m-1)}$ to $\boldsymbol{m}$ define the range from 100 to 200 % (Graph 1). Thus an ideal selector has strength of 100 %, a selector which inverts the populations has strength of 200 %, and a selector which does not influence the system has strength of 0 %. All other cases fall in between those values.

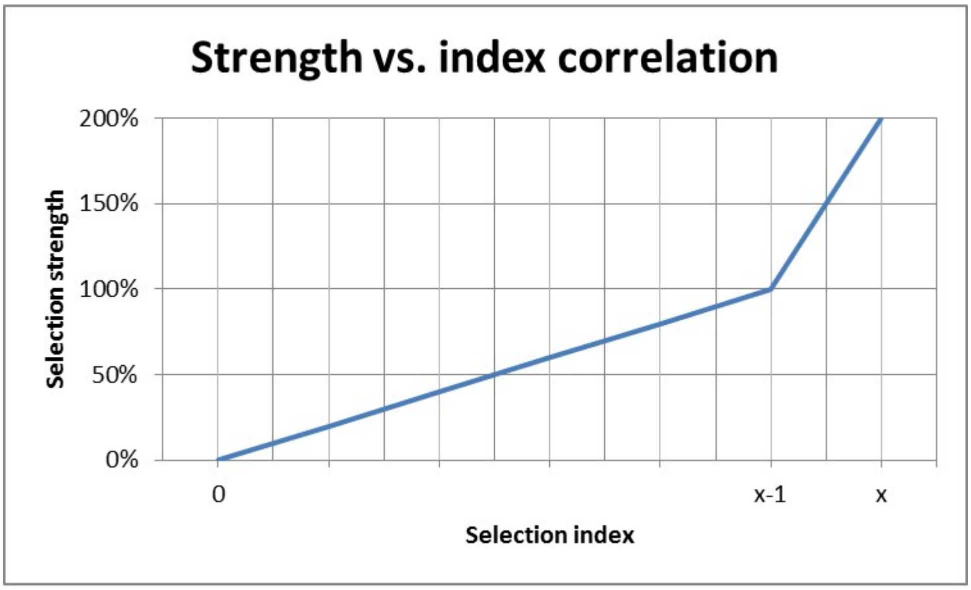

Graph 1 Graphical representation of the dependence of the selection strength on the selection index.

As for all descriptors, a single value does not completely describe the system but serves only for quick comparisons. The mathematical construction above enables single-value comparison of different selection systems.

2.2 Simplexity: selection in dynamic reaction networks

Considering dynamic reaction networks, which is discussed in the current work, the selection is driven by the complexity of the reaction mixture. In other words, the selection for a given product is a result of an increased number of species in the mixture. In such a case, the selector is inherent to the complex mixture, i.e. the feature of selectivity is encoded in the components. This property will be said to define the *simplexity*. If one or more components of the library are removed, the selection can be strongly perturbed or even completely lost. Therefore, to perform the deconvolution of such a system, all combinations of reagents must be experimentally explored requiring for $(2^m - 1)^n$ experiments. Clearly, the number of experiments rises exponentially as the system gets more complex and even just for a [3x3] library 49 experiments are required.

Quantification of the selection in the case of simplexity is complicated by the fact that the selector is inherently present and the matrix of initial populations $\boldsymbol{I}$ (as in the abovementioned case) is not available. In such case, the *deconvolution* of the full constitutional matrix is required. In practice it means that all combinations of reactants A are mixed with all combinations of reactants B. In the case of an imine library, it means that every single aldehyde and every single amine, as well as all combinations of two or more aldehydes or amines, are not added to the library and the equilibrium composition of the resulting mixture is determined.

The result of the deconvolution enables for construction of the selection matrix $\boldsymbol{S}$. Its elements $s_{i,j}$ are defined as

$$\boldsymbol{s_{i,j} = (f_{i,j} - \overline{\mu_{i,j,}})(1 - std_{i,j})}$$

where $s_{i,j}$ is the element of the selection matrix, $f_{i,j}$ is the population of the product in the full matrix (i.e. that where all constituents are present), $\overline{\mu_{i,j,}}$ is the mean of the population of this product

in all the deconvolution experiments (as described above) where this product can be formed (i.e. both its constituents are present) and $std_{i,j}$ is the standard deviation of the population of a given product through the deconvolution experiments. The term $(\boldsymbol{f_{i,j}} - \overline{\boldsymbol{\mu_{i,j}}})$ defines the result of a selection process (i.e. difference between initial and final mole fraction of the product as discussed above for the case of distilled library) and its value falls in between -1 and 1. The term $(1 - std_{i,j})$ reflects the variation of the population of a given product through the deconvolution and warrants a closer look. In a hypothetical case, a product $\boldsymbol{x_i y_j}$ is formed in 100 % yield when all the constituents of the library are present. But through the deconvolution experiments, its conversion is constantly 0 %. It seems logical that the selection for its 100 % formation requires large effort. Conversely, if its conversion varies greatly between 0 and 100 % through the deconvolution, it seems that the change to 100 % conversion would be easier. Therefore, for a constant conversion through the deconvolution experiment, the $std_{i,j}$ will be close to 0 and the whole term close to 1. For a fluctuating conversion it will be close to 0.5, thus reducing the contribution to the selection caused by this product. In other words, this term describes how great the propensity to form a given product is. With the selection matrix in hand constructed in according to the aforementioned procedure, it is possible to calculate the selection index and thereafter the selection strength, analogously to the case of an external selector described above. It is noteworthy that due to the $(1 - std_{i,j})$ term the sum of the elements of the selector matrix no longer needs to be zero. Also, due to this term, the selection strength in the simplexity selection should be lower than in the selection driven by an external force.

2.2.1 Example of quantification of simplexity selection

The complexity-driven selection described above can be quantified using the model introduced in the Section 2.2. The procedure of quantification is described in detail on the case of **SALAL** and **CAXAL** reacted with **IPA** and **PIP**, and the quantification results for the other selection experiments are also provided. In the full [2x2] experiment, only two species were observed: the imine of **SALAL** and the lactone of **CAXAL**. The final matrix is thus an identity matrix:

	SALAL	CAXAL
IPA	1	0
PIP	0	1

In the separate experiments, both of these products were also formed quantitatively:

	SALAL	
IPA	1	
[redacted]		

		CAXAL
PIP		1

Also the “not-matching” pairs were examined:

	SALAL	
PIP	0.30	

		CAXAL
IPA		1

When the two amines were reacted with only one of the aldehydes, a distribution of products and in the case of **SALAL** incomplete reaction was observed by NMR:

	SALAL	[redacted]
IPA	0.9	[redacted]
PIP	0	[redacted]

	[redacted]	CAXAL
IPA	[redacted]	0.61
PIP	[redacted]	0.39

Similarly, when the two aldehydes were treated with only one of the amines, a distribution of products in the case of **SALAL** was observed by NMR:

	SALAL	CAXAL
IPA	0.77	0.23
[redacted]	[redacted]	[redacted]

	SALAL	CAXAL
[redacted]	[redacted]	[redacted]
PIP	0	1

From the conversion values through the deconvolution the mean and its standard deviation can be calculated for each product:

$$x[SALAL + IPA] \in \{1, 0.9, 0.77\}, \bar{\mu} = 0.89, std = 0.12$$

$$x[SALAL + PIP] \in \{0.3, 0, 0\}, \bar{\mu} = 0.1, std = 0.17$$

$$x[CAXAL + IPA] \in \{1, 0.61, 0.23\}, \bar{\mu} = 0.61, std = 0.39$$

$$x[CAXAL + PIP] \in \{1, 0.39, 1\}, \bar{\mu} = 0.80, std = 0.35$$

From these values, the selector matrix can be constructed:

$$\begin{bmatrix} (1-0.89)(1-0.12) & (0-0.61)(1-0.39) \\ (0-0.1)(1-0.17) & (1-0.8)(1-0.35) \end{bmatrix} = \begin{bmatrix} 0.1 & -0.37 \\ -0.08 & 0.13 \end{bmatrix}$$

The sum of absolute values of the selector matrix divided by 2 gives a selector index of 0.34, which can be expressed as the selection strength of 34 %.

3. Dynamic combinatorial libraries

The establishment of equilibrium is of course a crucial point. The mixtures of components were monitored by NMR until no change in composition was detected. Afterwards, the sample was heated at 60 °C for 2 to 24 hours and again the composition of the mixture was determined. Finally, where appropriate, constituents were added to the mixture in different order with varying delays between additions (2 to 24 hours) and the evolution of the mixture composition was monitored. When these experiments showed identical final composition the equilibrium was indeed established and these values are reported.

3.1 The [1x1] aldehyde-amine mixtures

In an initial set of experiments, reactions between the individual reactants (one aldehyde and one amine) were studied in NMR tube in order to reveal the intrinsic reactivity of the various components. The reaction conditions have been fixed throughout the study using d_6-DMSO + 1 % D_2O as the solvent mixture (water was added to keep constant water content in the medium during the aldehyde-amine condensation), concentration of each reactant was 20 mM and the reaction was performed at room temperature unless explicitly stated otherwise. The discussed observations are reported at equilibrium which has been demonstrated by no further development of the NMR spectra in time.

The addition of one equivalent of **IPA** into a solution of **SALAL** in leads to the instantaneous (< 60 seconds), almost quantitative (> 98 %) formation of the correspondent imine as a single product. As shown in Figure 1, the reaction can be monitored using ^{1}H NMR spectroscopy, by following the decrease in the aldehyde peak (10.26 ppm) and the corresponding increase of the imine peak (8.58 ppm). A clear chemical shift change is also observed in all the signals in the aromatic region, compared to those of **SALAL**. In addition, another clear indication for the formation of the imine is the large downfield shift of the signal corresponding to the phenolic OH group of **SALAL**, due to intramolecular hydrogen bonding with the imine nitrogen.[4] Finally, a large downfield shift is also observed for the CH_2 group adjacent to the imine nitrogen (triplet at 3.61 ppm) compared to that of **IPA** (2.52 ppm, partially overlapped with solvent peak).

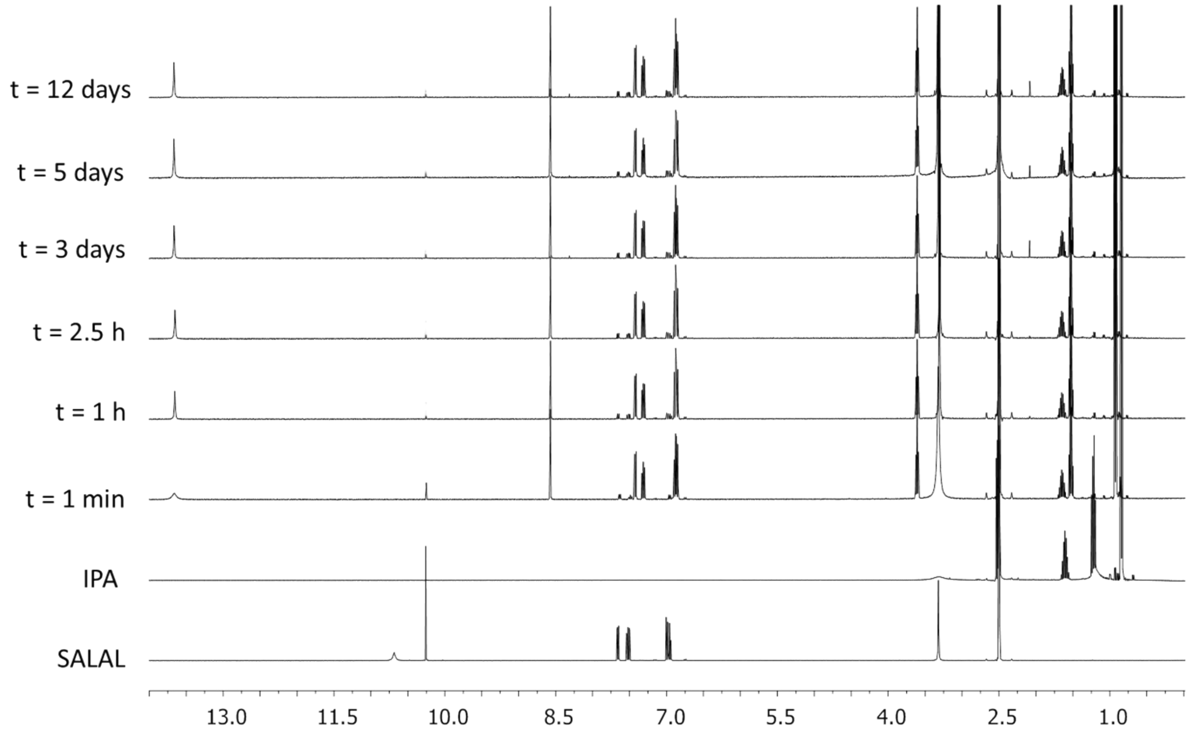

*Figure 1 ^{1}H NMR spectra of the individual reactants (**SALAL** and **IPA**) and the corresponding imine as the product of the reaction. Characteristic signals: aldehyde CHO 10.2 ppm, imine CH=N 8.6 ppm, imine N-CH_2 3.7 ppm, amine N-CH_2 2.5 ppm (overlapped with solvent signal). Aliphatic amine signals shift downfield upon formation of the imine.*

The reaction between equimolar amounts of **SALAL** and **PIP** proved to be more complicated. A colour change from pale to bright yellow occurs immediately upon addition of **PIP** into the solution containing **SALAL**, with a continuous increase in colour intensity, leading to dark orange/red after several days. The ^{1}H NMR of the reaction mixture (Figure 2) acquired immediately after the addition shows that the aminal is formed by the reaction of one molecule of **SALAL** with two molecules of **PIP**, consistently with the literature.[5] However, upon prolonged standing (> 2 days) new peaks appear as sharp singlets at 7.95 and 5.65 ppm as well as multiplets heavily overlapped with the residual water signal, which might be a result of oxidation by atmospheric oxygen. It is noteworthy that this reactivity was only observed in this particular experiment and only at very long reaction times.

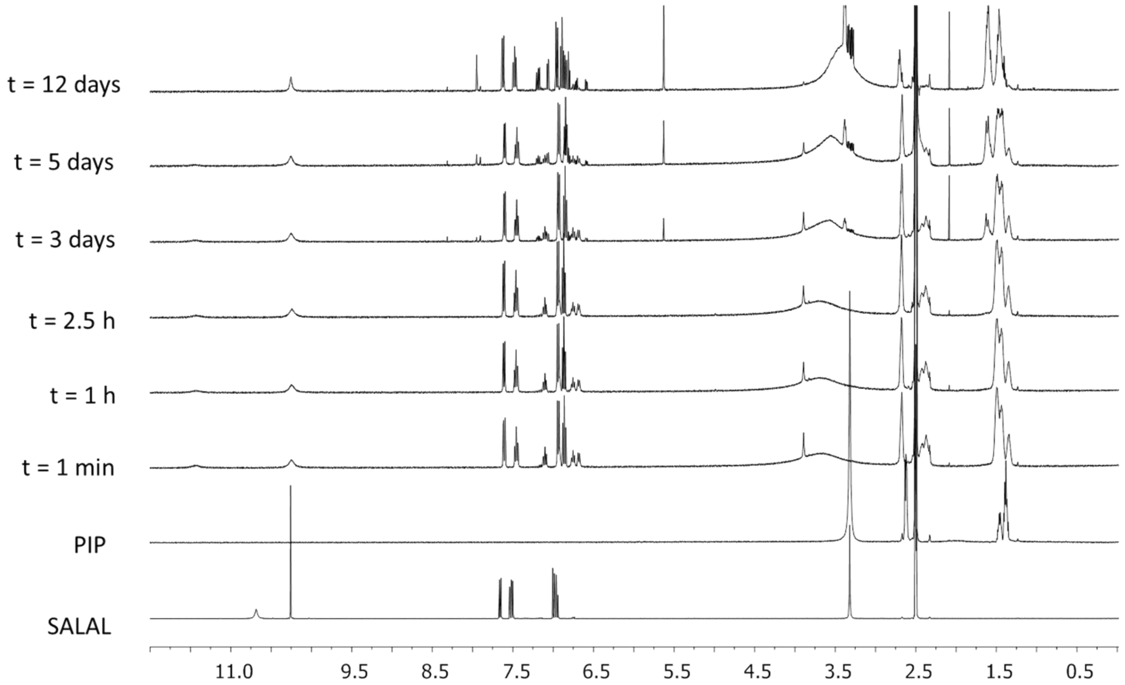

*Figure 2 ^{1}H NMR spectra of the single reactants (**SALAL** and **PIP**) and the reaction mixture at increasing times. Characteristic signals: aldehyde CHO 10.2 ppm, aminal $CH(N\text{-})_2$ 3.9 ppm. Piperidine $N(CH_2)_2$ protons shift only slightly upon formation of the aminal from 2.6 to 2.7 ppm.*

The reaction between **CAXAL** and **IPA** was also monitored by ^{1}H NMR (Figure 3). It is important to note that **CAXAL** is present in the cyclic lactone form in d_6-DMSO solution (Figure 3), and therefore it is not possible to use the disappearance of the characteristic aldehyde signal in order to monitor the reaction progress, but the singlet corresponding to the lactone CH (at 6.67 ppm) is available for this purpose. When **CAXAL** was mixed with 1 eq. of **IPA**, equilibrium was reached almost instantaneously. The aliphatic signals of **IPA** shifted downfield (by up to 0.5 ppm) and the aromatic protons appeared as a clear sharp pair of doublets and a pair of triplets assigned to a single species. However the azomethine CH proton appeared as a broad signal at 6.9 ppm. The HSQC spectrum did not show any cross-peak of this signal with any carbon peak and also the correlation for the N-CH_2 protons was missing. An extended acquisition of the ^{13}C spectrum revealed two broad signals at 46 and 144 ppm, assigned to the N-CH_2 and the azomethine carbons, respectively. The shift of the azomethine carbon signal lies in between the imine (typically around 155 ppm) and the aminal or lactone signal ranges (around 85-95 ppm). From these results it can be concluded that the product formed undergoes rapid interconversion between imine and lactone species.

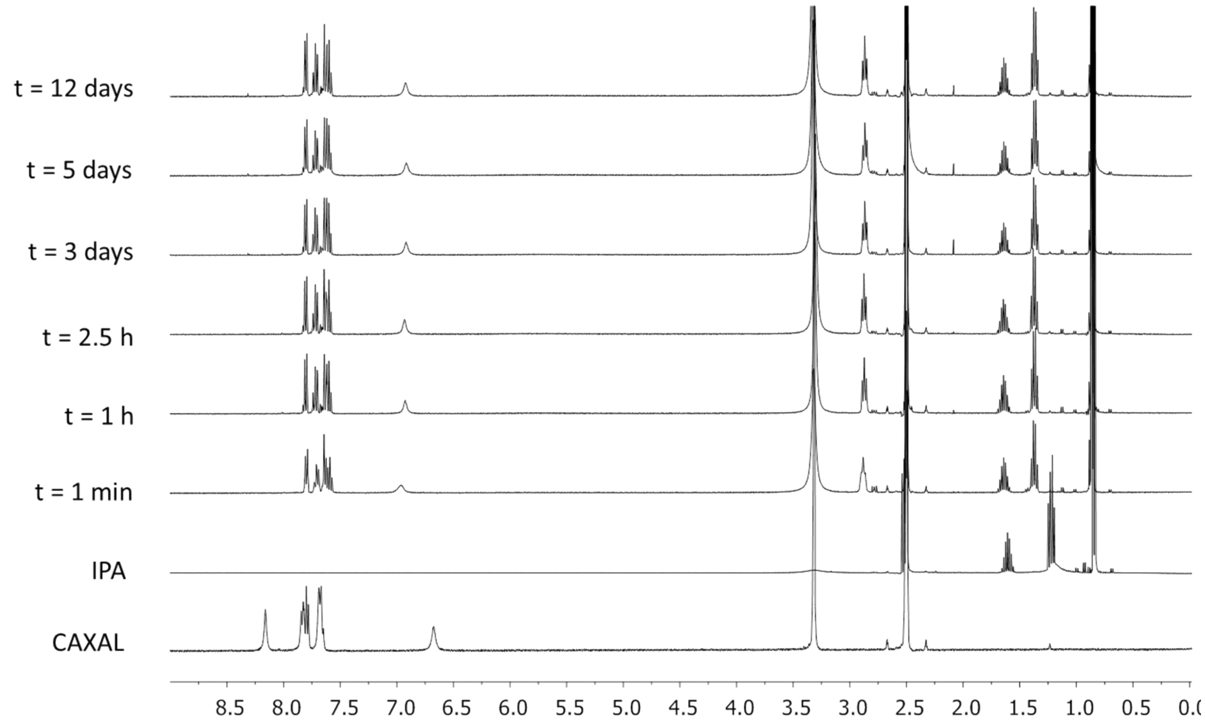

*Figure 3 ^{1}H NMR spectra of the single reactants (**CAXAL** and **IPA**) and the reaction mixture at increasing times (15 mM concentration). Characteristic signals: the lactone form of CAXAL 6.7 ppm, the aminolactone 6.9 ppm (in dynamic exchange with imine form, see the text), aminolactone N-CH_2 2.9 ppm, amine N-CH_2 2.5 ppm (overlap with solvent signal).*

The reaction between **CAXAL** and **PIP** was also monitored by ^{1}H NMR spectroscopy (Figure 4). Although the presence of some intermediate can be observed just after addition (see spectra taken after 1 min: mixture of various species, as observed in the aromatic region and, in particular, by triplet at 2.95 ppm), a unique product is observed within one hour of reaction, which corresponds to the cyclic aminolactone form, as confirmed also by 2D-NMR.

Altogether, **SALAL** showed high reactivity with primary amine **IPA**, but very limited reactivity with secondary amine **PIP** accompanied with an undesired degradation of products. **CAXAL** on the other hand showed ambiguous reactivity for both primary and secondary amine, and in the case of **IPA** the NMR data revealed rapid interconversion between imine and aminolactone species.

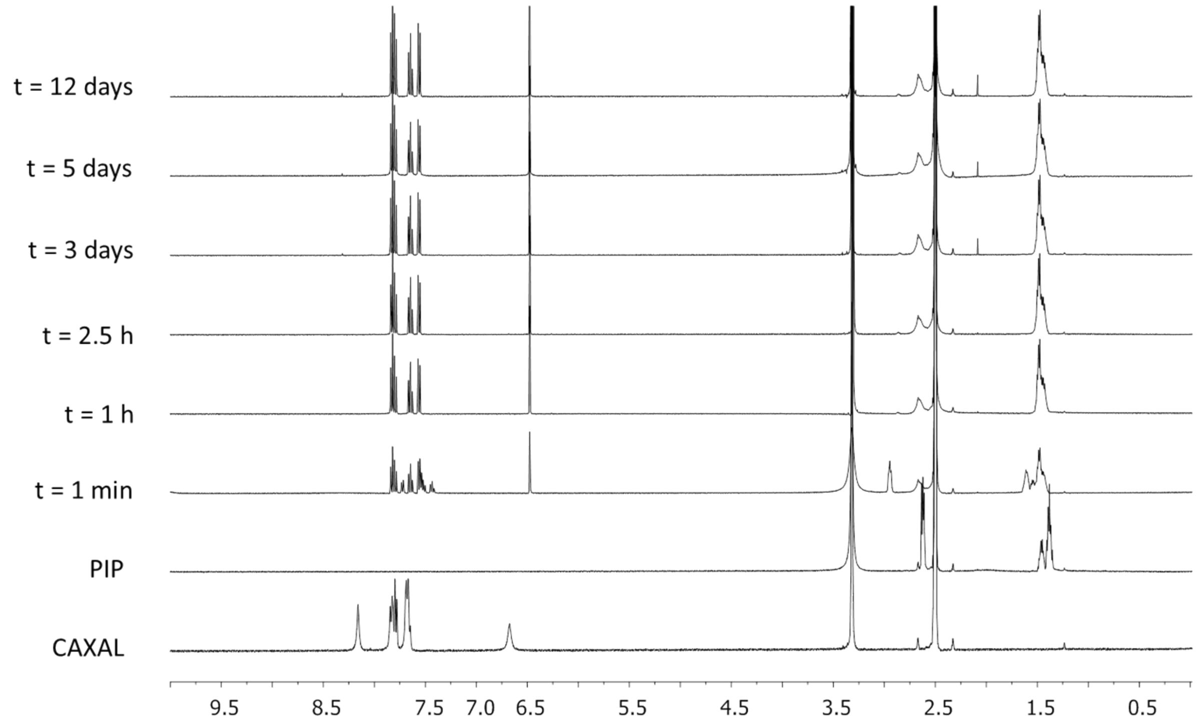

*Figure 4 ^{1}H NMR spectra of the single reactants (**CAXAL** and **PIP**) and the reaction mixture at increasing times. Characteristic signals: the lactone form of CAXAL 6.7 ppm, aminolactone 6.5 ppm.*

3.2 The [1x2] and [2x1] aldehyde-amine mixtures

Reactions containing mixtures of two aldehydes and a single amine [2+1], as well as those containing mixtures of a single aldehyde and two amines [1+2] were performed in order to compare with the [1+1] aldehyde-amine individual mixtures (shown in the previous section).

Reaction between **IPA** and a mixture of **SALAL** and **CAXAL** was monitored by ^{1}H NMR (Figure 5). The spectra corresponding to the mixture of both reaction products (the same as those formed in the individual reactions) is observed immediately after mixing and the reaction product of **CAXAL** (dynamic imine-lactone) the major component at initial stages of the reaction. However, at longer reaction times, the reaction product with **SALAL** (imine) proves to take over and become the major product (77 %), whereas the dynamic imine-lactone product of **CAXAL** is present in only 23 %.

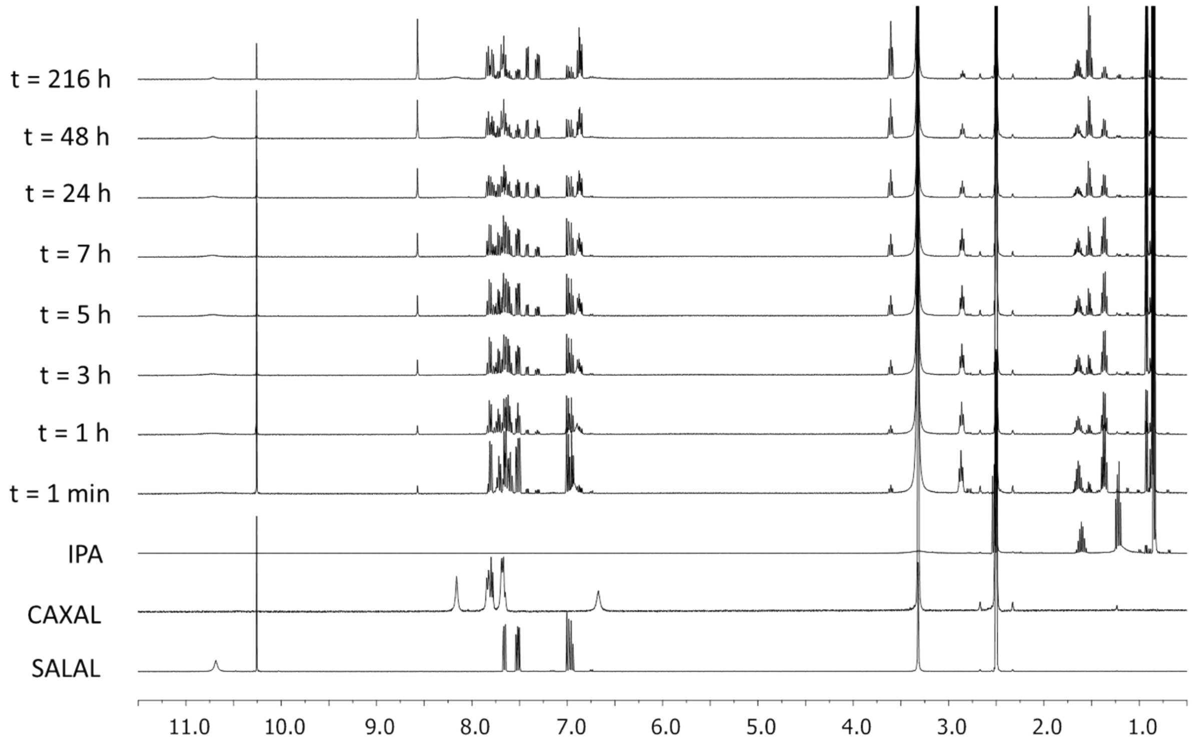

*Figure 5 ^{1}H NMR spectra of the single reactants (**SALAL**, **CAXAL** and **IPA**) and the reaction mixture at increasing times. Characteristic peaks are described in Figure 1 to Figure 4.*

The reaction between **PIP** and a mixture of **SALAL** and **CAXAL** monitored by ^{1}H NMR (Figure 6) shows full selectivity, giving only the lactone as the product of the reaction of **CAXAL** with **PIP** and leaving **SALAL** completely unreacted in the solution.

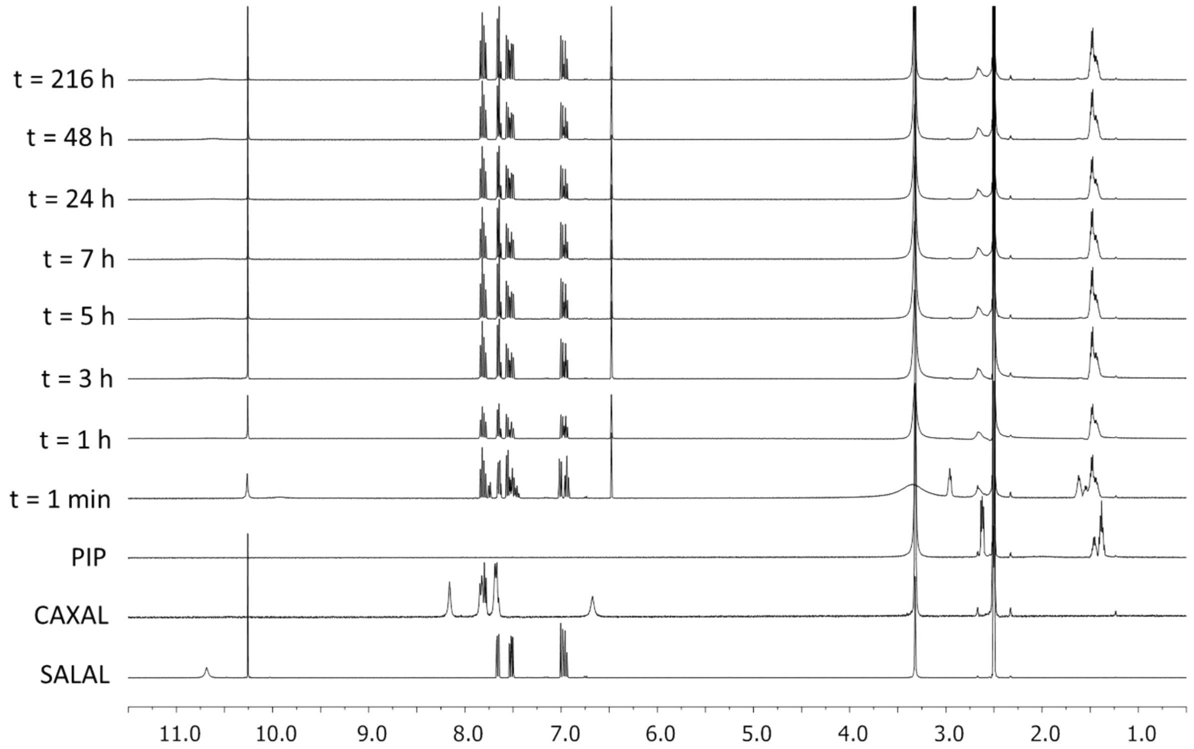

*Figure 6 ^{1}H NMR spectra of the single reactants (**SALAL**, **CAXAL** and **PIP**) and the reaction mixture at increasing times. Characteristic peaks are described in Figure 1 to Figure 4.*

In subsequent experiments, the propensity towards the formation of the expected product was tested in presence of competing amine of a different type, i.e. each aldehyde was reacted with equimolar mixture of the two amines. **SALAL** showed a very high preference for imine formation reacting solely with **IPA** and leaving **PIP** unreacted in the solution. However, the conversion of the aldehyde in this case was not complete and approximately 10 % of the aldehyde remained unreacted although the aldehyde CHO signal was broad.

In contrast, **CAXAL** under the same reaction conditions provided two products: the lactone on the **PIP** ring was formed in about 39 % yield and the imine derived from **IPA** was present in about 61 % yield. Furthermore, the imine of **IPA** exhibited broad signals in the ^{1}H-NMR spectrum, indicating an underlying dynamic process fast on the NMR time scale, a similar process to the case of **CAXAL** reacted only with **IPA**. This was also supported by VT-NMR measurements: by increasing the temperature from 25 to 45 or 65 °C the imine N=CH- signal at 8.52 ppm became sharper and shifted upfield towards the very broad signal at around 6.5 ppm.

3.3 The [2x2] aldehyde-amine mixtures

The previously studied reactants **SALAL**, **CAXAL**, **IPA** and **PIP** were mixed to form a complete four-component [2+2] aldehyde-amine library. The reactions reaches equilibrium within one hour and at the final stage the solution contains only two species: the imine formed from **SALAL** reacted with **IPA** and the aminolactone derived from **CAXAL** and **PIP** (Figure 7). Extended monitoring of the mixture to up to 9 days does not show any development of the NMR spectrum. This experiment shows that the ambiguous reactivity of **CAXAL** is overruled by the selectivity of **SALAL** jeopardizing all the **IPA** from the mixture and thus casting selectivity on **CAXAL**.

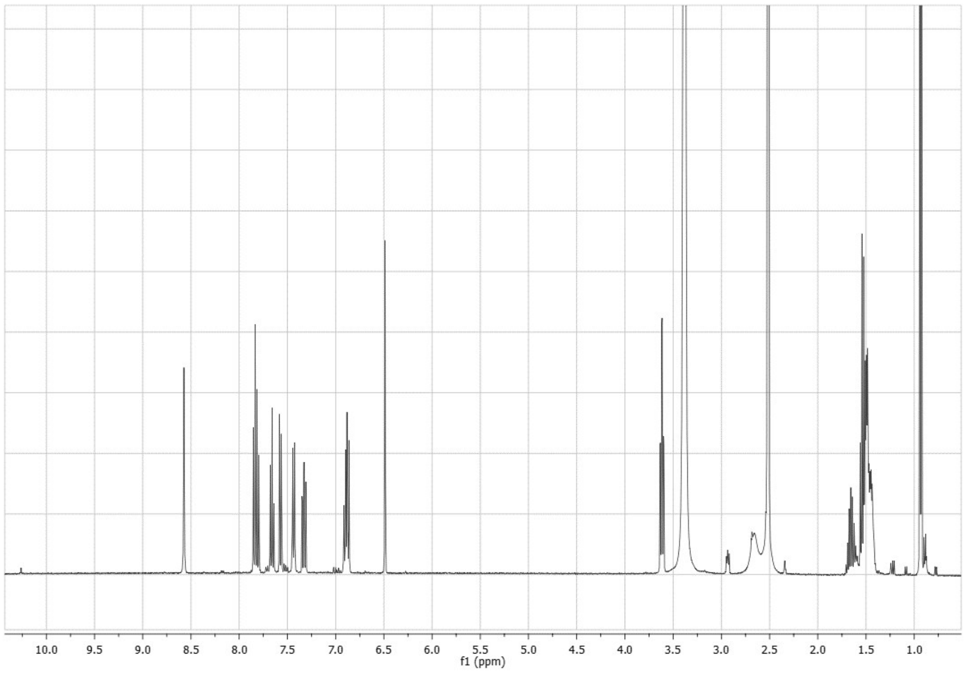

*Figure 7 ^{1}H NMR spectrum of the dynamic library composed of **SALAL** and **CAXAL** treated with **IPA** and **PIP** (20 mM each). While **SALAL** provides only its imine with **IPA** (azomethine singlet at 8.55 ppm, =N-CH_2-triplet at 3.6 ppm), **CAXAL** gives only the lactone with **PIP** (lactone singlet at 6.5 ppm, N-CH_2 signals at 2.6 ppm).*

Taking another aldehyde, **PYRAL**, into play gives the opportunity of formation of aminal species, different to the lactone and imine. Indeed, **PYRAL** efficiently forms both five- and six-membered-ring aminals with *N,N'*-dimethyl-1,2-diaminoethane (**Me_2EDA**) or N,N'-dimethyl-1,3-diaminopropane (**Me_2PDA**), reaching quantitative conversion when mixed with either of the two diamines. When the aldehyde was reacted with an equimolar mixture of **Me_2EDA** and **Me_2PDA**, the preferential formation of the five-membered ring was observed (77 % conversion to the aminal of **Me_2EDA**). Notably, both **SALAL** and **CAXAL** reacted with these diamines as well in high conversion (about 90 % for **SALAL** and quantitative for **CAXAL**), giving similar aminal structures. In the case of **CAXAL** these products were shown (2D NOESY, Figure 8) to undergo dynamic intramolecular exchange between the aminal species and the lactone formed on one of the nitrogen atoms of the corresponding diamine, a process which has been described earlier.[6] On the other hand, **PYRAL** provided its corresponding imine quantitatively when reacted with **IPA**, but gives a complicated mixture of products when mixed with **PIP**, with an overall conversion of the aldehyde of about 40 %.

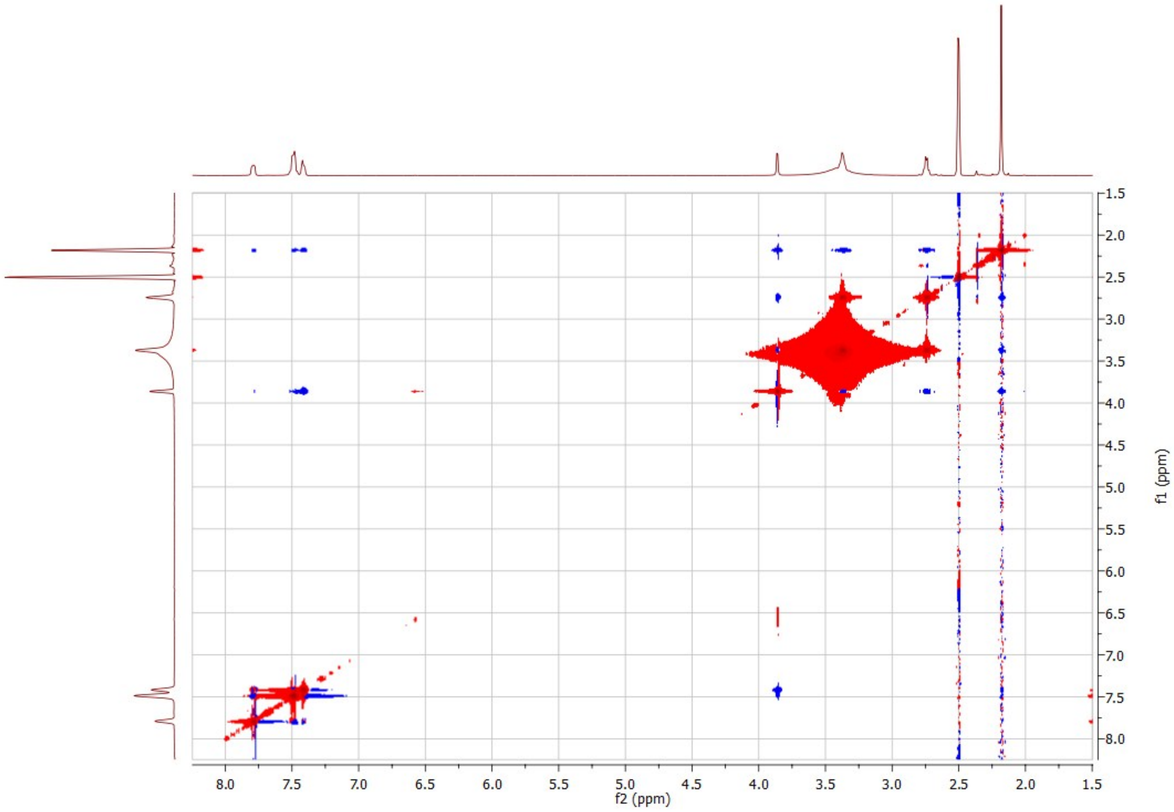

*Figure 8. 2D NOESY spectrum of **CAXAL** reacted with **Me_2EDA** (20 mM each, d_6-DMSO + 1 % D_2O). Chemical exchange cross-peaks are visible in the aliphatic region between the CH_2 groups of the diamine indicating an end-to-end displacement process. Second pair of chemical exchange cross-peaks is observable between the aminal signal at 3.7 ppm and the very weak lactone signal at around 6.6 ppm. This clearly demonstrates that the **CAXAL** moiety is fluxionally displacing between the nitrogen atoms of **Me_2EDA**.*

The combination of **PYRAL** and **SALAL** was examined next. The propensity of **PYRAL** to form the aminal (both with **Me_2EDA** and **Me_2PDA**) was assessed in the presence of the imine-forming primary amine **IPA**. To this end, **PYRAL** was mixed with **IPA** and one of the diamines and the NMR spectra were followed in time. The spectra taken shortly after mixing showed the presence of the imine as the major product of the reaction. However, the mixture continued to evolve over time, to finally reach equilibrium after 3 days in which the aminal was the dominant species in case of both diamines (79 % with **Me_2PDA** and 82 % with **Me_2EDA**, the difference to 100 % is the imine of **IPA**). It is noteworthy that the high preference for formation of the five-membered aminal in the separate experiment (77:23 in favour of the aminal of **Me_2EDA** over **Me_2PDA**) is translated in only 3 % enhancement of the aminal-over-imine selectivity in the competition experiment with **IPA** (Figure 9).

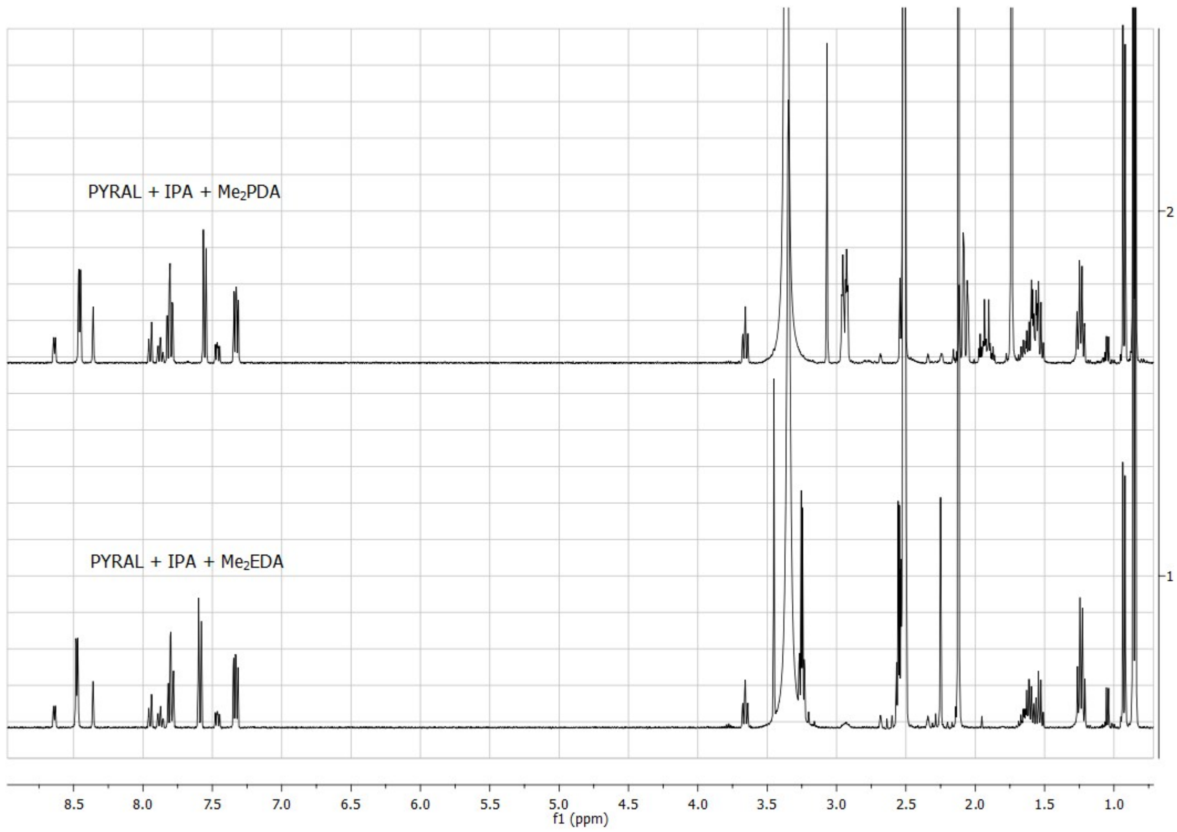

*Figure 9. ^{1}H-NMR spectra of **PYRAL** reacted with **IPA** and one of the studied diamines. In both cases, the aminal is the dominant species in the equilibrium as indicated by the characteristic singlet at 3.5 ppm for **Me_2EDA** and 3.1 for **Me_2PDA**, accompanied by small amount of the imine formed by the reaction with **IPA** (singlet at 8.4 ppm).*

When **SALAL** was exposed to the same amine mixtures, i.e. 1 eq. of **IPA** with 1 eq. of one of the diamines, the imine was formed first, as in the reaction with **PYRAL**, but the reaction evolved over three days to finally reach an equilibrium containing mostly the aminal (about 60 % for both diamines). These experiments show that both **PYRAL** and **SALAL** have a high propensity to form aminals, regardless of whether the size of the ring is five- or six-membered. For comparison, the two aldehydes were allowed to compete for one amine. Thus when the mixture of **SALAL** and **PYRAL** was mixed with 1 eq. of **IPA**, the conversion to corresponding imines (determined by ^{1}H NMR as the ratio of the aldehyde and imine signal intensity for each corresponding aldehyde) was 63 % and 27 % for **SALAL** and **PYRAL** respectively. Similarly, when these aldehydes were reacted with 1 eq. of **Me_2PDA**, the conversion to aminals (determined in the same way as for imines) reached 70 % and 30 % for **SALAL** and **PYRAL** respectively (Figure 10). These results show that the reactivity of **SALAL** is overruling the reactivity of **PYRAL** (under given conditions) regardless whether the product formed is either imine or aminal.

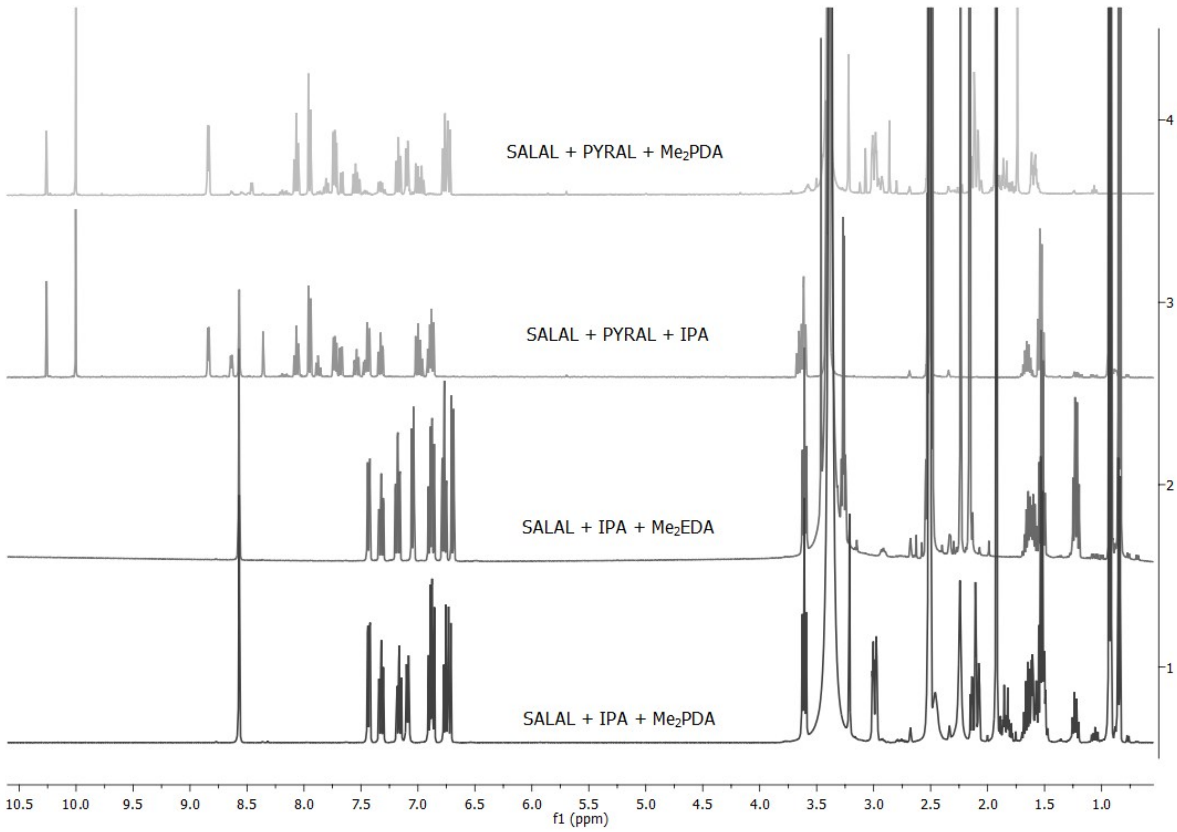

*Figure 10. ¹H-NMR spectra of the [2+1] and [1+2] libraries containing **SALAL**, **PYRAL**, **IPA** and **Me_2PDA** or **Me_2EDA**. Characteristic imine singlet of the imine of **SALAL** appears at around 8.5 ppm and for the imine of **PYRAL** around 8.3 ppm. Aldehyde signals follow the same trend in chemical shift, appearing at around 10.3 and 10.0 for **SALAL** and **PYRAL** respectively.*

The full [2x2] library of the **SALAL** and **PYRAL** combination was then examined. An equimolar mixture of the two aldehydes was reacted with of equimolar mixture of **IPA** and **Me_2EDA**, or **IPA** and **Me_2PDA** (20 mM of each reactant, d_6-DMSO + 1 % D_2O). In both cases the results were similar: rapid high conversion of **SALAL** to its products was observed and the ratio between aminal and imine changed slightly in favour of the imine as the reaction approached equilibrium. In contrast, **PYRAL** remained largely unreacted over the first few hours. As the reaction evolved, the aminal of **PYRAL** was formed in larger quantities while the concentration of corresponding imine stayed relatively constant. Equilibrium was reached within 24 hours and the final composition for **Me_2PDA** amounted to 76 % imine of **SALAL** and 24% of the aminal and exactly the opposite ratio for the imine and the aminal of **PYRAL**, respectively (Figure 11). In the case of **Me_2EDA**, the relative concentrations of the imine-aminal species reached the ratio of 80:20 and followed the same trend as in the case of **Me_2PDA**.

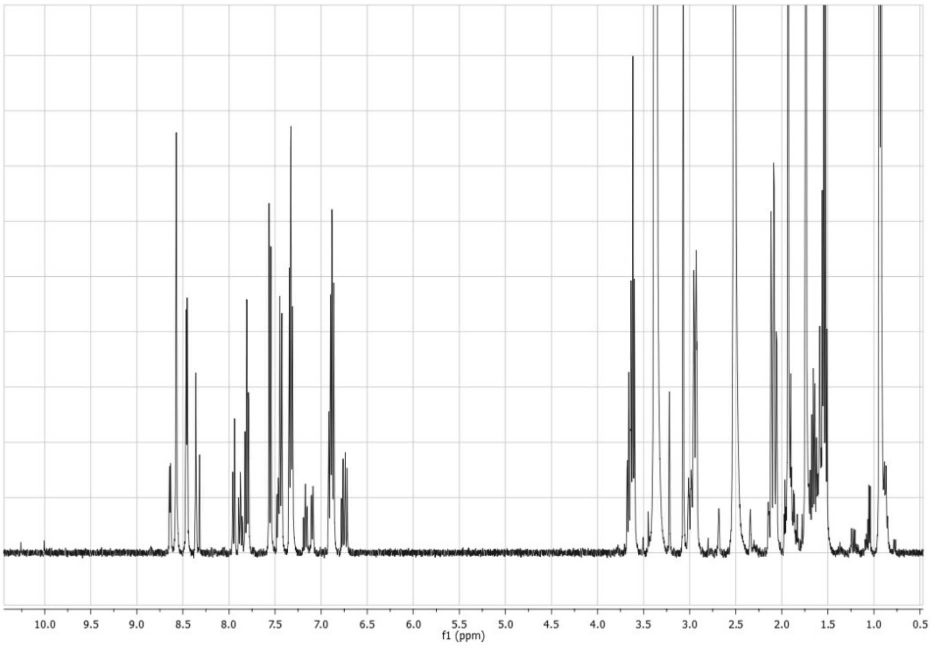

*Figure 11 ^{1}H-NMR spectrum of the [2+2] library consisting of **SALAL**, **PYRAL**, **IPA** and **Me$_2$PDA**. Only trace amounts of unreacted aldehydes are observed (bellow 1 %). The preferred products (imine of **SALAL** and aminal of **PYRAL**) are formed in 76 % conversion while the non-preferred ones are present in 24 %. The imine of SALAL gives signal at 8.6 ppm, the imine of PYRAL at 8.4 ppm, aminal of PYRAL at 3.0 ppm and SALAL aminal at 3.2 ppm.*

Finally, the combination of **PYRAL** and **CAXAL** with their matching amines was investigated. As indicated above, **CAXAL** reacted with both aminal-forming diamines in quantitative conversion, while **PYRAL** with **PIP** gave a complicated mixture of products in overall 40 % conversion. When **PYRAL** was mixed with 1 equivalent of each **PIP** and **Me$_2$PDA**, the corresponding aminal formed with a high selectivity of about 88 % and 12 % was converted to the aminal formed by the reaction with two molecules of **PIP**. However, **CAXAL** reacted with the same amine mixture gives a broad and complicated spectrum. From 2D NMR analysis the products were assigned as the lactone formed on **PIP** and dynamic aminal-lactone of **Me$_2$PDA**. The shape of the signals allowed only a rough estimation of the relative ratio of the products, indicating that 10 % of **CAXAL** had reacted with **PIP** and about 90 % with **Me$_2$PDA** (Figure 12). The combination of the two aldehydes with one amine was also examined: **PIP** reacted with the two aldehydes to form solely the lactone of **CAXAL**, leaving **PYRAL** completely unreacted in solution, while **Me$_2$PDA** gave a mixture of the two aminals in 44 % and 55 % conversion for **PYRAL** and **CAXAL** respectively (Figure 12).

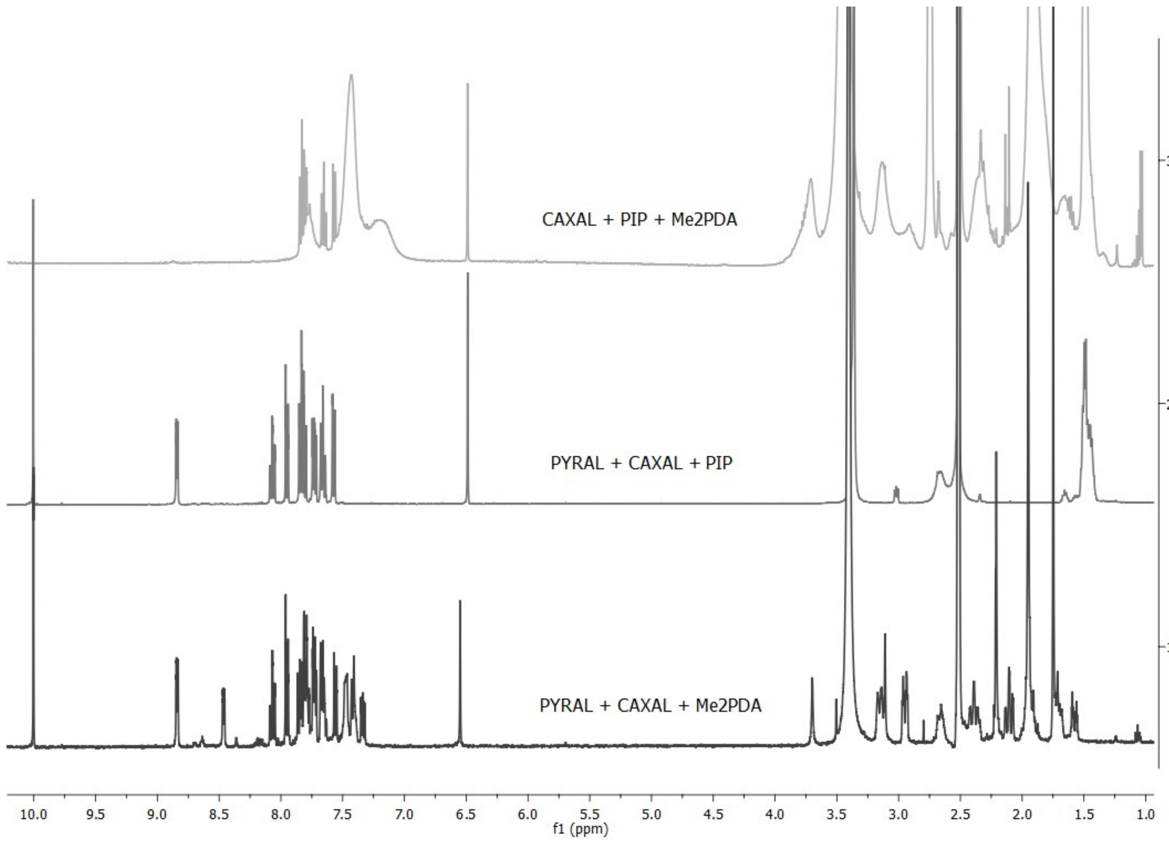

*Figure 12. ^{1}H-NMR spectra of the [2+1] and [1+2] libraries containing **PYRAL**, **CAXAL**, **PIP** and **Me_2PDA**. The hydroxy-lactone (closed of form of the aldehyde of **CAXAL**) peak at 6.55 ppm shifts to 6.45 ppm upon reaction with **PIP** and formation of the corresponding aminolactone. When reacted with the mixture of **PIP** and **Me_2PDA**, **CAXAL** provides very broad signals in the NMR spectrum showing a dynamic exchange between aminal and lactone.*

As previously, the [2x2] aldehyde-amine selection experiment was performed for **PYRAL**, **CAXAL**, **Me_2PDA** and **PIP**. When all four reagents were mixed, an immediate reaction of **CAXAL** was observed, giving both the lactone of **PIP** and the aminal with **Me_2PDA**. As the reaction proceeded, the ratio increased in favour of the lactone due to the slower formation of the aminal of **PYRAL**, reaching equilibrium after 3 days. The equilibrated mixture was composed of 97 % the aminal of **PYRAL** and only 3 % of the aldehyde remained unreacted, while **CAXAL** formed the lactone in 94 % conversion and the aminal in only < 5 % (signal overlapped with residual water signal). Notably, replacement of the diamine by **Me_2EDA** led to almost identical results within ± 2 % difference in relative conversions (Figure 13).

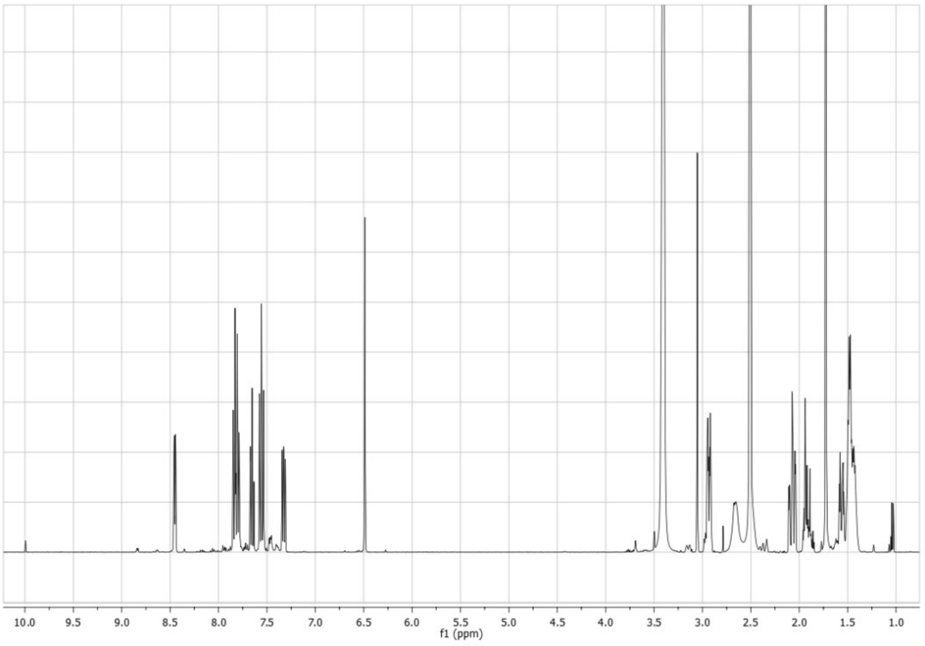

*Figure 13 ^{1}H-NMR spectrum of the mixture of **PYRAL** and **CAXAL** reacted with **Me$_2$PDA** and **PIP**. The quantification was based on the azomethine signals. The lactone of **CAXAL** appears as sharp singlet at 6.5 ppm, while the minor aminal product gives a small broad signal around 3.7 ppm. Similarly, the aminal of **PYRAL** shows a sharp singlet at 3.0 ppm and the unreacted aldehyde is observed as small peak at 10 ppm.*

The kinetics of the imine formation described in the maintext was performed in d_6-DMSO buffered with 0.8 M triethanolaminal - HCl mixture. Reactants were mixed in equimolar ratios at 20 mM concentration level and followed by NMR at controlled temperature of 25 °C. The shown conversion was calculated from the characteristic signals of starting materials and products (see spectra above).

3.4 Selection matrices for the full [3x3] library

	SALAL	PYRAL	CAXAL
IPA	0.88	0.12	0
Me2PDA	0.12	0.88	0
PIP	0	0	1

	SALAL	PYRAL	CAXAL
[redacted]	[redacted]	[redacted]	[redacted]
Me2PDA	0.21	0.89	0
PIP	0	0	1

	SALAL	PYRAL	[redacted]
IPA	0.83	0.17	[redacted]
Me2PDA	0.17	0.83	[redacted]
PIP	0	0	[redacted]

	SALAL	PYRAL	CAXAL
IPA	0.71	0.29	0
[redacted]	[redacted]	[redacted]	[redacted]
PIP	0	0	1

	SALAL	[redacted]	CAXAL
IPA	0.6	[redacted]	0
Me2PDA	0.4	[redacted]	0.59
PIP	0	[redacted]	0.41

	SALAL	PYRAL	CAXAL
IPA	0.7	0.25	0.07
Me2PDA	0.12	0.52	0.36
[redacted]	[redacted]	[redacted]	[redacted]

	[redacted]	PYRAL	CAXAL
IPA	[redacted]	0.35	0.23
Me2PDA	[redacted]	0.65	0.24
PIP	[redacted]	0	0.53

	SALAL	[redacted]	[redacted]
IPA	0.5	[redacted]	[redacted]
Me2PDA	0.5	[redacted]	[redacted]
PIP	0	[redacted]	[redacted]

	[redacted]	[redacted]	CAXAL
IPA	[redacted]	[redacted]	0.33
Me2PDA	[redacted]	[redacted]	0.63
PIP	[redacted]	[redacted]	0.04

	[redacted]	PYRAL	[redacted]
IPA	[redacted]	0.22	[redacted]
Me2PDA	[redacted]	0.78	[redacted]
PIP	[redacted]	0	[redacted]

	SALAL	[redacted]	CAXAL
IPA	0.81	[redacted]	0.17
Me2PDA	0.19	[redacted]	0.74
[redacted]	[redacted]	[redacted]	[redacted]

	[redacted]	PYRAL	CAXAL
IPA	[redacted]	0.49	0.37
Me2PDA	[redacted]	0.39	0.52
[redacted]	[redacted]	[redacted]	[redacted]

	SALAL	PYRAL	[redacted]
IPA	0.75	0.25	[redacted]
[redacted]	[redacted]	[redacted]	[redacted]
PIP	0	0.3	[redacted]

	SALAL	[redacted]	CAXAL
IPA	1	[redacted]	0
[redacted]	[redacted]	[redacted]	[redacted]
PIP	0	[redacted]	1

	[redacted]	PYRAL	CAXAL
IPA	[redacted]	0.86	0.11
[redacted]	[redacted]	[redacted]	[redacted]
PIP	[redacted]	0	0.89

	SALAL	PYRAL	CAXAL
[redacted]	[redacted]	[redacted]	[redacted]
PIP	0	0	1

	SALAL	PYRAL	CAXAL
IPA	0.58	0.27	0.15
[redacted]	[redacted]	[redacted]	[redacted]

	SALAL	PYRAL	CAXAL
[redacted]	[redacted]	[redacted]	[redacted]
Me2PDA	0.13	0.7	0.18
[redacted]	[redacted]	[redacted]	[redacted]

	SALAL	PYRAL	[redacted]
IPA	0.75	0.25	[redacted]
Me2PDA	0.25	0.75	[redacted]
[redacted]	[redacted]	[redacted]	[redacted]

	[redacted]	PYRAL	CAXAL
[redacted]	[redacted]	[redacted]	[redacted]
Me2PDA	[redacted]	0.97	0.06
PIP	[redacted]	0	0.94

	SALAL	[redacted]	[redacted]
[redacted]	[redacted]	[redacted]	[redacted]
Me2PDA	1	[redacted]	[redacted]
PIP	0	[redacted]	[redacted]

	[redacted]	[redacted]	CAXAL
[redacted]	[redacted]	[redacted]	[redacted]
Me2PDA	[redacted]	[redacted]	0.9
PIP	[redacted]	[redacted]	0.1

	[redacted]	PYRAL	[redacted]
[redacted]	[redacted]	[redacted]	[redacted]
Me2PDA	[redacted]	0.88	[redacted]
PIP	[redacted]	0.12	[redacted]

	SALAL	[redacted]	[redacted]
IPA	0.9	[redacted]	[redacted]
[redacted]	[redacted]	[redacted]	[redacted]
PIP	0.05	[redacted]	[redacted]

	SALAL	PYRAL	
Me2PDA	0.9	0.1	
PIP	0	0.34	

			CAXAL
IPA			0.61
PIP			0.39

	SALAL		CAXAL
Me2PDA	0.75		0.14
PIP	0		0.86

		PYRAL	
IPA		0.74	
PIP		0.18	

	SALAL		
IPA	0.4		
Me2PDA	0.6		

	SALAL		CAXAL
PIP	0		1

			CAXAL
IPA			0.27
Me2PDA			0.73

		PYRAL	CAXAL
PIP		0	1

		PYRAL	
IPA		0.21	
Me2PDA		0.79	

	SALAL	PYRAL	
Me2PDA	0.70	0.30	

	SALAL	PYRAL	
IPA	0.63	0.27	

	SALAL		CAXAL
Me2PDA	0.31		0.69

	SALAL		CAXAL
IPA	0.77		0.23

		PYRAL	CAXAL
Me2PDA		0.44	0.55

		PYRAL	CAXAL
IPA		0.65	0.35

	SALAL		
IPA	1		

	SALAL	PYRAL	[redacted]
[redacted]	[redacted]	[redacted]	[redacted]
[redacted]	[redacted]	[redacted]	[redacted]
PIP	0	0.4	[redacted]

	[redacted]	[redacted]	CAXAL
IPA	[redacted]	[redacted]	1
[redacted]	[redacted]	[redacted]	[redacted]
[redacted]	[redacted]	[redacted]	[redacted]

	[redacted]	PYRAL	[redacted]
IPA	[redacted]	1	[redacted]
[redacted]	[redacted]	[redacted]	[redacted]
[redacted]	[redacted]	[redacted]	[redacted]

	SALAL	[redacted]	[redacted]
[redacted]	[redacted]	[redacted]	[redacted]
Me2PDA	0.92	[redacted]	[redacted]
[redacted]	[redacted]	[redacted]	[redacted]

	SALAL	[redacted]	[redacted]
[redacted]	[redacted]	[redacted]	[redacted]
[redacted]	[redacted]	[redacted]	[redacted]
PIP	0.3	[redacted]	[redacted]

	[redacted]	[redacted]	CAXAL
[redacted]	[redacted]	[redacted]	[redacted]
Me2PDA	[redacted]	[redacted]	1
[redacted]	[redacted]	[redacted]	[redacted]

	[redacted]	[redacted]	CAXAL
[redacted]	[redacted]	[redacted]	[redacted]
[redacted]	[redacted]	[redacted]	[redacted]
PIP	[redacted]	[redacted]	1

	[redacted]	PYRAL	[redacted]
[redacted]	[redacted]	[redacted]	[redacted]
Me2PDA	[redacted]	1	[redacted]
[redacted]	[redacted]	[redacted]	[redacted]

	[redacted]	PYRAL	[redacted]
[redacted]	[redacted]	[redacted]	[redacted]
[redacted]	[redacted]	[redacted]	[redacted]
PIP	[redacted]	0.4	[redacted]

selector

0.123	-0.220	-0.191
-0.232	0.145	-0.337
-0.022	-0.097	0.166

mean

0.732	0.419	0.259
0.461	0.688	0.489
0.023	0.116	0.744

std

0.169	0.265	0.264
0.318	0.243	0.310
0.078	0.163	0.351

3.5 Multivalency experiments

Initially, the aldehydes **CAXAL** and **SALAL** were reacted separately with the diamine. In both cases broad NMR spectra were recorded, indicating fast exchange between species, and in the case of **CAXAL** the azomethine peak disappeared completely from the spectrum. Next, a mixture of the aldehydes (20 mM each) was treated with **BnEDA** (1 eq.) and allowed to equilibrate at 60 °C overnight. The dominant product formed (about 70 %) was the expected However, **CAXAL** also provided its imine (15 %) and about 17 % remained unreacted (equilibrium between aldehyde and hydroxy-lactone). The formation of the imine of **CAXAL** also resulted in larger amounts of unreacted **SALAL** (30 %) as it cannot react with secondary amines (Figure 14).

The experiment was repeated using identical conditions for various polyamines and the results are discussed in the main text including the final conversion to the desired products. The following Figures provide the corresponding NMR traces of these experiments: a) symmetric **en_2N_3** and **pr_2N_3**

reacted with **SALAL**, **PYRAL** (Figure 15), b) unsymmetrical triamines **en-prN$_3$** and spermidine reacted **SALAL** and **PYRAL** (Figure 16), c) tetramine **en$_3$N$_4$** reacted **SALAL** and **PYRAL** (Figure 17). NMR signal assignments are based on the spectra of individual two-component mixtures as shown above (Figure 1 – Figure 13). Only the major products formed are assigned in the spectra. However, other species are formed in various amounts as well. Due to the presence of large number of species at low concentration in the solution and signal overlap, complete signal assignment and quantification of these products is not possible. In general, the difference between the indicated conversion and 100 % is the sum of all other possible products, comprising for unreacted aldehydes, mono-imines, (hemi)aminals, lactones and undesired regioisomers.

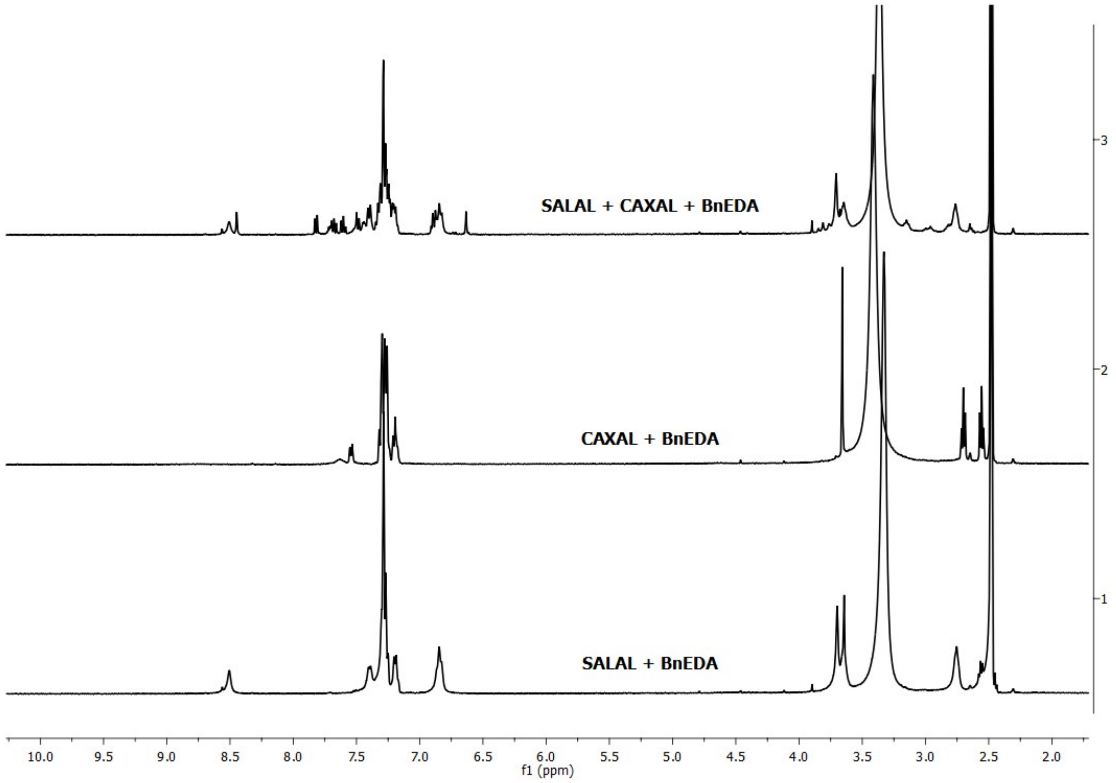

*Figure 14. ^{1}H-NMR spectra of **BnEDA** reacted with **SALAL** (bottom trace), **CAXAL** (middle trace) and the mixture of **SALAL** and **CAXAL** (top trace). Dynamic imine-aminal(-lactone) exchange results in broad signals in the NMR spectrum, unless both nitrogen atoms are occupied by the preferred aldehyde derivative – **SALAL** on the primary amine and **CAXAL** on the secondary one. Characteristic signals: SALAL monoamine in dynamic exchange 8.5 ppm, SALAL imine 8.4 ppm with CAXAL lactone 6.5 ppm. The CAXAL azomethine proton in the reaction with BnEDA is not visible due to the rapid exchange on the NMR time scale.*

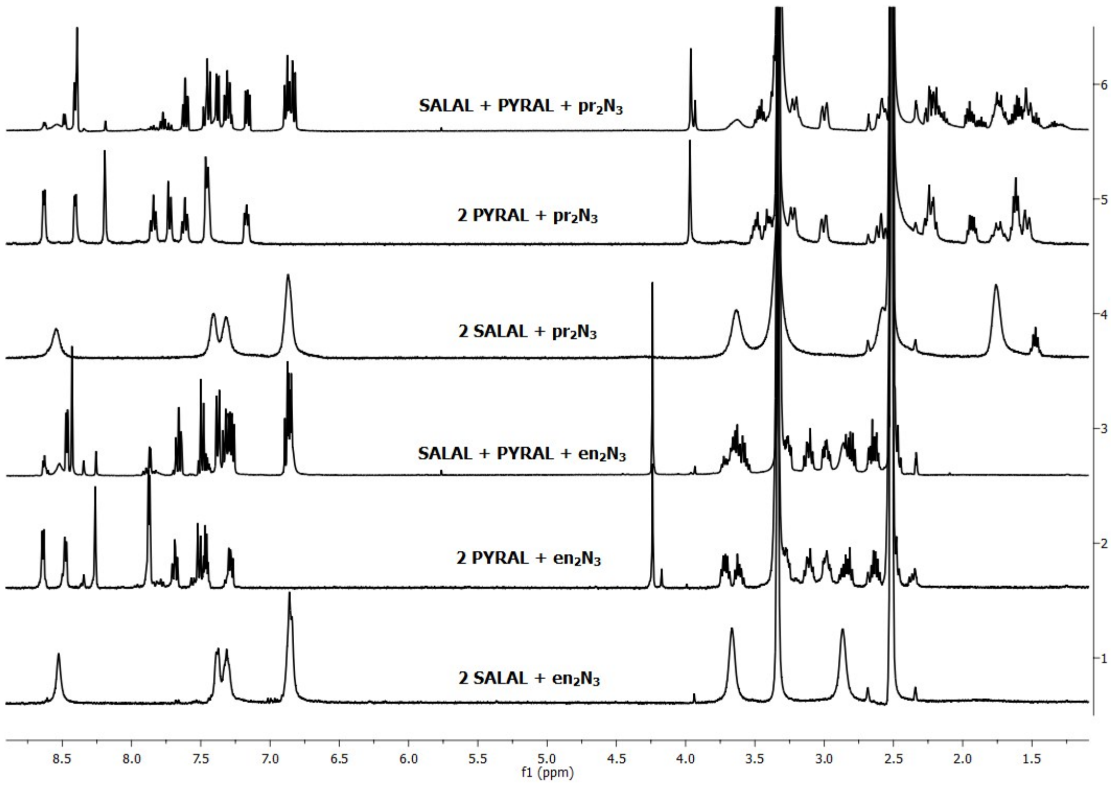

*Figure 15. ^{1}H-NMR spectra of symmetric triamines **en_2N_3** and **pr_2N_3** reacted with **SALAL**, **PYRAL** and the mixture of the two. Characteristic signals: SALAL imine 8.5 ppm, PYRAL imine 8.2 ppm (pr_2N_3) or 8.3 ppm (en_2N_3), PYRAL aminal 4.2 ppm (en_2N_3) or 4.0 ppm (pr_2N_3).*

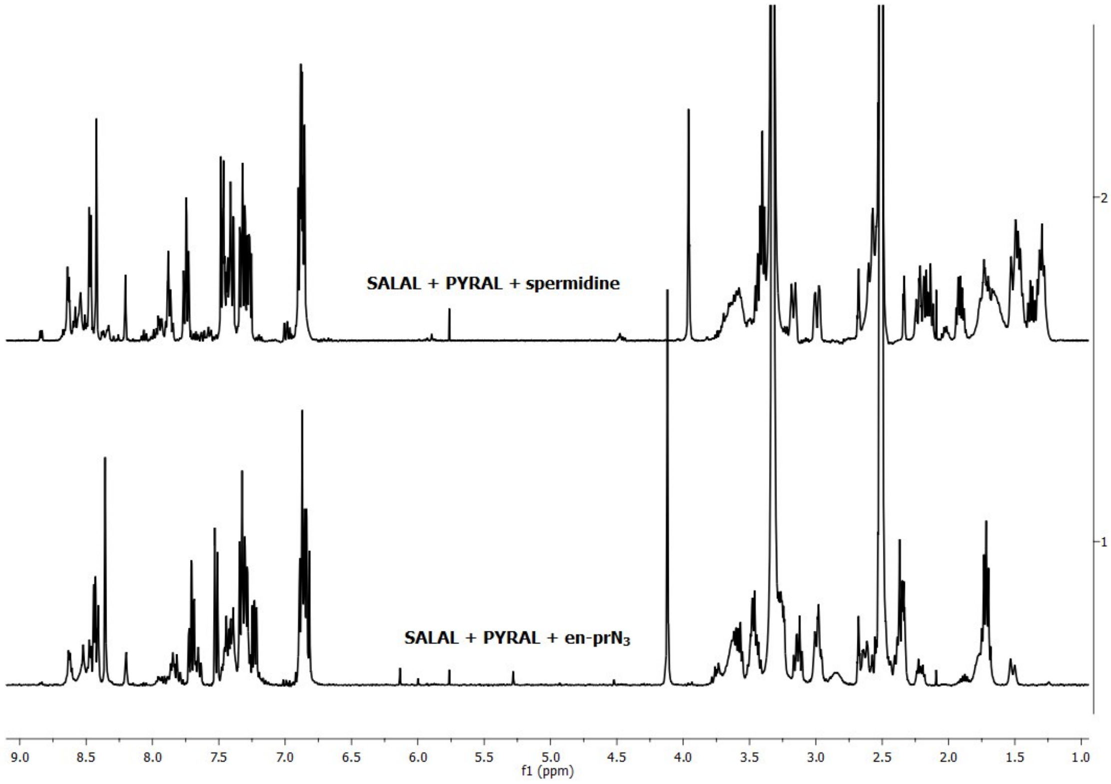

*Figure 16. ^{1}H-NMR spectra of unsymmetrical triamines **en-prN_3** and spermidine reacted with equimolar mixture of **SALAL** and **PYRAL**. Characteristic signals: azomethine signal of the five-membered aminal of PYRAL 4.1 ppm, SALAL*

imine 8.4 ppm, PYRAL imine 8.2 ppm. Trace amounts of six-membered aminal of PYRAL are probably formed as well, but some signals overlap.

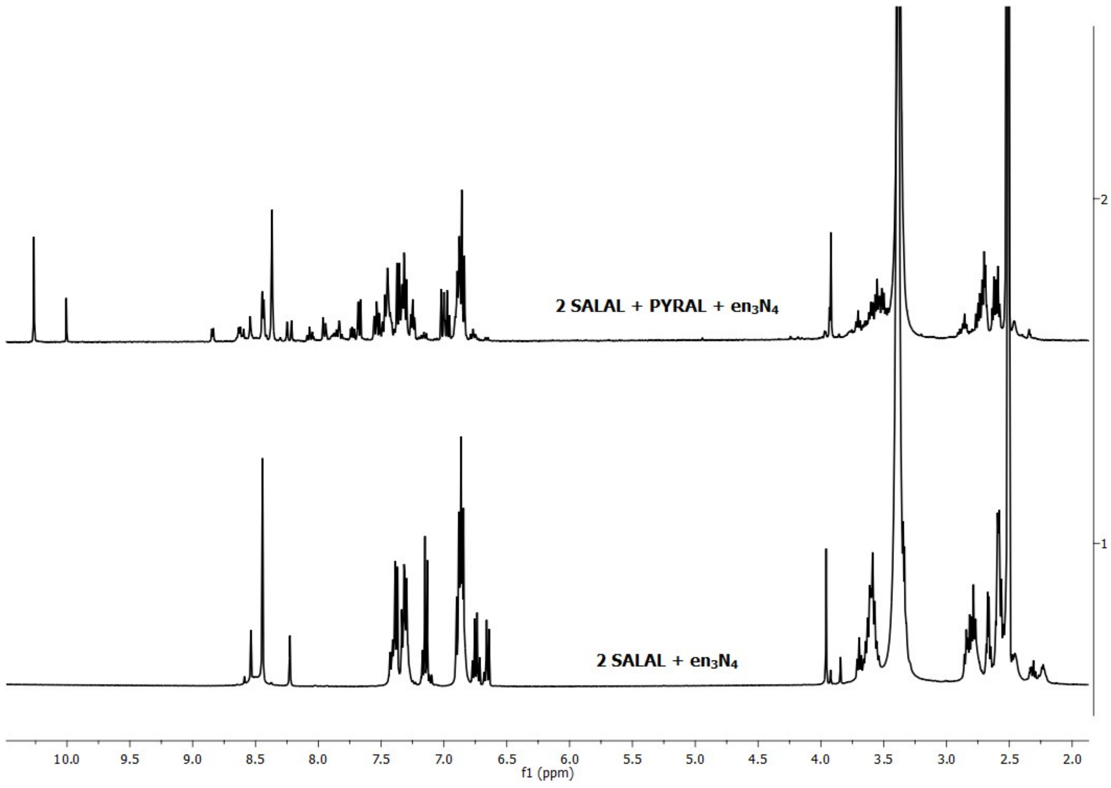

*Figure 17. ^{1}H-NMR spectra of **en_3N_4** reacted first with 2 eq. of **SALAL** (bottom trace) and after addition of 1 eq. of **PYRAL** (top trace). Higher amounts of free aldehydes leads to relatively low overall conversion to the desired bis-imine-aminal of about 60 %. Characteristic signals: SALAL bis-imine on both termini 8.4 ppm, SALAL mono-imines and mixed imine-aminal 8.5, 8.2 and 4.0 ppm, PYRAL aminal 3.9 ppm.*

4. Dynamic selective protecting groups

4.1 Optimization of reaction conditions – general tendencies and results

The reaction was also compared in two different solvents, acetonitrile and ethanol, finding no difference in isolated yields, which allows for adjustment of the medium depending on the solubility issues which might come up for some substrates. There are also several possibilities for the choice of the deprotecting agent. Successful imine exchange is achieved if a more stable imine derivative is formed. In the present case we have explored benzhydrazide and O-benzylhydroxylamine, giving after deprotection benzhydrazone and *O*-benzyloxime, respectively. These two byproducts of the deprotection step differ largely in their polarity, which was found beneficial during chromatographic purification of the products, as better separation can be achieved by the choice of different deprotecting agent. Moreover, large number of other hydrazide and hydroxylamines are commercially available and thus the polarity of the deprotection byproduct can be varied over a wide range.

To gain comprehensive insight into the evolution of the reaction, small samples were taken from the reaction mixtures after each step performed and analysed by NMR and LC-MS. Characteristic signals in the NMR spectra (aldehyde, imine, aminal) enable direct observation of ongoing transformation.

LC-MS analysis was used to confirm or disprove the formation of products. Reported yields are always given in terms of isolated compounds.

4.1.1 Bis-reaction with SALAL

4.1.1.1 Electrophiles tested

1st Electrophile	2nd Electrophile	Outcome (best yield)
Ph_2NCOCl	BzCl	75%
Anthracenemethylene chloride	CbZCl	No product found
CbzCl	PhNCO	71%
PhNCO	CbzCl	82%
BocAlaOSu	BocPheOSu	32%
Ac_2O	CbzGlyONp	37%
BocLeuOSu	p-NO_2BzCl	No product found
CbzGlyONp	BocAlaOSu	82%
CbzGlyONp	BocLeuOSu	83%
Acrylonitrile	Ac_2O	No product found
Acrylonitrile	BzCl	No product found
Ac_2O	PhNCO	No product found
PhNCO	Ac_2O	No product found

Different electrophiles were tested on the model system *N*-methylaminopropylamine. The reaction of diphenylcarbamoyl chloride and benzoyl chloride was examined best. The highest yield after optimization was 75% of isolated bis-substituted product. The reactions of benzyl chloroformate and phenyl isocyanate, phenyl isocyanate and benzyl chloroformate (inverted order), CbzGlyONp and BocAlaOSu as well as CbzGlyONp and BocLeuOSu gave yields in the same range (71-83%) after several optimizations. The reactions of BocAlaOSu and BocPheOSu as well as acetic anhydride and CbzGlyONp did not work in the same way. Only low yields below 40% were achieved. This is probably due to the lower reactivity of the succinimide activated alanine, which is not reactive enough in the first step. With acetic anhydride, the reactions usually gave poor results (see below), which might also be due to a low reactivity or side reactions. No product could be identified in the following reactions: anthracene methylenechloride and benzyl chloroformate, BocleuOSu and *para*-nitrobenzoyl chloride, acrylonitrile and acetic anhydride, acrylonitrile and benzoyl chloride, acetic anhydride and phenyl isocyanate as well as with phenyl isocyanate and acetic anhydride (inverted order). The antracene methylenechloride was probably not reactive enough and sterically hindered. The same might be the case for BocLeuOSu. Acetic anhydride might also be not reactive enough or undergoes side reactions. Acrylonitrile should be reactive enough but might undergo some other reaction than the desired one.

4.1.1.2 Solvents

Acetonitrile or ethanol was used. Both in p.a. quality and not dried or otherwise purified before the reactions. No difference could be observed between those solvents, the yields of isolated products were similar. Ethanol was chosen, if the reagents were polar, so that a higher solubility could be achieved and therefore sometimes a better yield. But the imine formation with SALAL was the same

in both solvents: after 2 h, only 6% of unreacted SALAL was observed by NMR in acetonitrile and 9% in ethanol.

4.1.1.3 Bases

If HCl or another acid could be liberated in the course of a reaction, 1 equivalent of base was added before the acid-liberating species. First, triethylamine was employed, which gave already good yields (around 60%). With the stronger base DBU, the yield could be enhanced (above 70%). The base was important in the addition of the 1st electrophile, because acid liberation led to hydrolysis of the imine. Therefore the stronger base DBU afforded higher yields than the weaker base triethylamine.

4.1.1.4 Deprotecting agents

Two different deprotecting agents were used: *O*-benzylhydroxylamine (as hydrochloride salt) and benzhydrazide. No difference could be observed between those two reagents. As the hydroxylamine is used as its hydrochloride salt, 1 equivalent of base must be added with it.

4.1.1.5 Concentration

The concentration in all the experiments was high. First a concentration of 0.12 mol/l was used. To improve the yields, the concentration was then increased to 0.18 mol/l but no difference in the isolated yield could be observed. Higher dilution (0.04 mol/l) resulted in lower yields (20% less than in the higher concentrations).

4.1.1.6 Equivalents of SALAL

Usually, 1 equivalent of SALAL was used to form the imine. With 2 equivalents, the yield could be slightly improved (from 60 to 66%) but this complicated the purification of the product afterwards. Therefore, it was decided to use 1 equivalent in all the experiments because the difference in the yield is not that high.

4.1.1.7 Reaction time

The protection (imine formation from SALAL and diamine) was complete after 2 h stirring at rt. Prolonged reaction times (up to 24 h) did not affect the outcome. The reaction of the imine with the 1st electrophile was usually complete after 2 h, except in the cases where no reaction occurred (anthracene methylene chloride, BocLeuOSu) or decomposition took place (acrylonitrile, acetic anhydride). The deprotection was also complete after 2 h at rt. In some cases it could be observed, that longer deprotection times (above 6 h) lead to side reactions which resulted in a lower yield. The reaction with the 2nd electrophile was, like with the 1st one, complete after 2 hours. Prolonged reaction times for the 1st or the 2nd electrophile did not result in lower or higher yields (up to 3 days). The reaction times were independent of the solvent or the base added.

4.1.1.8 Mode of addition

Usually, all reagents were added as liquids or solids to the solution. Mostly a good solubility of the reagents was obtained. To see, if the dilution and the solubility of the reagents affect the yield, one experiment was conducted where all reagents were added as solutions. As some of the reagents had a low solubility, high dilution of the added reagent-solutions was needed. Therefore, the dilution of the reaction mixture was very high, which led to a much lower yield (20% less; see also section "concentration")

4.1.1.9 Without protecting group

The reaction of diphenylcarbamoyl chloride and benzoyl chloride was also tested without the protecting group SALAL. First, diphenylcarbamoyl chloride was added to the unprotected *N*-methylaminopropyl amine and after 2 h, benzoyl chloride was added. The desired product was only obtained in 31% yield. The other 3 possible products (regioisomer of desired product (yield not determinable), twice reacted diphenylcarbamoyl chloride (18%) and twice reacted benzoyl chloride (34%)) were also obtained. This shows the efficiency of the SALAL protection, as the yield of the desired product was 60% under the same reaction conditions (and 75% under optimized reaction conditions).

4.1.2 Mono-reaction with SALAL

4.1.2.1 Electrophiles

Electrophile	Outcome (best yield)
Ph_2NCOCl	70%
PhNCO	86%
p-NO_2BzCl	69%

From the successful bis-reactions, 3 electrophiles were used in mono-reactions on *N*-methylaminopropylamine, yielding in a mono-substitution of the secondary amine. The yields were all in the same range, between 69 and 86%.

4.1.2.2 Solvents

As in the bis-reaction experiments, no difference has been observed between the use of acetonitrile and ethanol in the mono-reactions. THF was tested as another solvent, but no product (*para*-nitrobenzoyl substituted diamine) could be observed. This is probably due to a very poor solubility of the reagents in THF. Therefore, it was not used anymore.

4.1.2.3 Bases

Triethylamine and DBU have been tested in the mono-reactions. Like in the bis-reactions, triethylamine gave moderate to good yields of the corresponding product. With DBU the reactions seemed to proceed well, but unfortunately, it was not possible to isolate any product after reverse phase column chromatography. It seems that DBU is reacting on the reverse phase column with the product and destroys it. Before the reverse phase chromatography, the mass of the corresponding products could be observed in LCMS analyses. As DBU caused these problems, only triethylamine was used as base in the mono-reactions. With 10 mol% DMAP added to the triethylamine, the yield was lower than with triethylamine only.

4.1.2.4 Deprotecting agents

Like for the bis-reactions, no difference could be observed in using *O*-benzylhydroxylamine hydrochloride or benzhydrazide as deprotecting agent.

4.1.2.5 Concentration

Only the two “high” concentrations 0.12 and 0.18 mol/l were tested and no difference in the yield of the corresponding products could be observed.

4.1.2.6 Reaction times

The same results were obtained as for the bis-reaction with SALAL (see above).

4.1.3 Bis-reaction with PYRAL

4.1.3.1 Electrophiles

1st Electrophile	2nd Electrophile	Outcome (best yield)
Ph_2NCOCl	BzCl	No product
CbzCl	PhNCO	6% + regioisomer
PhNCO	CbzCl	74% (+2% regioisomer)
CbzGlyONp	BocAlaOSu	No product
AcCl	Ph_2NCOCl	No product

With PYRAL, the same reactions should be tested as with SALAL. Unfortunately, in most cases, PYRAL did not afford the desired products. With diphenylcarbamoyl chloride, no reaction could be monitored by NMR. This is probably due to a steric hindrance, as the aminal and the carbamoyl chloride are bulky. With benzyl chloroformate and phenyl isocyanate, there was always mostly the undesired regioisomer of the product (up to 82%) and none or a very small amount (up to 6%) of the desired product. NMR showed, that upon addition of the benzyl chloroformate, the aminal is completely transformed into the corresponding imine before reacting with the chloroformate. Therefore, the undesired nitrogen reacts. This problem could not be solved with different solvents or bases (see below). The reaction with phenyl isocyanate and benzyl chloroformate (inverted order) gave 74% of the desired product. This might be due to a higher reactivity of the isocyanate and also due to the fact, that no acid is liberated upon addition of the isocyanate. In this case, the aminal stays intact, which can be seen in the NMR. In the reactions of CbzGlyONp and BocAlaOSu as well as acetyl chloride and diphenylcarbamoyl chloride, no product could be identified. In both cases, the aminal was destroyed after addition of the first electrophile.

4.1.3.2 Solvents

The reactions proceeded best in acetonitrile. In ethanol, protection and deprotection were very slow (up to 2 weeks for the deprotection) but the outcome of the reaction was not affected. In chloroform, the reaction proceeded like in acetonitrile. In toluene, the solubility of the reagents was too low, therefore no product could be isolated. The reaction of benzyl chloroformate and phenyl isocyanate was once conducted in pyridine as solvent. The protection was really fast but the yield of the isolated product was 5 times less than in acetonitrile and mostly the undesired regioisomer (see above) was obtained. Therefore, acetonitrile and chloroform seem to be the best solvents for these reactions.

4.1.3.3 Bases

Generally, aminal formation only became quantitative in presence of 1 equivalent of base. The best base therefore seemed to be triethylamine with 10 mol% DMAP. With triethylamine alone or DBU, aminal formation was much slower but also became quantitative after 18 h.

For the deprotection step, it was important, that no unnecessary additional base was present in the solution. If so, the deprotection became very slow (less than 50% conversion after 3 weeks).

For neutralizing the formed acid from some reagents, DBU was used, as it was already proven to give better results than triethylamine in the reactions with SALAL.

4.1.3.4 Deprotecting agent

No difference was observed between the use of *O*-benzylhydroxylamine hydrochloride and benzhydrazide.

4.1.3.5 Reaction times

In the reactions with PYRAL, protection and deprotection times were, unlike with SALAL, dependent on the solvent and the base added. The protection (aminal formation of PYRAL with diamine) did not complete without the addition of 1 equivalent of base. With triethylamine or DBU in acetonitrile, protection was completed after 18 h, with triethylamine plus 10 mol% DMAP in acetonitrile or chloroform, the protection was already completed after 3 h. In ethanol, reaction was only tested with triethylamine alone and the protection was only completed after several days. The reactions with the 1st electrophile were completed after 2 h in the cases of phenyl isocyanate, benzyl chloroformate, CbzGlyONp and AcCl, although in the last 3 examples, the aminal was converted into the imine and no desired product could be obtained. In the case of diphenylcarbamoyl chloride, no reaction occurred. The deprotection only completed, if no unnecessary additional base was present. In acetonitrile and chloroform the deprotection was complete after 18 h, in ethanol the only after 2 weeks. The reaction with the 2nd electrophile was usually completed after 2 h.

4.1.4 Mono-reaction with PYRAL

4.1.4.1 Electrophile

Electrophile	Outcome
PhNCO	74% (+2 % regioisomer)
Ph_2NCOCl	No product
p-NO_2BzCl	No product
BzCl	No product
Boc_2O	No product

Some of the electrophiles used with SALAL have also been used with PYRAL in mono-reaction. But like for the bis-reaction with PYRAL, the mono-reaction only worked with phenyl isocyanate. As also mentioned for the bis-reaction, diphenylcarbamoyl chloride is probably sterically too hindered to react with the bulky aminal. With *p*-nitrobenzoyl chloride, decomposition was observed upon addition and no product could be obtained. The same result was obtained with benzoyl chloride. As it was thought that the liberated HCl during addition might be the cause of the transformation of the aminal into the imine, di-*t*-butyl-dicarbonate was tested next. This should liberate a weaker acid and therefore, the aminal should be more stable. Unfortunately, this was not the case and complete conversion into the imine was observed.

4.1.4.2 Other aldehydes tested for aminal formation and further reaction

The ability of aminal-formation has been tested with other electron-poor aldehydes than PYRAL. They were applied in the reaction of benzyl chloroformate as 1st electrophile and phenyl isocyanate as 2nd electrophile.

First, para-nitrobenzaldehyde has been used. NMR showed, that the aminal is formed quantitatively with 1 equivalent DBU after 2 h, but upon addition of benzyl chloroformate, it was completely converted into the corresponding imine before reaction with the electrophile. This left the secondary amine free to react with benzyl chloroformate, forming the same product than with SALAL.

Next, *p*-carboxybenzaldehyde was used, but it was not soluble and therefore, the reaction has not been pursued.

With propionaldehyde, the aminal was also formed in presence of 1 equivalent DBU, but again converted into the imine upon addition of benzyl chloroformate.

Hexanal was employed next. It also formed the aminal in presence of 1 equivalent DBU, but after addition of benzyl chloroformate, the NMR became very unclear, so that it could not be determined if aminal was stable or imine was formed. In the end, no product could be identified.

The highly electron withdrawing aldehyde trifluoroacetaldehyde was used next, as it should form a very stable aminal. No additional base was needed to form the aminal, it was quantitative after 2 h. Nevertheless, after addition of benzyl chloroformate, no aminal could be detected, it was converted into the imine again.

As so many aldehydes could not provide the desired product, literature known reaction with formaldehyde was envisaged next. It should be verified if either only formaldehyde gives the desired products or if the reactions described with formaldehyde only work in the described cases (addition of acrylonitrile). Like in the literature, no base was added for the aminal formation, and it was quantitative after 3 h. But again, upon addition of benzyl chloroformate, the aminal was converted into the imine.

A last attempt to obtain a possibility to modify the primary amine before the secondary, was protection with dimethylamino benzaldehyde. This was thought to form in principle the imine with the primary amine but also in a very small amount the iminium with the secondary amine. As the iminium should be much more reactive than the imine, it was thought, that the first would react preferably with benzyl chloroformate. Thus, iminium would be consumed, which would shift the equilibrium from the imine to the iminium side. As it could not be confirmed by NMR if the iminium was formed or stable, reaction was proceeded until the end and the crude was purified by column chromatography. Again, only the regioisomer and not the desired product was obtained, which shows, that either no iminium is formed at all or that it is not reacting preferably with benzyl chloroformate.

4.1.4.3 Solvents

The reactions have been tested in acetonitrile and ethanol and as already observed for the bis-reaction, acetonitrile was the better reagent, as protection and deprotection were too slow in ethanol.

4.1.4.4 Bases

As for the bis-reaction with PYRAL, aminal formation only became quantitative upon addition of 1 equivalent of base. Either triethylamine or DBU gave the aminal quantitative after 18 h. With triethylamine plus 10 mol% DMAP, aminal formation was complete after 3 h. For the deprotection step, it was again important, that no unnecessary additional base was added.

Like in the mono-reaction with SALAL, DBU caused some problems in the reverse phase column chromatography and here again, no product could be obtained. This is why, triethylamine with 10 mol% DMAP were used in these reactions.

4.1.4.5 Deprotecting agent
No difference was observed between *O*-benzylhydroxylamine hydrochloride and benzhydrazide.

4.1.4.6 Reaction times
The same results as for bis-reaction with SALAL were obtained.

4.2 Syntheses

Note: amides and related compounds exhibit hindered rotation around the C-N bond resulting in two rotameric structures observable by NMR as two sets of signals. Splitting of the signals somewhat complicates the spectra in some cases. However, chemical exchange cross-peaks between these two sets of signals in 2D NOESY spectra and coalescence of the two signals into one upon heating to about 65 °C clearly confirms that the relatively complex spectrum (at r.t.) corresponds to the reported structure. In such cases the reported NMR spectrum is given for each rotamer. In all cases the structure was fully characterized by NMR experiments (HSQC, HMBC, COSY and NOESY), mass spectrometry and HPLC-MS analysis. Clean chromatogram and NMR trace served as the proof of purity.

4.2.1 Examples of the SALAL protection procedure

4.2.1.1 Mono-derivatization of N-methyl-1,3-diaminopropane

N-methyl-1,3-diaminopropane (0.30 g, 3.4 mmol) was diluted in acetonitrile (35 mL) and salicylaldehyde (0.37 mL, 1 eq.) was added. The reaction mixture was stirred at room temperature for 2 hours. Then *N*-Cbz-glycine *p*-nitrophenyl ester (1.14 g, 1 eq.) was added as solid and the mixture was stirred at room temperature. After 2 hours, *O*-benzyl hydroxylamine hydrochloride (0.55 g, 1 eq.) together with triethylamine (0.50 mL, 1 eq.) was added and the mixture was stirred for 2 more hours. Then it was concentrated on rotary evaporator an injected on C18 reverse phase column. After injection of the crude, 10 mL of 0.15 M HCl was injected as well to improve the separation. Pure product was obtained by chromatography using acetonitrile-water gradient (0:100 → 95:5) as hydrochloride salt in 82 % yield (0.88 g). The product of the bis-acylation was isolated in 6 % yield (0.09 g).

4.2.1.2 Bis-derivatization of N-methyl-1,3-diaminopropane

N-methyl-1,3-diaminopropane (0.25 g, 2.8 mmol) was diluted in acetonitrile (15 mL) and salicylaldehyde (0.30 mL, 1 eq.) together with triethylamine (0.40 mL, 1 eq.) was added. The reaction mixture was stirred at room temperature for 2 hours. Then *p*-nitrobenzoyl chloride (0.53 g, 1 eq.) was added as solid and the mixture was stirred at room temperature for 2 hours, after which benzhydrazide (0.39 g, 1 eq.) was added and the mixture was stirred for 2 more hours. Then *N*-Cbz-glycine *p*-nitrophenyl ester (0.81 g, 1 eq.) was added as solid and the mixture was again stirred at room temperature for 2 hours. The mixture was then concentrated on rotary evaporator and pure product was obtained by column chromatography on silica using DCM-MeOH gradient elution (99:1 → 90:10), yielding 0.95 g (78 %).

4.2.2 Examples of the PYRAL protection procedure

4.2.2.1 Mono-derivatization of N-methyl-1,3-diaminopropane

N-methyl-1,3-diaminopropane (0.100 g, 1.13 mmol) was diluted in acetonitrile (6 mL) and pyridine-2-carboxaldehyde (0.107 mL, 1 eq.) and triethylamine (0.157 mL, 1 eq.) was added. The reaction mixture was stirred at room temperature for 4 days. Then phenyl isocyanate (0.122 mL, 1 eq.) was added and the mixture was stirred at room temperature. After 3 hours, *O*-benzyl hydroxylamine hydrochloride (0.180 g, 1 eq.) was added and the mixture was stirred for 2 days. Then it was concentrated on rotary evaporator and injected on C18 reverse phase column. The product, as an

inseparable mixture with its regioisomer (23:1), was obtained as yellowish oil by chromatography using acetonitrile-water gradient (0:100 → 95:5) in 77 % yield (0.105 g). Yield product: 74 %, 0.173 g; yield regioisomer: 3.2 %, 0.0075 g.

4.2.2.2 Bis-derivatization of N-methyl-1,3-diaminopropane

N-methyl-1,3-diaminopropane (0.100 g, 1.13 mmol) was diluted in acetonitrile (6 mL) and DBU (0.169 mL, 1 eq.) as well as pyridine-2-carboxaldehyde (0.107 mL, 1 eq.) were added. The reaction mixture was stirred at room temperature for 3 days. Then phenyl isocyanate (0.122 mL, 1 eq.) was added and the mixture was stirred at room temperature. After 2 hours, *O*-benzyl hydroxylamine hydrochloride (0.180 g, 1 eq.) was added and the mixture was stirred for 3 days. Then benzyl chloroformate (0.160 mL, 1 eq.) together with DBU (0.169 mL, 1 eq.) was added. After 4 hours it was concentrated on rotary evaporator and the product was obtained as a mixture with its regioisomer (35:1) by column chromatography on silica using hexane/ethyl acetate gradient elution (88:12 → 0:100) in 76 % yield (0.293 g) as colourless oil. Yield major regioisomer: 0.285 g, 74%; yield minor regioisomer: 8.1 mg, 2.1%.

4.2.3 Synthetic protocols

4.2.3.1 N-(Cbz-glycyl)-N-methyl-1,3-diaminopropane (A1)

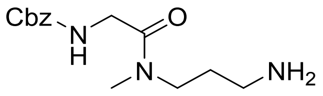

N-methyl-1,3-diaminopropane (0.30 g, 3.4 mmol) was diluted in acetonitrile (35 mL) and salicylaldehyde (0.37 mL, 1 eq.) was added. The reaction mixture was stirred at room temperature for 2 hours. Then *N*-Cbz-glycine *p*-nitrophenyl ester (1.14 g, 1 eq.) was added as solid and the mixture was stirred at room temperature. After 2 hours, *O*-benzyl hydroxylamine hydrochloride (0.55 g, 1 eq.) together with triethylamine (0.50 mL, 1 eq.) was added and the mixture was stirred for 2 more hours. Then it was concentrated on rotary evaporator an injected on C18 reverse phase column. After injection of the crude, 10 mL of 0.15 M HCl was injected as well to improve the separation. Pure product was obtained by chromatography using acetonitrile-water gradient (0:100 → 95:5) as hydrochloride salt in 82 % yield (0.88 g). The product of the bis-acylation was isolated in 6 % yield (0.09 g).

^{1}H-NMR (400 MHz, CD_3CN, 25 °C): 7.45-7.30 (m, 5H; Ar**H**), 5.11 (s, 2H; ArC**H**$_2$), 4.02 + 3.94 (br s, 2H; Gly C**H**$_2$), 3.42 + 3.31 (t, J=6.9 Hz, 2H; NC**H**$_2$), 2.91 + 2.88 (s, 3H; C**H**$_3$), 2.64 + 2.57 (t, J=6.5 Hz, 2H; C**H**$_2$NH$_2$), 1.66 + 1.59 (q, J=6.6 Hz, 2H; CH$_2$C**H**$_2$CH$_2$)

^{13}C-NMR (100 MHz, CD_3CN, 25 °C): 168.47, 168.26, 156.57, 156.50, 137.17, 128.45, 127.88, 127.76, 66.06, 45.84, 44.82, 42.36, 42.12, 38.66, 38.51, 33.34, 32.52, 31.15, 30.45

4.2.3.2 N-(Cbz-glycyl)-N-methyl-N'-(4-nitrobenzoyl)-1,3-diaminopropane (B1)

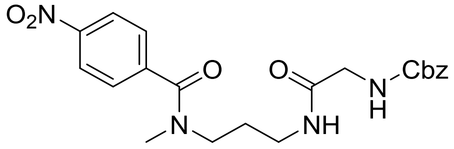

N-methyl-1,3-diaminopropane (0.25 g, 2.8 mmol) was diluted in acetonitrile (15 mL) and salicylaldehyde (0.30 mL, 1 eq.) together with triethylamine (0.40 mL, 1 eq.) was added. The reaction mixture was stirred at room temperature for 2 hours. Then *p*-nitrobenzoyl chloride (0.53 g, 1 eq.) was added as solid and the mixture was stirred at room temperature for 2 hours, after which benzhydrazide (0.39 g, 1 eq.) was added and the mixture was stirred for 2 more hours. Then *N*-Cbz-glycine *p*-nitrophenyl ester (0.81 g, 1 eq.) was added as solid and the mixture was again stirred at room temperature for 2 hours. The

mixture was then concentrated on rotary evaporator and pure product was obtained by column chromatography on silica using DCM-MeOH gradient elution (99:1 → 90:10), yielding 0.95 g (78 %).

^{1}H-NMR (400 MHz, d_6-DMSO, 25 °C): 8.27 (d, J=8.6 Hz, 2H; Ar**H**), 7.72 (br t, 1H; N**H**), 7.66 + 7.59 (d, J=8.3 Hz, 2H; Ar**H**), 7.4-7.2 (m, 5H; Ar**H**), 5.02 + 5.00 (s, 2H; ArC**H**$_2$), 3.58 + 3.44 (d, J=6.0 Hz, 2H; Gly C**H**$_2$), 3.46 + 3.10 (m, 2H; NC**H**$_2$), 3.13 + 2.90 (m, 2H; NC**H**$_2$), 2.96 + 2.83 (s, 3H; C**H**$_3$), 1.73 + 1.63 (q, J=6.7 Hz, 2H; CH_2C**H$_2$**CH_2)

^{13}C-NMR (100 MHz, d_6-DMSO, 25 °C): 169.40, 169.02, 168.80, 156.90, 148.09, 143.49, 137.48, 128.75, 128.54, 128.21, 128.15, 124.13, 79.62, 65.92, 48.65, 44.95, 44.16, 43.92, 37.29, 36.53, 36.19, 32.57, 28.21, 27.07

4.2.3.3 1-(3-aminopropyl)-1-methyl-3,3-diphenylurea (A3)

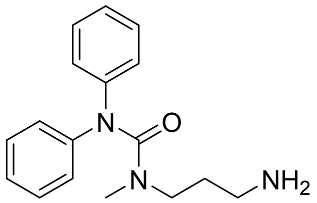

N-methyl-1,3-diaminopropane (0.100 g, 1.13 mmol) was diluted in acetonitrile (6 mL) and salicylaldehyde (0.120 mL, 1 eq.) was added. The reaction mixture was stirred at room temperature for 3 days. Then triethylamine (0.157 mL, 1 eq.) was added and after stirring for 5 min, *N,N*-diphenylcarbamoyl chloride (0.262 g, 1 eq.) was added as solid and the mixture was stirred at room temperature. After 2 hours, benzhydrazide (0.154 g, 1 eq.) was added and the mixture was stirred for 2 more hours. Then it was concentrated on rotary evaporator and injected on C18 reverse phase column. Pure product was obtained as yellowish oil by chromatography using acetonitrile-water gradient (0:100 → 95:5) in 70 % yield (0.225 g).

^{1}H-NMR (400 MHz, $CDCl_3$, 25 °C): 8.50 (s, 2H; N**H**$_2$), 7.32 (t, J=7.8 Hz, 4H; Ar**H**), 7.15 (t, J=7.4 Hz, 2H; Ar**H**), 7.01 (d, J=7.6 Hz, 4H; Ar**H**), 3.41 (t, J=6.0 Hz, 2H; C**H$_2$**NMe), 2.96-3.06 (m, 2H; C**H$_2$**NH_2), 2.56 (s, 3H; C**H**$_3$), 2.07 (quin, J=6.0 Hz, 2H; CH_2C**H$_2$**CH_2)

^{13}C-NMR (100 MHz, $CDCl_3$, 25 °C): 161.84, 144.23, 129.48, 125.35, 125.32, 45.74, 36.90, 35.75, 24.04

MS (ESI, positive mode, 4500 V): 567 (2M$^+$ + H), 284 (M$^+$ + H)

HRMS (ESI, positive mode, 4500 V): calc. (M$^+$ + H): 284.1757; meas. (M$^+$ + H): 284.1757

4.2.3.4 1-(3-aminopropyl)-1-methyl-3-phenylurea (A4)

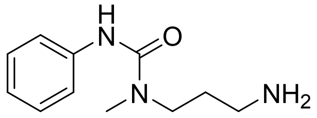

N-methyl-1,3-diaminopropane (0.100 g, 1.13 mmol) was diluted in acetonitrile (6 mL) and salicylaldehyde (0.120 mL, 1 eq.) was added. The reaction mixture was stirred at room temperature for 2 hours. Then phenyl isocyanate (0.122 mL, 1 eq.) was added and the mixture was stirred at room temperature. After 2 hours, *O*-benzyl hydroxylamine hydrochloride (0.180 g, 1 eq.) together with triethylamine (0.157 mL, 1 eq.) was added and the mixture was stirred for 2 more hours. Then it was concentrated on rotary evaporator and injected on C18 reverse phase column. Pure product was obtained as yellowish oil by chromatography using acetonitrile-water gradient (0:100 → 95:5) in 86 % yield (0.201 g).

^{1}H-NMR (400 MHz, MeOD, 25 °C): 7.38 (d, J=8.5 Hz, 2H; Ar**H**), 7.27 (t, J=8.4 Hz, 2H; Ar**H**), 7.05 (t, J=7.4 Hz, 1H; Ar**H**), 3.51 (t, J=6.5 Hz, 2H; C**H$_2$**NMe), 3.08 (s, 3H; C**H**$_3$), 2.96 (t, J=7.4 Hz, 2H; C**H$_2$**NH_2), 1.94 (quin, J=6.6 Hz, 2H; CH_2C**H$_2$**CH_2)

^{13}C-NMR (100 MHz, MeOD, 25 °C): 159.29, 140.58, 129.57, 124.53, 122.74, 45.92, 37.83, 34.82, 26.50

MS (ESI, positive mode, 4500 V): 415 ($2M^+$ + H), 208 (M^+ + H)

HRMS (ESI, positive mode, 4500 V): calc. (M^+ + H): 208.1444; meas. (M^+ + H): 208.1447

4.2.3.5 N-(3-aminopropyl)-N-methyl-4-nitrobenzamide (A2)

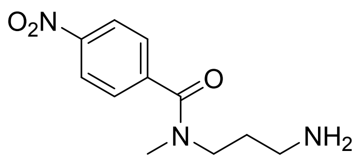

N-methyl-1,3-diaminopropane (0.100 g, 1.13 mmol) was diluted in acetonitrile (6 mL) and salicylaldehyde (0.120 mL, 1 eq.) was added. The reaction mixture was stirred at room temperature for 2 hours. Then triethylamine (0.157 mL, 1 eq.) was added and after stirring for 5 min, *p*-nitrobenzoyl chloride (0.210 g, 1 eq.) was added as solid and the mixture was stirred at room temperature. After 2 hours, *O*-benzyl hydroxylamine hydrochloride (0.180 g, 1 eq.) together with triethylamine (0.157 mL, 1 eq.) was added and the mixture was stirred for 2 more hours. Then it was concentrated on rotary evaporator and injected on C18 reverse phase column. Pure product was obtained as yellowish oil by chromatography using acetonitrile-water gradient (0:100 → 95:5) in 69 % yield (0.184 g).

^{1}H-NMR (400 MHz, MeOD, 25 °C): 8.34 (d, J=8.7 Hz, 2H; Ar**H**), 7.71 (d, J=8.7 Hz, 2H; Ar**H**), 3.69 (t, J=6.7 Hz, 2H; C**H₂**NMe), 3.04 (t, J=7.3 Hz, 2H; C**H₂**NH₂), 3.00 (s, 3H; C**H₃**), 2.06 (quin, J=7.0 Hz, 2H; CH₂C**H₂**CH₂)

^{13}C-NMR (100 MHz, MeOD, 25 °C): 172.25, 150.16, 143.13, 129.32, 124.88, 45.48, 38.20, 37.86, 25.98

MS (ESI, positive mode, 4500 V): 238 (M^+ + H)

HRMS (ESI, positive mode, 4500 V): calc. (M^+ + H): 238.1186; meas. (M^+ + H): 238.1192

4.2.3.6 N-(3-(1-methyl-3,3-diphenylureido)propyl)benzamide (B4)

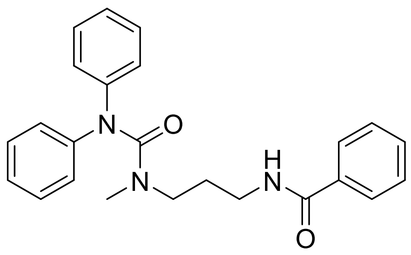

N-methyl-1,3-diaminopropane (0.100 g, 1.13 mmol) was diluted in ethanol (6 mL) and salicylaldehyde (0.120 mL, 1 eq.) was added. The reaction mixture was stirred at room temperature for 2 hours. Then 1,8-diazabicyclo[5.4.0]undecene (DBU; 0.169 mL, 1 eq.) was added and after stirring for 5 min, *N,N*-diphenylcarbamoyl chloride (0.262 g, 1 eq.) was added as solid and the mixture was stirred at room temperature. After 2 hours, *O*-benzyl hydroxylamine hydrochloride (0.180 g, 1 eq.) together with 1,8-diazabicyclo[5.4.0]undecene (DBU; 0.169 mL, 1 eq.) was added and the mixture was stirred for 2 more hours. Then 1,8-diazabicyclo[5.4.0]undecene (DBU; 0.169 mL, 1 eq.) and benzoyl chloride (0.142 mL, 1 eq) were added and the mixture was again stirred for 2 h. Then it was concentrated on rotary evaporator and the product was obtained by column chromatography on silica using hexane/ethyl acetate gradient elution (88:12 → 0:100) as a mixture with a byproduct, where both amines did react with *N,N*-diphenylcarbamoyl chloride. In this case, the amount of byproduct was much less than in the 1st procedure (0.0245 g, 4.5 %) and the yield of the desired product was much higher (0.329 g, 75 %). It is possible to separate the product from the byproduct by chromatography on a C18 column.

^{1}H-NMR (400 MHz, $CDCl_3$, 25 °C): 7.83 (d, J=6.8 Hz, 2H; Ar**H**), 7.77 (s, 1H; NH), 7.44 (t, J=7.3 Hz, 1H; Ar**H**), 7.37 (t, J=6.9 Hz, 2H; Ar**H**), 7.31 (t, J=8.0 Hz, 4H; Ar**H**), 7.14 (t, J=7.6 Hz, 2H; Ar**H**), 7.02 (d, J=7.6 Hz, 4H; Ar**H**), 3.54 (q, J=5.8 Hz, 2H; C**H₂**NH), 3.47 (t, J=6.2 Hz, 2H; C**H₂**NMe), 2.65 (s, 3H; C**H**$_3$), 1.86 (quin, J=6.5 Hz, 2H; CH_2C**H₂**CH_2)

^{13}C-NMR (100 MHz, $CDCl_3$, 25 °C): 167.22, 161.35, 144.57, 134.52, 131.12, 129.34, 128.41, 127.07, 124.81, 124.75, 46.04, 36.07, 35.50, 26.51

MS (ESI, positive mode, 4500 V): 797 (2M$^+$ + Na), 410 (M$^+$ + Na)

HRMS (ESI, positive mode, 4500 V): calc. (M$^+$ + H): 388.2020; meas. (M$^+$ + H): 388.2020

4.2.3.7 Benzyl methyl(3-(3-phenylureido)propyl)carbamate (B5)

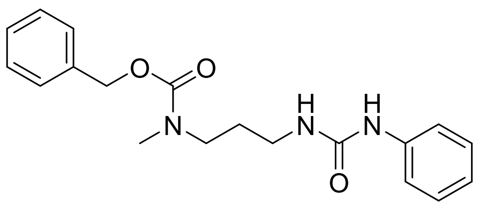

N-methyl-1,3-diaminopropane (0.100 g, 1.13 mmol) was diluted in acetonitrile (6 mL) and salicylaldehyde (0.120 mL, 1 eq.) was added. The reaction mixture was stirred at room temperature for 2 hours. Then DBU (0.169 mL, 1 eq.) was added and after stirring for 5 min, benzyl chloroformate (0.160 mL, 1 eq.) was added and the mixture was stirred at room temperature. After 2 hours, benzhydrazide (0.154 g, 1 eq.) was added and the mixture was stirred for 2 more hours. Then phenylisocyanate (0.122 mL, 1 eq) were added and the mixture was stirred for 18 h. Then it was concentrated on rotary evaporator and the product was obtained pure as white solid by column chromatography on silica using hexane/ethyl acetate gradient elution (88:12 → 0:100) in 71 % yield (0.275 g). In NMR, 2 rotamers (2:1) of the desired products were visible, probably due to the Cbz group. Therefore, sometimes only multiplets could be assigned in ^{1}H NMR.

R_f (hexane/ethyl acetate = 1:1) = 0.31

^{1}H-NMR (400 MHz, $CDCl_3$, 25 °C, major rotamer): 7.32-7.40 (m, 7H; Ar**H**), 7.26-7.29 (m, 2H; Ar**H**)7.00-7.05 (m, 1H; Ar**H**), 6.57 (s, 1H; N**H**Ph), 5.87 (s, 1H; N**H**CH_2), 5.10 (s, 2H; C**H₂**Ph), 3.38 (t, J=6.0 Hz, 2H; C**H₂**NMe), 3.20-3.25 (m, 2H; C**H₂**NH), 2.90 (s, 3H; C**H**$_3$), 1.68-1.72 (m, 2H; CH_2C**H₂**CH_2)

^{1}H-NMR (400 MHz, $CDCl_3$, 25 °C, minor rotamer): 7.32-7.40 (m, 7H; Ar**H**), 7.26-7.29 (m, 2H; Ar**H**), 7.00-7.05 (m, 1H; Ar**H**), 6.57 (s, 1H; N**H**Ph), 5.87 (s, 1H; N**H**CH_2), 5.10 (s, 2H; C**H₂**Ph), 3.28-3.36 (m, 2H; C**H₂**NMe), 3.13-3.20 (m, 2H; C**H₂**NH), 2.90 (s, 3H; C**H**$_3$), 1.68-1.72 (m, 2H; CH_2C**H₂**CH_2)

^{13}C-NMR (100 MHz, $CDCl_3$, 25 °C, major rotamer): 156.99, 155.90, 139.09, 136.57, 128.98, 128.49, 128.03, 127.60, 122.88, 119.90, 67.21, 45.93, 36.39, 33.84, 27.34

^{13}C-NMR (100 MHz, $CDCl_3$, 25 °C, minor rotamer): 156.09, 155.67, 138.87, 136.57, 128.98, 128.71, 128.26, 127.60, 123.11, 120.13, 67.21, 45.93, 36.91, 34.45, 28.08

MS (ESI, positive mode, 4500 V): 705 (2M$^+$ + Na), 364 (M$^+$ + Na)

HRMS (ESI, positive mode, 4500 V): calc. (M$^+$ + H): 342.1812; meas. (M$^+$ + H): 342.1762

Melting point: 96 °C

4.2.3.8 benzyl (3-(1-methyl-3-phenylureido)propyl)carbamate (B6)

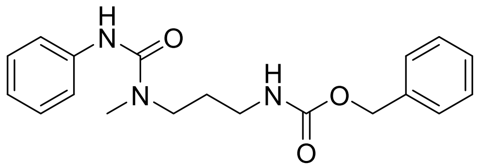

N-methyl-1,3-diaminopropane (0.100 g, 1.13 mmol) was diluted in ethanol (6 mL) and salicylaldehyde (0.120 mL, 1 eq.) was added. The reaction mixture was stirred at room temperature for 6 hours. Then phenylisocyanate (0.122 mL, 1 eq.) was added and the mixture was stirred at room temperature. After 18 hours, *O*-benzyl hydroxylamine hydrochloride (0.180 g, 1 eq.) together with triethylamine (0.157 mL, 1 eq.) was added and the mixture was stirred for 2 hours. Then triethylamine (0.157 mL, 1 eq.) and benzyl chloroformate (0.160 mL, 1 eq.) were added and the mixture was stirred for 2 more hours. Then it was concentrated on rotary evaporator and the product was obtained pure as yellow solid by column chromatography on silica using hexane/ethyl acetate gradient elution (88:12 → 0:100) in 82 % yield (0.316 g).

^{1}H-NMR (400 MHz, $CDCl_3$, 25 °C): 7.28-7.38 (m, 9H; Ar**H**), 7.04 (t, J=7.3 Hz, 1H; Ar**H**), 6.48 (s, 1H; N**H**CON), 5.72 (s, 1H; N**H**CO_2), 5.10 (s, 2H; C**H₂**Ph), 3.46 (t, J=6.3 Hz 2H; C**H₂**NMe), 3.21 (m, 2H; C**H₂**NH), 2.98 (s, 3H; C**H**₃), 1.73 (quin, J=6.0 Hz, 2H; CH_2C**H₂**CH_2)

^{13}C-NMR (100 MHz, $CDCl_3$, 25 °C): 156.68, 155.91, 138.90, 136.71, 128.86, 128.43, 128.02, 127.96, 123.17, 120.11, 66.51, 45.62, 37.57, 34.29, 27.88

MS (ESI, positive mode, 4500 V): 705 (2M^+ + Na), 653 (2M^+ - COH), 364 (M^+ + Na), 312 (M^+ - COH)

HRMS (ESI, positive mode, 4500 V): calc. (M^+ + Na): 364.1632; meas. (M^+ + Na): 364.1632

Melting point: 101 °C

4.2.3.9 benzyl (2,2,6,12-tetramethyl-4,7,13-trioxo-3-oxa-5,8,12-triazatetradecan-14-yl)carbamate (B3)

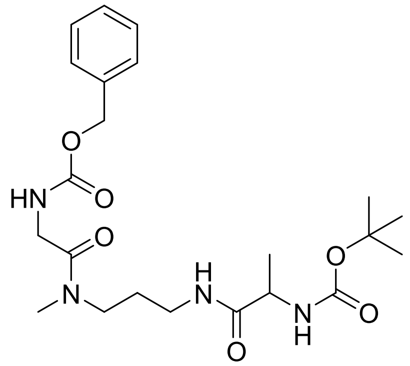

N-methyl-1,3-diaminopropane (0.100 g, 1.13 mmol) was diluted in acetonitrile (6 mL) and salicylaldehyde (0.120 mL, 1 eq.) was added. The reaction mixture was stirred at room temperature for 2 hours. Then CbZ-Gly-ONp (Benzyl oxycarbonyl protected glycine *para*-nitrophenolester; 0.373 g, 1 eq.) was added and the mixture was stirred at room temperature. After 4 hours, benzhydrazide (0.154 g, 1 eq.) was added and the mixture was stirred for 2 hours. Then *tert*-butyloxycarbonyl protected alanine-*O*-succinimide (BocAlaOSu; 0.323 g, 1 eq.) were added and the mixture was stirred for 18 hours. Then it was concentrated on rotary evaporator and purified by column chromatography on silica using hexane/ethyl acetate gradient elution (88:12 → 0:100) followed by ethyl acetate/methanol gradient elution (100:0 → 0:100). Then, the product was repurified by reverse phase column chromatography on a C18-column. It was obtained pure as yellowish oil in 82% yield (0.419 g, 0.931 mmol). At room temperature, the NMR showed at least 2 rotamers because of the nitrogen protecting groups. Heating the NMR tube to 70 °C gave a nice spectrum with one signal set. Therefore, NMR data is given at 70 °C.

R_f (ethyl acetate pure) = 0.12

^{1}H-NMR (400 MHz, $CDCl_3$, 70 °C): 7.55-7.63 (m, 1H; N**H**COCHCH$_3$), 7.34-7.38 (m, 5H; Ar**H**), 6.93 (bs, 1H; N**H**CO$_2$Bn), 6.52 (bs, 1H; N**H**CO$_2$*t*Bu), 5.05 (s, 2H; C**H$_2$**Ph), 3.93 (quin, J=7.1 Hz, 1H; C**H**CH$_3$), 3.86 (d, J=5.8 Hz, 2H; Gly C**H**$_2$), 3.24-3.35 (m, 2H; C**H$_2$**NMe), 3.12 (s, 3H; C**H**$_3$N), 3.00-3.15 (m, 2H, C**H$_2$**NH), 1.55-1.74 (m, 2H, CH$_2$C**H$_2$**CH$_2$), 1.39 (s, 9H; *t*Bu), 1.20 (d, J=7.1 Hz, 3H; C**H$_3$**CH)

^{13}C-NMR (100 MHz, $CDCl_3$, 70 °C, major rotamer): 172.19, 168.02, 155.97, 154.59, 136.86, 127.92, 127.31, 127.18, 77.77, 65.12, 49.75, 44.87, 41.92, 35.86, 33.51, 27.89, 26.63, 18.01

MS (ESI, positive mode, 4500 V): 923 (2M$^+$ + Na), 473 (M$^+$ + Na)

HRMS (ESI, positive mode, 4500 V): calc. (M$^+$ + Na): 473.2303; meas. (M$^+$ + Na): 473.2371

4.2.3.10 benzyl (6-isobutyl-2,2,12-trimethyl-4,7,13-trioxo-3-oxa-5,8,12-triazatetradecan-14-yl)carbamate (B2)

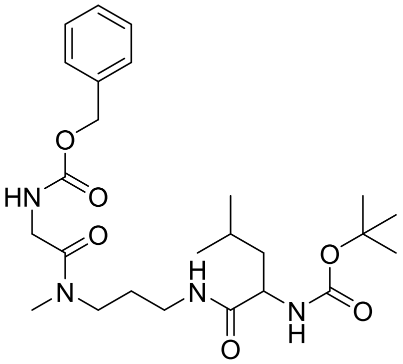

N-methyl-1,3-diaminopropane (0.100 g, 1.13 mmol) was diluted in acetonitrile (6 mL) and salicylaldehyde (0.120 mL, 1 eq.) was added. The reaction mixture was stirred at room temperature for 16 hours. Then CbZ-Gly-ONp (Benzyl oxycarbonyl protected glycine *para*-nitrophenolester; 0.373 g, 1 eq.) was added and the mixture was stirred at room temperature. After 3 hours, benzhydrazide (0.154 g, 1 eq.) was added and the mixture was stirred for 4 hours. Then *tert*-butyloxycarbonyl protected leucine-*O*succinimide (BocLeuOSu; 0.371 g, 1 eq.) were added and the mixture was stirred for 17 hours. Then it was concentrated on rotary evaporator and purified by column chromatography on silica using hexane/ethyl acetate gradient elution (88:12 → 0:100) followed by ethyl acetate/methanol gradient elution (100:0 → 0:100). Then, the product was repurified by reverse phase column chromatography on a C18-column. It was obtained pure as yellowish oil in 83% yield (0.462 g, 0.938 mmol). At room temperature, the NMR showed at least 2 rotamers because of the nitrogen protecting groups. NMR data is given of the major rotamer.

R_f (ethyl acetate pure) = 0.31

^{1}H-NMR (400 MHz, $CDCl_3$, 25 °C, major rotamer): 7.87 (t, J=5.3 Hz, 1H; N**H**CO), 7.33-7.38 (m, 5H; Ar**H**), 7.20 (t, J=5.6 Hz, 1H; N**H**CO$_2$Bn), 6.83 (t, J=6.6 Hz, 1H; N**H**CO$_2$*t*Bu), 5.03 (s, 2H; C**H$_2$**Ph), 3.87-3.92 (m, 1H; C**H**NH), 3.83 (d, J=6.0 Hz, 2H; Gly C**H**$_2$), 3.21-3.29 (m, 2H; C**H$_2$**N), 2.96-3.05 (m, 2H, C**H$_2$**N), 2.91 (s, 3H; C**H**$_3$N), 1.52-1.60 (m, 3H, CH$_2$C**H$_2$**CH$_2$, C**H**(CH$_3$)$_2$), 1.39-1.45 (m, 2H; CHC**H$_2$**CH), 1.37 (s, 9H; *t*Bu), 0.86 (d, J=7.7 Hz, 6H; (C**H$_3$**)$_2$CH)

^{13}C-NMR (100 MHz, $CDCl_3$, 25 °C, major rotamer): 172.33, 168.35, 155.27, 137.12, 128.26, 127.67, 77.86, 65.31, 52.87, 45.75, 42.04, 40.83, 36.07, 33.81, 28.13, 26.86, 24.25, 22.85

MS (ESI, positive mode, 4500 V): 1007 (2M$^+$ + Na), 515 (M$^+$ + Na)

HRMS (ESI, positive mode, 4500 V): calc. (M$^+$ + Na): 515.2840; meas. (M$^+$ + Na): 515.2817

4.2.3.11 1-(3-(methylamino)propyl)-3-phenylurea (C1)

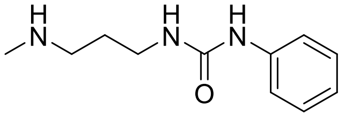

N-methyl-1,3-diaminopropane (0.100 g, 1.13 mmol) was diluted in acetonitrile (6 mL) and pyridine-2-carboxaldehyde (0.107 mL, 1 eq.) and triethylamine (0.157 mL, 1 eq) was added. The reaction mixture was stirred at room temperature for 4 days. Then phenyl isocyanate

(0.122 mL, 1 eq.) was added and the mixture was stirred at room temperature. After 3 hours, *O*-benzyl hydroxylamine hydrochloride (0.180 g, 1 eq.) was added and the mixture was stirred for 2 days. Then it was concentrated on rotary evaporator and injected on C18 reverse phase column. The product, as an inseparable mixture with its regioisomer (23:1), was obtained as yellowish oil by chromatography using acetonitrile-water gradient (0:100 → 95:5) in 77 % yield (0.105 g). Yield product: 74 %, 0.173 g; yield regioisomer: 3.2 %, 0.0075 g.

^{1}H-NMR (400 MHz, MeOD, 25 °C; major regioisomer): 7.38 (d, J=7.4 Hz, 2H; Ar**H**), 7.3 (t, J= 8.0 Hz, 2H; Ar**H**), 6.99 (t, J=7.3 Hz, 1H; Ar**H**), 3.30-3.33 (m, 2H; C**H$_2$**NHCO (signal overlap with solvent)), 3.05 (t, J=7.3 Hz, 2H, C**H$_2$**NHMe), 2.71 (s, 3H; C**H**$_3$), 1.89 (quin, J=6.5 Hz, 2H; CH$_2$C**H$_2$**CH$_2$)

^{1}H-NMR (400 MHz, MeOD, 25 °C; minor regioisomer): 7.40-7.42 (m, 2H; Ar**H**), 7.27-7.31 (m, 2H; Ar**H**), 7.06 (t, J=7.4 Hz, 1H; Ar**H**), 3.53 (t, J=6.5 Hz, 2H; C**H$_2$**-NMe), 3.10 (s, 3H; C**H**$_3$), 2.98 (t, J=7.0 Hz, 2H; C**H$_2$**NH$_2$), 1.96 (quin, J=6.8 Hz, 2H; CH$_2$C**H$_2$**CH$_2$)

^{13}C-NMR (100 MHz, MeOD, 25 °C; major regioisomer): 158.97, 140.72, 129.81, 123.67, 120.38, 47.85, 37.18, 33.61, 28.34

^{13}C-NMR (100 MHz, MeOD, 25 °C; minor regioisomer): 159.25, 140.58, 129.56, 124.50, 122.74, 45.96, 37.86, 34.85, 26.52

MS (ESI, positive mode, 4500 V): 415 (2M$^+$ + H), 208 (M$^+$ + H)

HRMS (ESI, positive mode, 4500 V): calc. (M$^+$ + H): 208.1444 meas. (M$^+$ + H): 208.1454

4.2.3.12 benzyl methyl(3-(3-phenylureido)propyl)carbamate (C2)

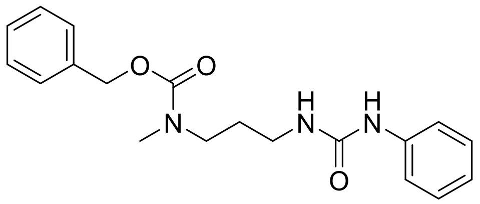

N-methyl-1,3-diaminopropane (0.100 g, 1.13 mmol) was diluted in acetonitrile (6 mL) and DBU (0.169 mL, 1 eq.) as well as pyridine-2-carboxaldehyde (0.107 mL, 1 eq.) were added. The reaction mixture was stirred at room temperature for 3 days. Then phenyl isocyanate (0.122 mL, 1 eq.) was added and the mixture was stirred at room temperature. After 2 hours, O-benzyl hydroxylamine hydrochloride (0.180 g, 1 eq.) was added and the mixture was stirred for 3 days. Then benzyl chloroformate (0.160 mL, 1 eq.) together with DBU (0.169 mL, 1 eq.) was added. After 4 hours it was concentrated on rotary evaporator and the product was obtained as a mixture with its regioisomer (35.0:1.0) by column chromatography on silica using hexane/ethyl acetate gradient elution (88:12 → 0:100) in 76 % yield (0.293 g). Yield major regioisomer: 0.285 g, 74%; yield minor regioisomer: 8.1 mg, 2.1%. White oil. The major regioisomer is in addition composed of two rotamers (2:1). Therefore, some signals in the ^{1}H NMR can only be assigned as multiplets.

R_f (hexane/ethyl acetate = 1:1) = 0.31

^{1}H-NMR (400 MHz, CDCl$_3$, 25 °C; major regioisomer, major rotamer): 7.25-7.40 (m, 9H; Ar**H**), 7.01-7.06 (m, 1H; Ar**H**), 5.12 (s, 2H; C**H$_2$**Ph), 3.37 (t, J=5.6 Hz, 2H; C**H$_2$**NMe), 3.19-3.25 (m, 2H, C**H$_2$**NH), 2.92 (s, 3H; C**H**$_3$), 1.66-1.74 (m, 2H; CH$_2$C**H$_2$**CH$_2$)

^{1}H-NMR (400 MHz, $CDCl_3$, 25 °C; major regioisomer, minor rotamer): 7.25-7.40 (m, 9H; Ar**H**), 7.01-7.06 (m, 1H; Ar**H**), 5.12 (s, 2H; C**H₂**Ph), 3.29-3.35 (m, 2H; C**H₂**NMe), 3.12-3.20 (m, 2H, C**H₂**NH), 2.92 (s, 3H; C**H**₃), 1.66-1.74 (m, 2H; CH_2C**H₂**CH_2)

^{1}H-NMR (400 MHz, $CDCl_3$, 25 °C; minor regioisomer): 7.25-7.40 (m, 9H; Ar**H**), 7.01-7.06 (m, 1H; Ar**H**), 6.62 (s, 1H; N**H**Ph), 5.78 (s, 1H, N**H**CH_2), 5.12 (s, 2H; C**H₂**Ph), 3.47 (t, J=6.4 Hz, 2H; C**H₂**NMe), 3.19-3.25 (m, 2H, C**H₂**NH), 3.00 (s, 3H; C**H**₃), 1.66-1.77 (m, 2H; CH_2C**H₂**CH_2)

^{13}C-NMR (100 MHz, $CDCl_3$, 25 °C; major regioisomer, major rotamer): 156.99, 155.85, 139.10, 136.58, 128.96, 128.50, 128.04, 127.61, 122.85, 119.82, 67.25, 45.91, 36.33, 33.83, 27.32

^{13}C-NMR (100 MHz, $CDCl_3$, 25 °C, major regioisomer, minor rotamer): 156.12, 155.62, 138.89, 136.58, 128.96, 128.73, 128.34, 128.26, 123.06, 120.06, 67.25, 45.91, 36.89, 34.44, 28.06

^{13}C-NMR (100 MHz, $CDCl_3$, 25 °C, minor regioisomer): 156.73, 156.03, 138.89, 136.64, 128.83, 128.42, 127.97, 127.95, 123.20, 120.23, 66.52, 45.67, 37.60, 34.27, 27.90

MS (ESI, positive mode, 4500 V): 705 ($2M^+$ + Na), 364 (M^+ + Na)

HRMS (ESI, positive mode, 4500 V): calc. (M^+ + Na): 364.1632 meas. (M^+ + Na): 364.1627

4.2.3.13 t-butyl (S)-(1-(methyl(3-(3-phenylureido)propyl)amino)-1-oxo-3-phenylpropan-2-yl)carbamate (C3)

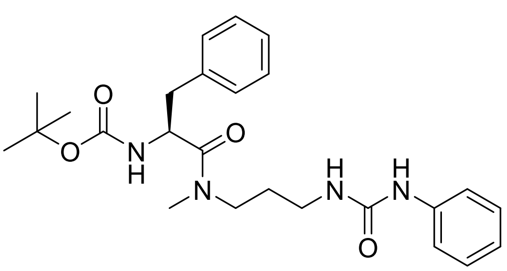

N-methyl-1,3-diaminopropane (0.100 g, 1.13 mmol) was diluted in acetonitrile (6 mL) and DBU (0.169 mL, 1 eq.) as well as pyridine-2-carboxaldehyde (0.107 mL, 1 eq.) were added. The reaction mixture was stirred at room temperature for 18 hours. Then phenyl isocyanate (0.122 mL, 1 eq.) was added and the mixture was stirred at room temperature. After 2 hours, *O*-benzyl hydroxylamine hydrochloride (0.180 g, 1 eq.) was added and the mixture was stirred for 18 hours. Then BocPheOSu (0.409 g, 1 eq.) was added. After 4 hours it was concentrated on rotary evaporator and the product was obtained by column chromatography on silica using hexane/ethyl acetate gradient elution (88:12 → 0:100) and then ethyl acetate/methanol gradient elution (100:0 → 0:100) in 76 % yield (0.390 g). Together with the desired product, the corresponding regioisomer was isolated in 9% yield (0.046 g). The regioisomers could not be separated and were obtained as greenish oil. Both regioisomers are composed of 2 rotamers. Here, NMR data is only given for the major rotamer of the desired product.

R_f (pure ethyl acetate) = 0.67

^{1}H-NMR (400 MHz, DMSO-d_6, 25 °C; major rotamer of desired product): 8.59 (bs, 1H, N**H**), 7.37-7.41 (m, 2H; Ar**H**), 7.07-7.27 (m, 8H, Ar**H** + N**H**), 6.85-6.94 (m, 1H, Ar**H**), 6.12 (t, J=5.2 Hz, 1H, N**H**), 4.52 (m, 1H, C**H**NHBoc), 3.28 (t, J=6.3 Hz, 2H; C**H₂**NH), 2.73-3.06 (m, 4H, C**H₂**NMe + C**H**₂Ph), 2.88 (s, 3H; C**H**₃), 1.52 (quin, J=6.2 Hz, 2H; CH_2C**H₂**CH_2), 1.31 (s, 9H, *t*Bu)

^{13}C-NMR (100 MHz, DMSO-d_6, 25 °C; major rotamer of desired product): 171.67, 155.34, 155.26, 140.70, 137.74, 129.37, 128.66, 128.14, 126.44, 120.96, 117.67, 78.16, 51.99, 44.98, 36.34, 34.64, 33.36, 28.20, 27.39

MS (ESI, positive mode, 4500 V): 931 ($2M^+$ + Na), 477 (M^+ + Na)

HRMS (ESI, positive mode, 4500 V): calc. (M^+ + Na): 477.2472 meas. (M^+ + Na): 477.2490

4.3 NMR spectra of key intermediates and experiments

4.3.1 Intermediates in SALAL and PYRAL protection

Reactions in acetonitrile were followed by NMR after each step by taking 4 µL of the reaction mixture and diluting it in 0.5 mL of $CDCl_3$. No solvent suppression was used because the informative signals are well separated from the strong acetonitrile signal at ~2 ppm.

Salicylaldehyde reacts with **MeDAP** to afford the imine formed on the primary amino group. Presence of a second amino group in proximity leads to dynamic intramolecular exchange between the imine and the aminal form. For the cases of salicylaldehyde the amount of the aminal form has been previously estimated[4] to around 3 %, whereas the imine as the major product is present in 97 %. This dynamic equilibrium leads to dramatic broadening of corresponding signals in NMR spectra.

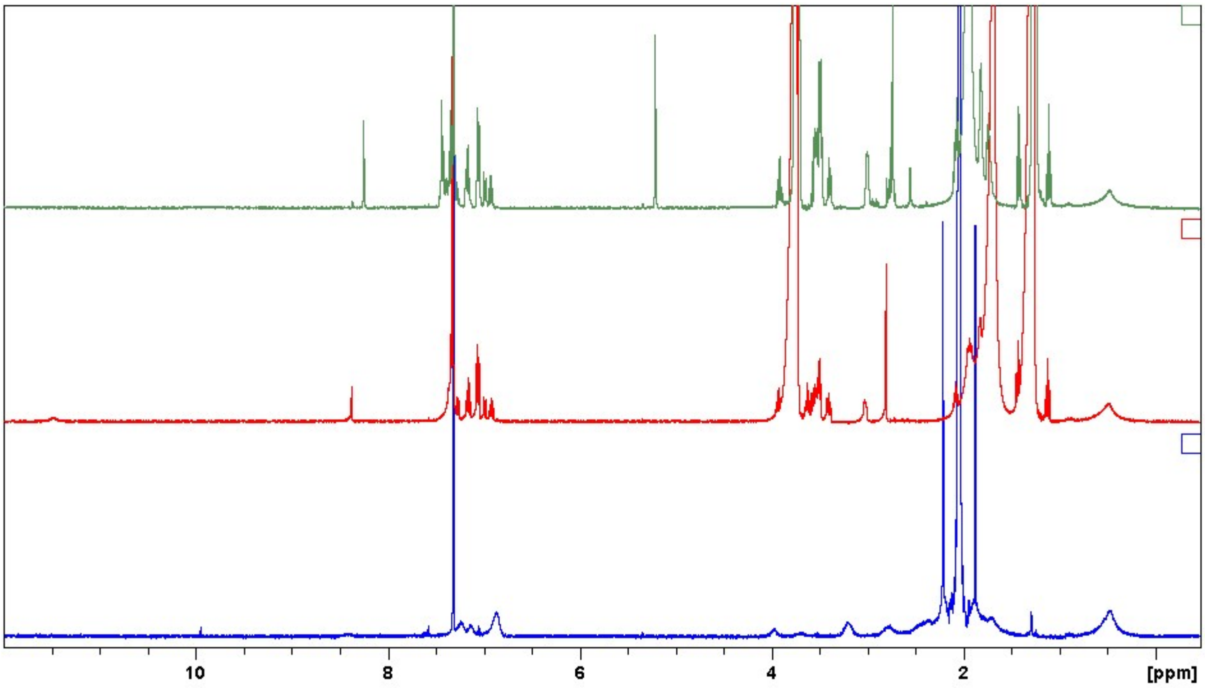

*Figure 18, NMR spectra of **SALAL** protected derivatization of **MeDAP**. Blue trace was recorded after mixing **MeDAP** and **SALAL**, dynamic exchange between aminal and imine results in broad signals. Red trace is the reaction mixture after the addition of N,N-diphenylcarbamoyl chloride – sharp imine signal at 8.4 ppm shows that the intramolecular exchange process does not occur anymore. Also the sharp singlet at 2.7 ppm, corresponding to the methyl group of derivatized **MeDAP**, indicates that only one product has been formed. Green trace is the reaction mixture after addition of O-benzylhydroxylamine as the deprotecting agent. The shift of the imine signal is indicative for the formation of the oxime. Sharp singlet at 5.1 ppm (benzylic CH_2) shows complete conversion of the imine to the oxime.*

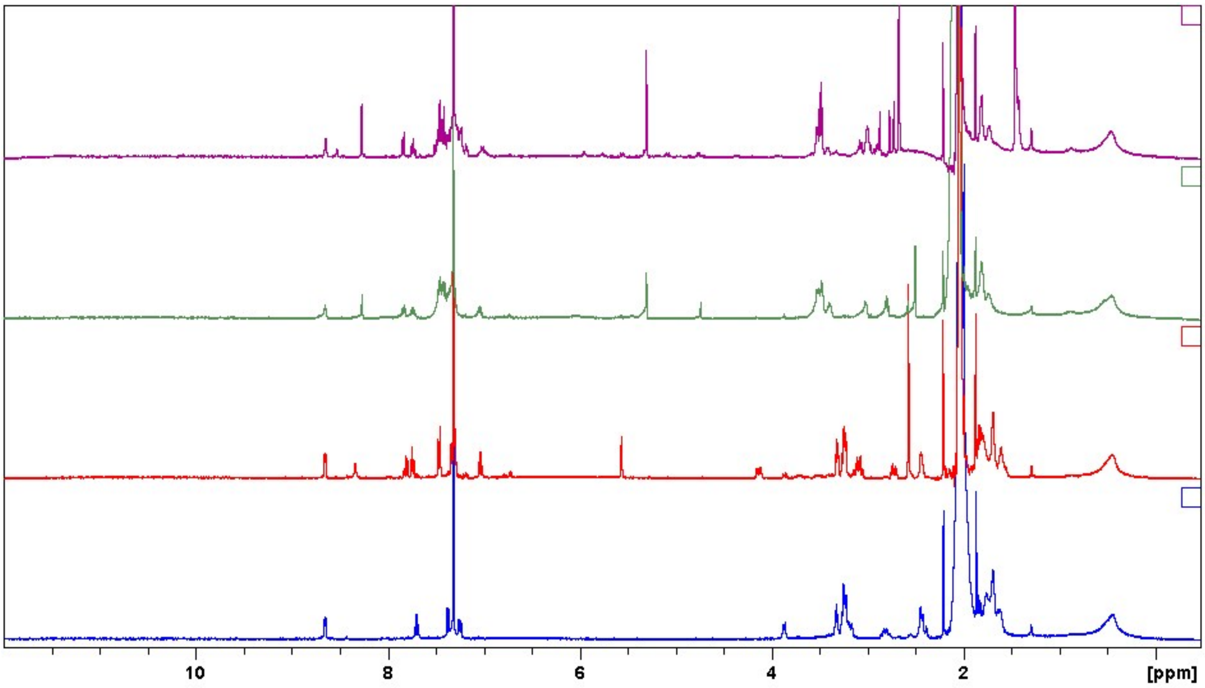

*Figure 19, NMR spectra of **PYRAL** protected derivatization of **MeDAP**. Blue trace is after mixing **PYRAL** + **MeDAP** – only one sharp set of signals corresponding to the aminal formed is observed. Red trace is after the addition of phenyl isocyanate – the aminal peak has shifted from 3.9 to 5.5 ppm indicating the derivatization of the nitrogen atom, as well as the signal at 8.4 ppm which belongs to the urea NH proton. Green trace is the deprotection by O-benzylhydroxylamine, oxime singlets at 8.3 and 5.3 ppm (CH=NO and benzylic CH_2 protons, respectively). Purple trace is the reaction after addition of Boc-PheOSu.*

4.4 Acrylonitrile Michael addition

Addition of acrylonitrile to amines proceeds quickly in methanol which enables quick proton transfer. In the case of reactions in NMR tube, which were performed in d_4-methanol quantitative deuteration of the carbon next to nitrile group was observed due to the proton transfer from protic solvent.

4.4.1.1 N,N'-bis(2-cyanoethyl)-1,3-diaminopropane

1,3-Diaminopropane (1.00 ml, 11.98 mmol) was dissolved in 50 ml of methanol and acrylonitrile (1.57 ml, 23.96 mmol) was added. The reaction mixture was stirred for 24 hours then the solvent was evaporated. Colourless oil (2.15 g, quantitative yield) was obtained in sufficient purity.

^{1}H-NMR (400 MHz, MeOD, 25 °C): 2.89 (t, J=6.8 Hz, 4H, C**H**$_2$CN); 2.71 (t, J=7.0 Hz, 4H, C**H**$_2$NHCH$_2$CH$_2$CN); 2.63 (t, J=6.8 Hz, 4H, CH$_2$NHC**H**$_2$CH$_2$CN); 1.72 (q, J=7.0 Hz, 2H, C**H**$_2$)

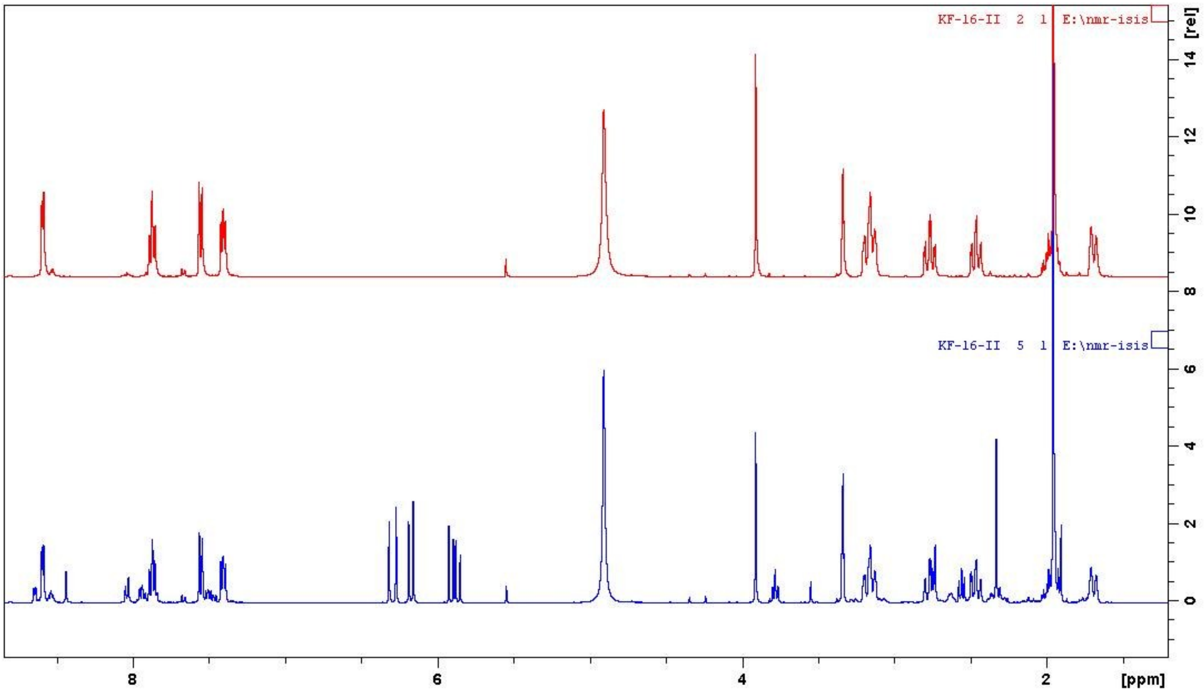

*Figure 20. NMR spectra in d_4-methanol of **MeDAP** protected with **PYRAL** (red spectrum) where formation of aminal can be clearly detected. After 24 hours of reaction with acrylonitrile, two products can be observed in low yields- approximately 8 % of expected addition to aminal NH group and 19 % of addition to secondary amino group due to equilibrium between aminal and imine form (blue spectrum).*

Red spectrum: 8.6 (d, 1H, J= 4.5 Hz, ArH), 7.9 (t, 1H, J=7.5 Hz, ArH), 7.6 (d, 1 H, J= 7.5 Hz, ArH), 4.9 (br s, H_2O), 3.9 (1H, s, aminal), 3.3 (m, solvent peak), 3.2 (t, 2H, J=13.1 Hz, CH_2), 2.8 (t, 1H, J=13.1 Hz, CH_2), 2.5 (t, 1H, J= 12.3 Hz, CH_2), 1.9-2.1 (m+s, 4 H, CH_2+CH_3), 1.7 (d, 1H, J=12.3, CH_2) ppm.

Blue spectrum: acrylonitrile: 6.3 (d, 1 H, J= 17.8 Hz), 6.2 (d, 1 H, J=11.5 Hz), 5.9 (dd, 1H, J_1=117.8, J_2=11.5 Hz) ppm, important peaks in the mixture are assigned in Figure 21.

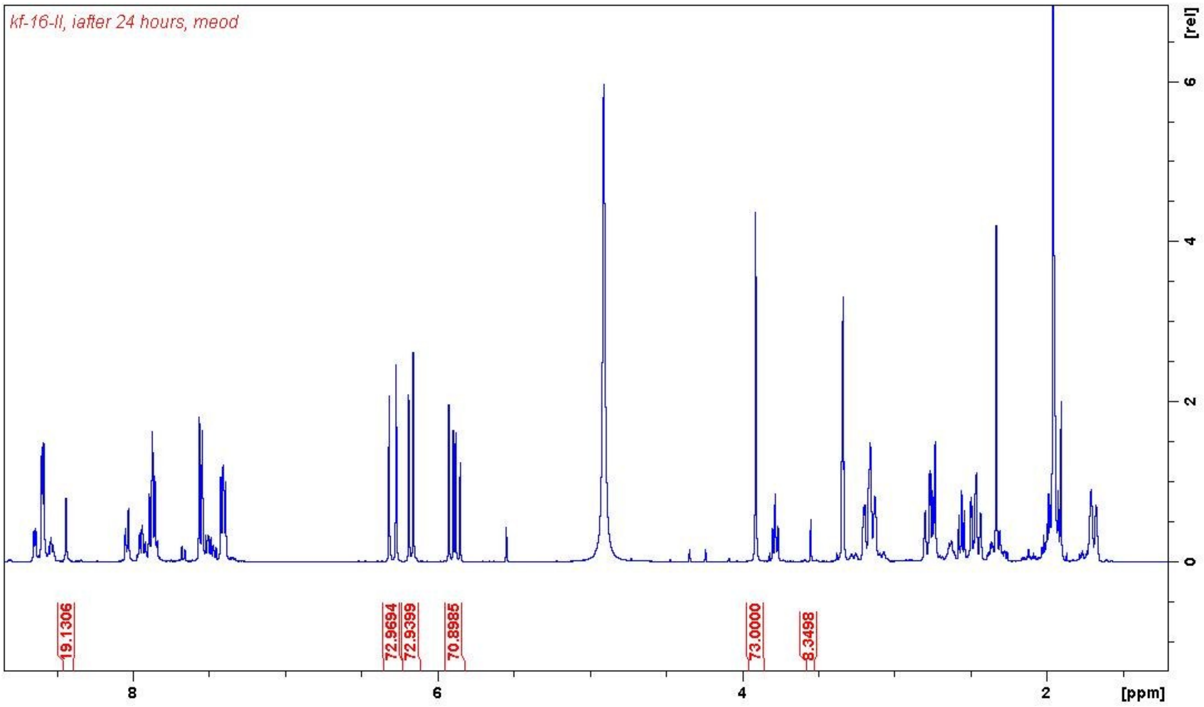

*Figure 21, NMR spectrum of **MeDAP** protected with **PYRAL** after 24 hours of reaction with acrylonitrile in d4-methanol. Important peaks are integrated: imine peak of the product of addition to secondary amino group (8.44 ppm), peaks of*

*the unreacted acrylonitrile (d 6.30 ppm, d 6.18 ppm, dd 5.90 ppm), aminal peak of unreacted **MeDAP** protected with **PYRAL** (3.91 ppm) and aminal peak of desired aminal after the addition of acrylonitrile to aminal NH group (3.55 ppm).*

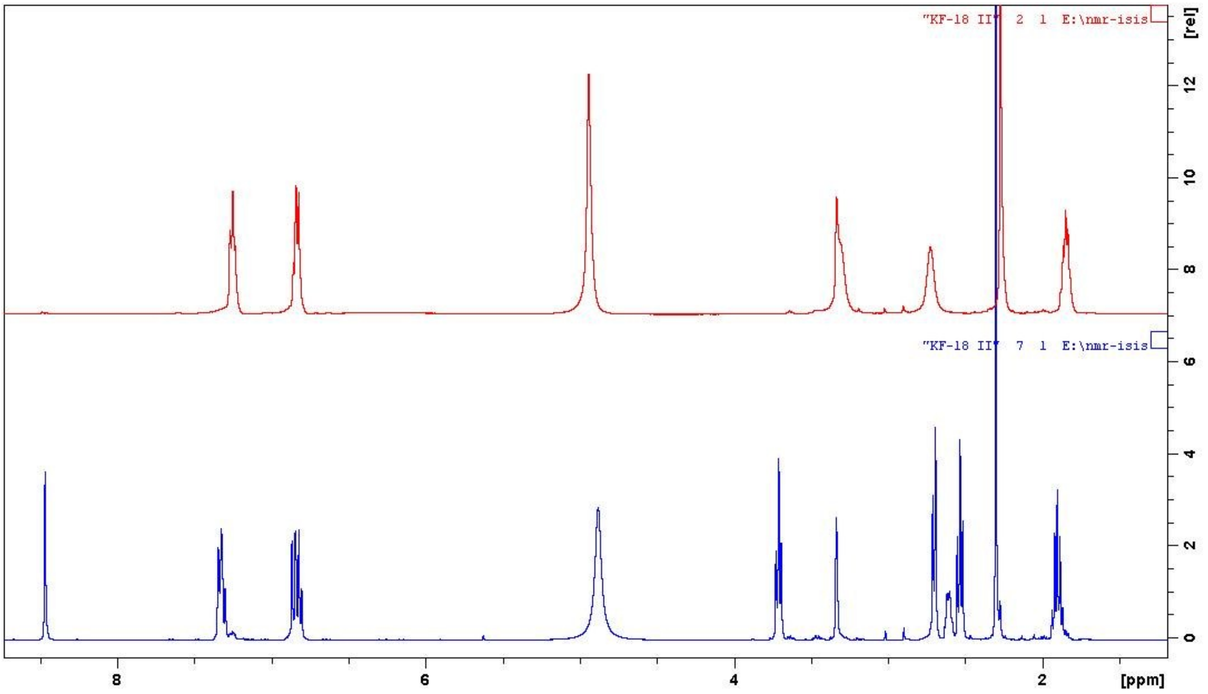

*Figure 22. NMR spectra in d_4-methanol of **MeDAP** protected with **SALAL** (red spectrum), the imine proton cannot be observed due to the quick interconversion between aminal and imine form. After 24 hours the acrylonitrile completely reacted with protected amine (blue spectrum) and the signal of imine hydrogen can be clearly observed at 8.49 ppm which proves that the addition of acrylonitrile takes place on the secondary amine.*

Red spectrum: 7.3 (br, 2H, ArH), 6.8 (m, 2H, ArH), 4.9 (br s, H_2O), 3.3 (br, CH_2+solvent peak), 2.7 (br, 2H, CH_2), 2.3 (s, 3H, CH_3), 1.8 (br, 2H, CH_2) ppm.

Blue spectrum: 8.5 (s, 1H, imine), 7.3 (m, 2H, ArH), 6.8 (m, 2H, ArH), 4.9 (br s, H_2O),3.7 (t, 2H, J=6.8 Hz, CH2), 3.3 (m, solvent peak), 2.7 (d, 2H, J=6.8 Hz, CH_2), 2.6 (br, 1H, CH_2CHD-CN-deuteration takes place during addition reaction), 2.5 (t, 2H, J=6.5 Hz, CH_2), 2.3 (s, 3H, CH_3), 1.9 (m, 2H, CH_2) ppm.

4.5 Selective derivatisation of triamines

The possibility of the use of **SALAL** and **PYRAL** in selective protection of polyamines opens new way for their simple derivatisation. Reactions of several triamines – diethylenetriamine (**en_2N_3**), bis(3-amiopropyl)amine (**pr_2N_3**) and spermidine were studied first in NMR tube in d_3-acetonitrile and obtained results were used for the development of the subsequent preparative derivatisation procedure without isolation of intermediates.

4.5.1 NMR equilibration study

Because of the aforementioned insufficient reactivity of the aminal NH group, the selective aminal formation was investigated in scope of concomitant protection of one secondary and one primary amino group. NMR experiments in d_3-acetonitrile showed that it is possible to prepare the desired aminal with 1 equivalent of **PYRAL**. NOESY spectra revealed that interconversion between aminal and imine takes place, but aminal form is strongly favoured as can be seen from integral intensities of corresponding signals.

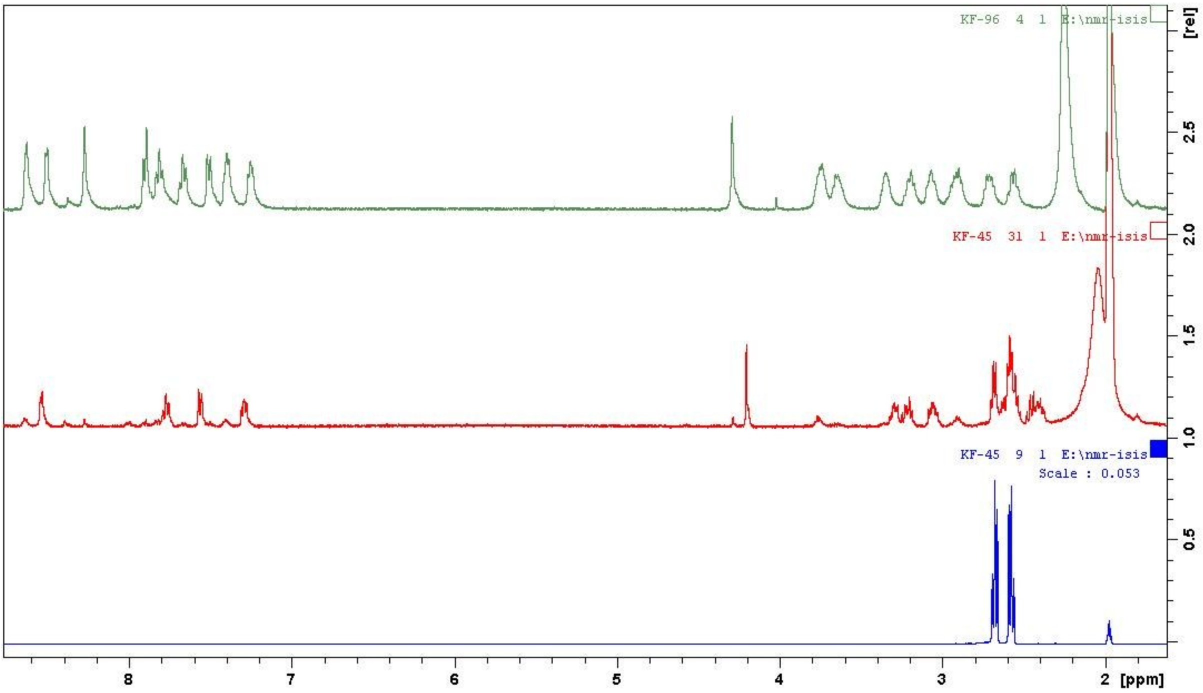

*Figure 23 ^{1}H-NMR spectra of **en_2N_3** (blue) with 1 eq. **PYRAL** (red) and 2 eq. **PYRAL** (green) in acetonitrile-d_3. With 1 eq. of the aldehyde the desired aminal is the major product and after addition of the second equivalent the corresponding imine-aminal product is formed.*

Red spectrum: aromatic part (8.5, 7.8, 7.6 and 7.3 ppm) integrally corresponds (1:1) with aliphatic part of the spectrum. Important peak is aminal at 4.2 ppm.

Green spectrum: integrals in aromatic part correspond to aliphatic part of the spectrum. Two different pyridyl unities can be observed in aromatic part (8.6, 8.5, 7.9, 7.8, 7.7, 7.5, 7.4 and 7.3 ppm) and also imine peak at 8.3 ppm. Aminal peak can be found at 4.3 ppm. Every aliphatic hydrogen has its own signal.

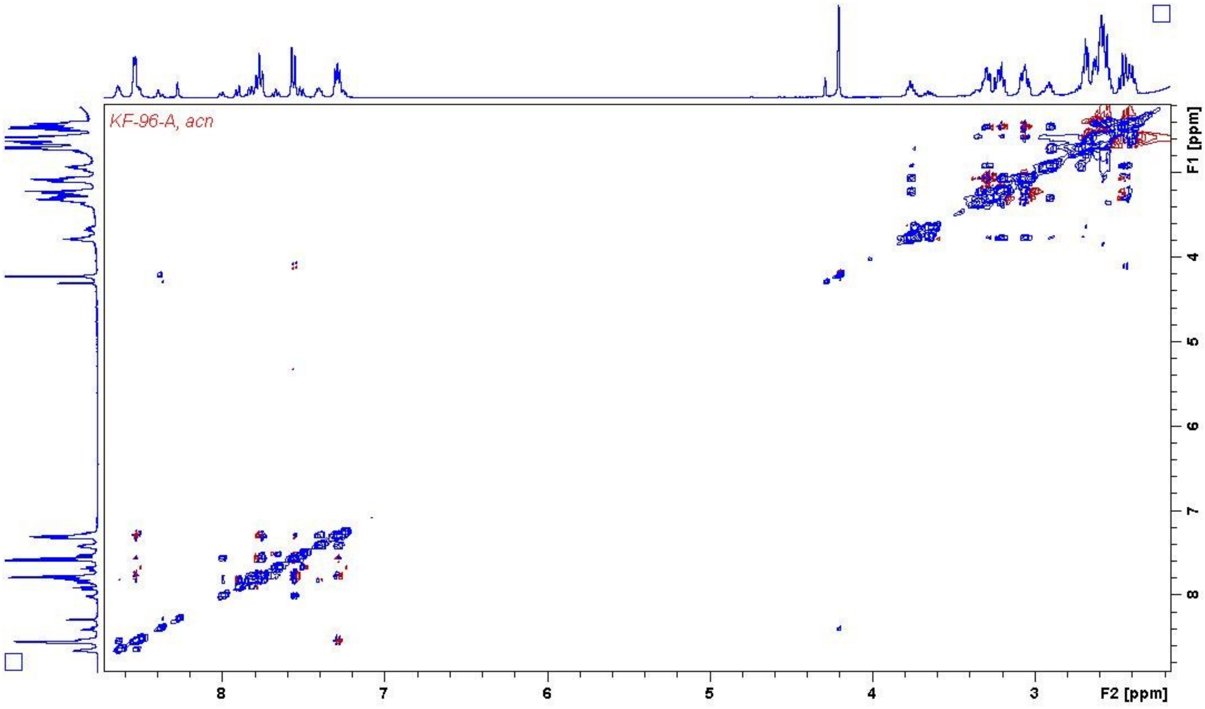

*Figure 24 ^{1}H-NOESY spectrum of **en_2N_3** with 1 equivalent of **PYRAL**. Chemical exchange crosspeaks show interconversion between the aminal (4.21 ppm, 90 %) and the imine form (8.39 ppm, 10 %).*

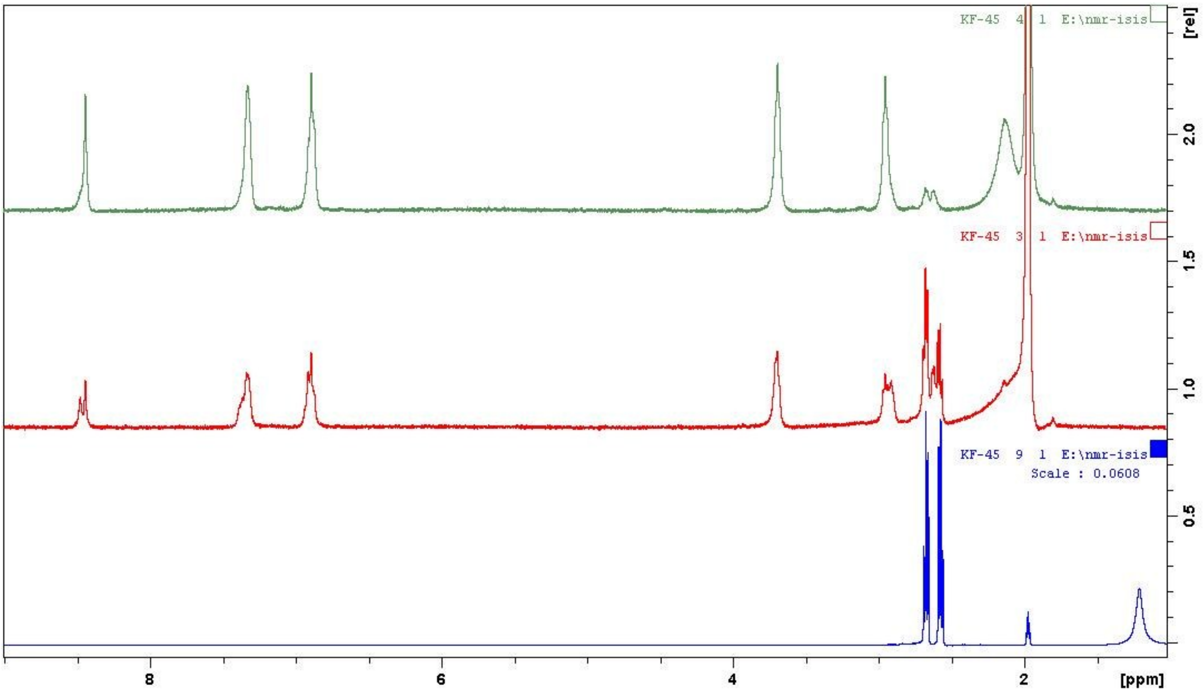

*Figure 25 ^{1}H-NMR spectra of **en_2N_3** (blue) with 1 eq. **SALAL** (red) and 2 eq. **SALAL** (green) in acetonitrile-d_3. Addition of one equivalent of the aldehyde leads to a mixture of mono-imine, bis-imine (two different imine peaks at 8.5 and 8.4 ppm) and unreacted triamine. Addition of the second equivalent clearly provides the bis-imine (one imine peak at 8.4 ppm), but the broad signals indicate rapid imine-aminal interconversion.*

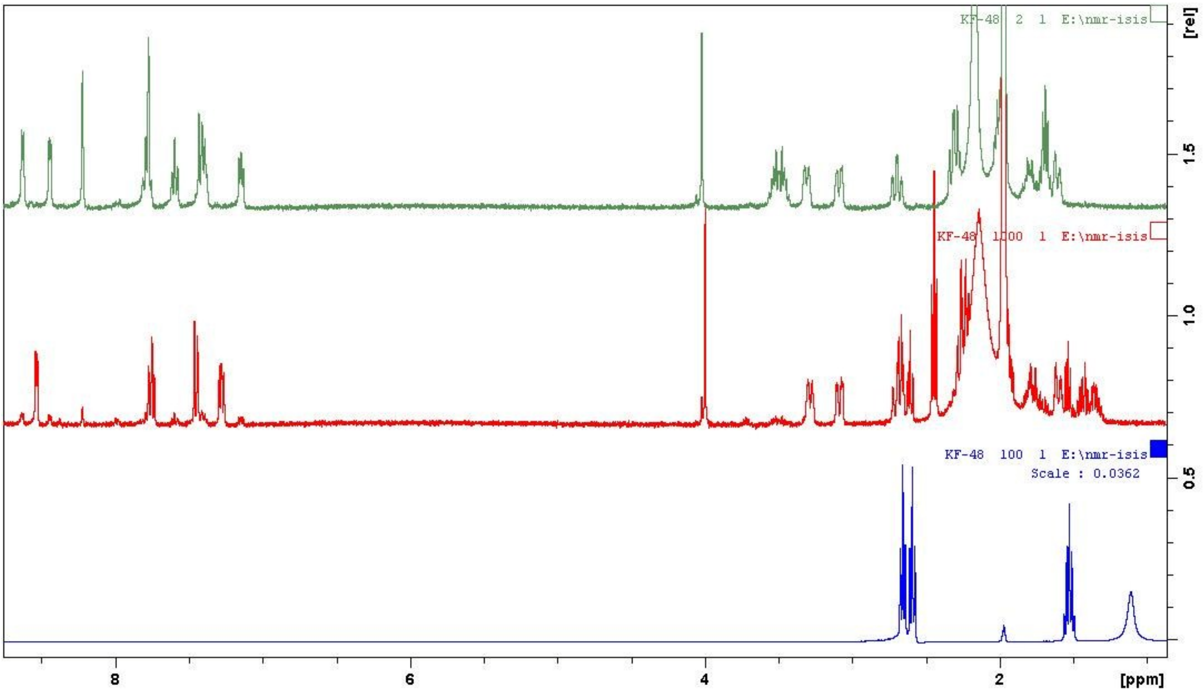

*Figure 26 ^{1}H-NMR spectra of **pr_2N_3** (blue) with 1 eq. **PYRAL** (red) and 2 eq. **PYRAL** (green) in acetonitrile-d_3. The results are analogous to the reaction of **en_2N_3** presented in Figure 23*

Red spectrum: integral intensity of pyridyl moiety (8.5, 7.8, 7.5, 7.3 ppm) corresponds to aliphatic part of the spectrum (1:1), aminal hydrogen can be clearly observed at 4.0 ppm.

Green spectrum: integrals in aromatic part correspond to aliphatic part of the spectrum. Two different pyridyl unities can be observed in aromatic part (8.6, 8.4, 7.8, 7.6, 7.4 and 7.2 ppm) and also imine peak at 8.2 ppm. Aminal peak can be found at 4.0 ppm.

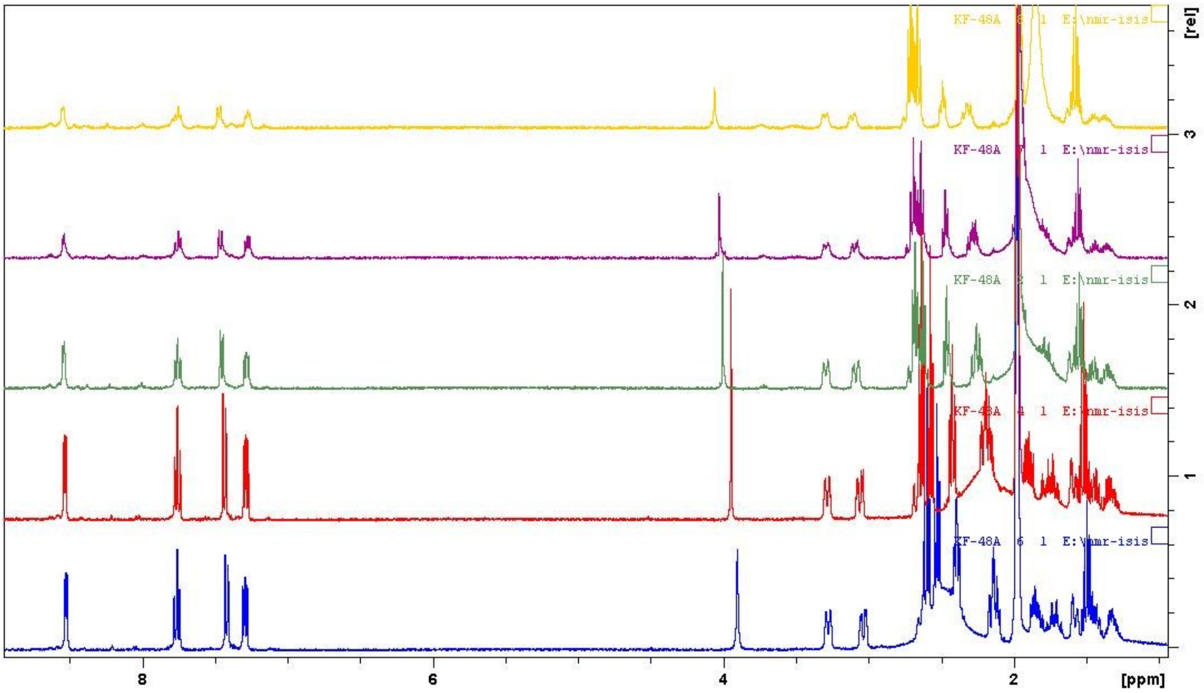

*Figure 27, ^{1}H-NMR spectra of **pr_2N_3** with 1 eq. of **PYRAL** in acetonitrile-d_3 at different temperatures (-42 °C, -10 °C, 0 °C, 40 °C, 60 °C, from bottom to top). Shift of the aminal signal from 3.9 to 4.1 ppm indicates increasing rate of exchange with the imine species (chemical shift around 8.4 ppm, Figure 28).*

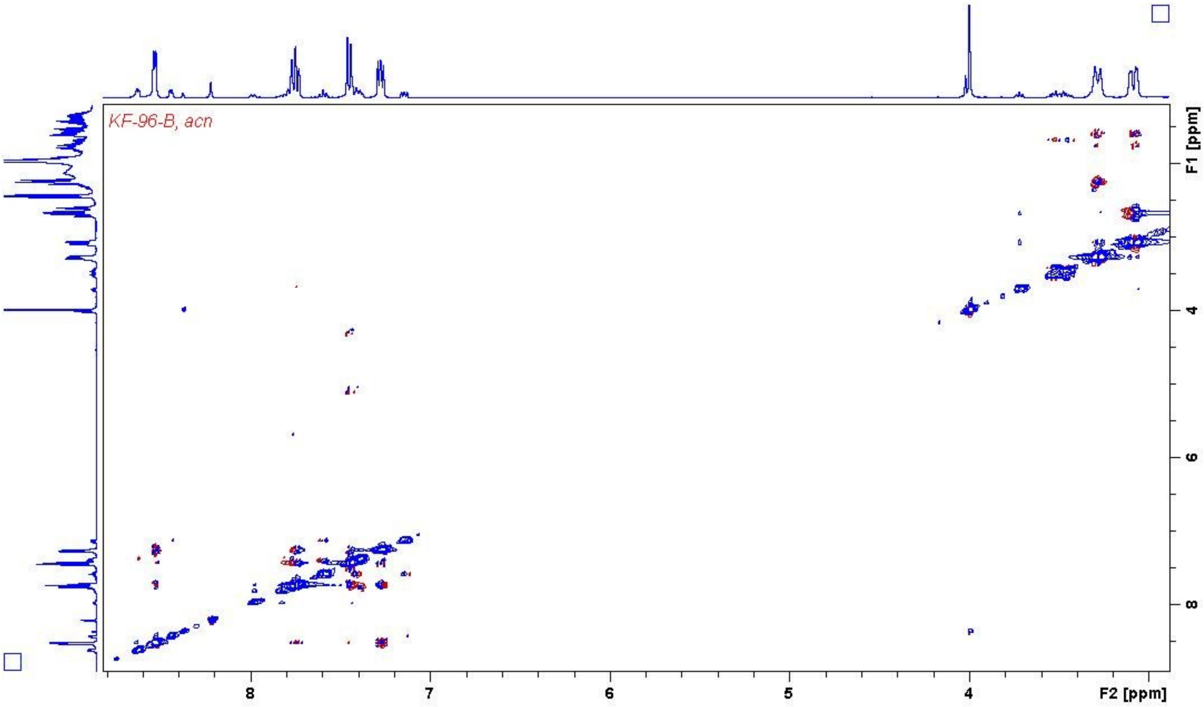

*Figure 28, ^{1}H-NOESY spectrum of **pr_2N_3** reacted with 1 eq. **PYRAL** in acetonitrile-d_3. Crosspeaks show chemical exchange between the aminal (4.00 ppm, 96 %) and the imine form (8.38 ppm, 4 %).*

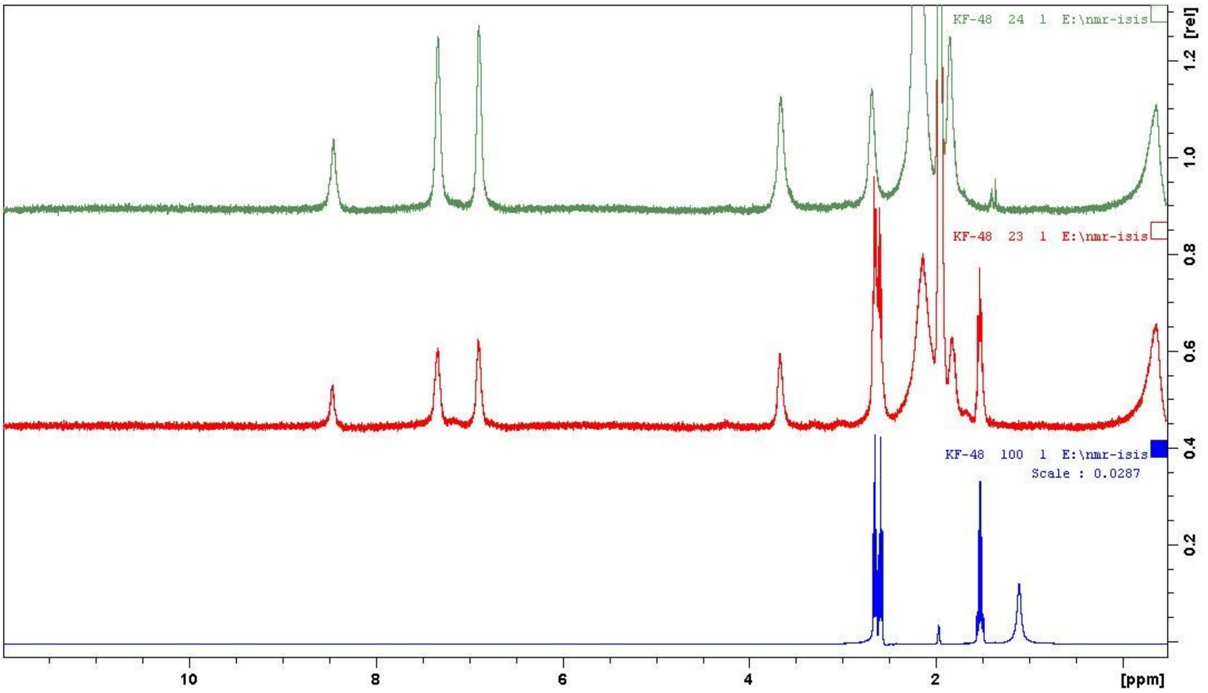

Figure 29, ^{1}H-NMR spectra of ***pr_2N_3*** *(blue) with 1 eq.* ***SALAL*** *(red) and 2 eq.* ***SALAL*** *(green) in acetonitrile-d_3. The results are analogous to the reaction with* ***en_2N_3*** *presented in Figure 25.*

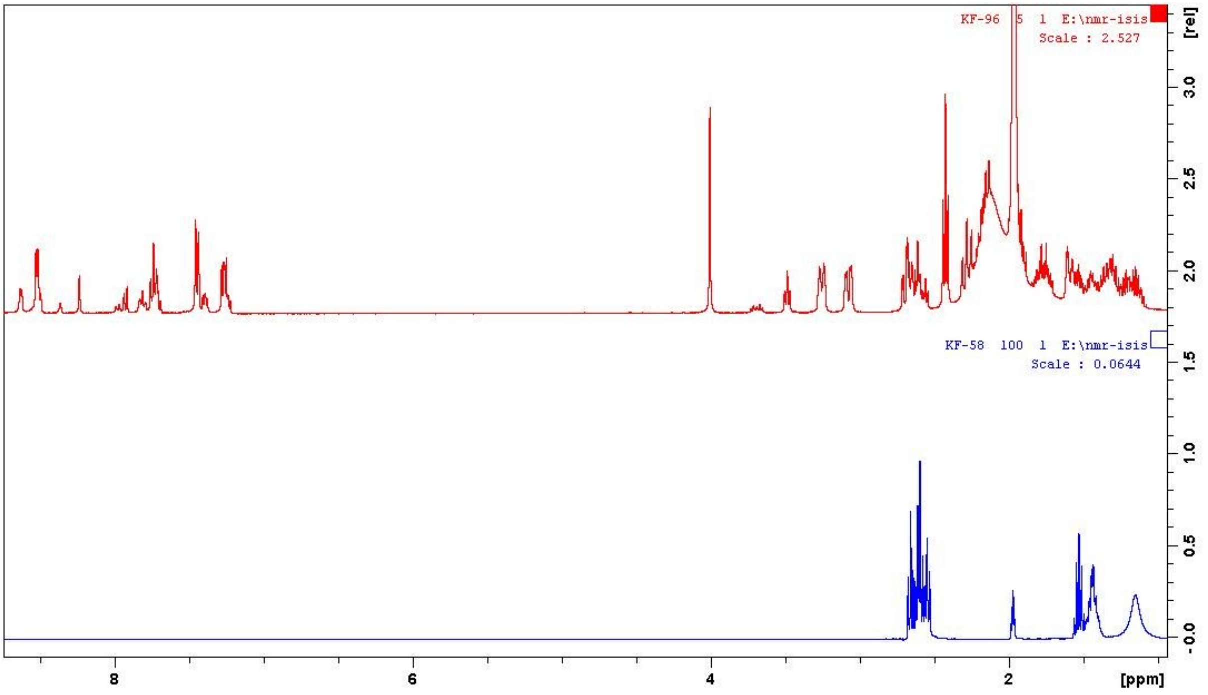

Figure 30, ^{1}H-NMR spectra of spermidine (blue) reacted with 1 eq. ***PYRAL*** *(red) in acetonitrile-d_3. The major product is the expected six-membered aminal (76 %, 4.01 ppm) accompanied by the undesired imine formed on C4 end (8.24 ppm, 17 %) and small amounts of the imine form arriving from the aminal-imine exchange appears at 8.37 ppm (7 %).*

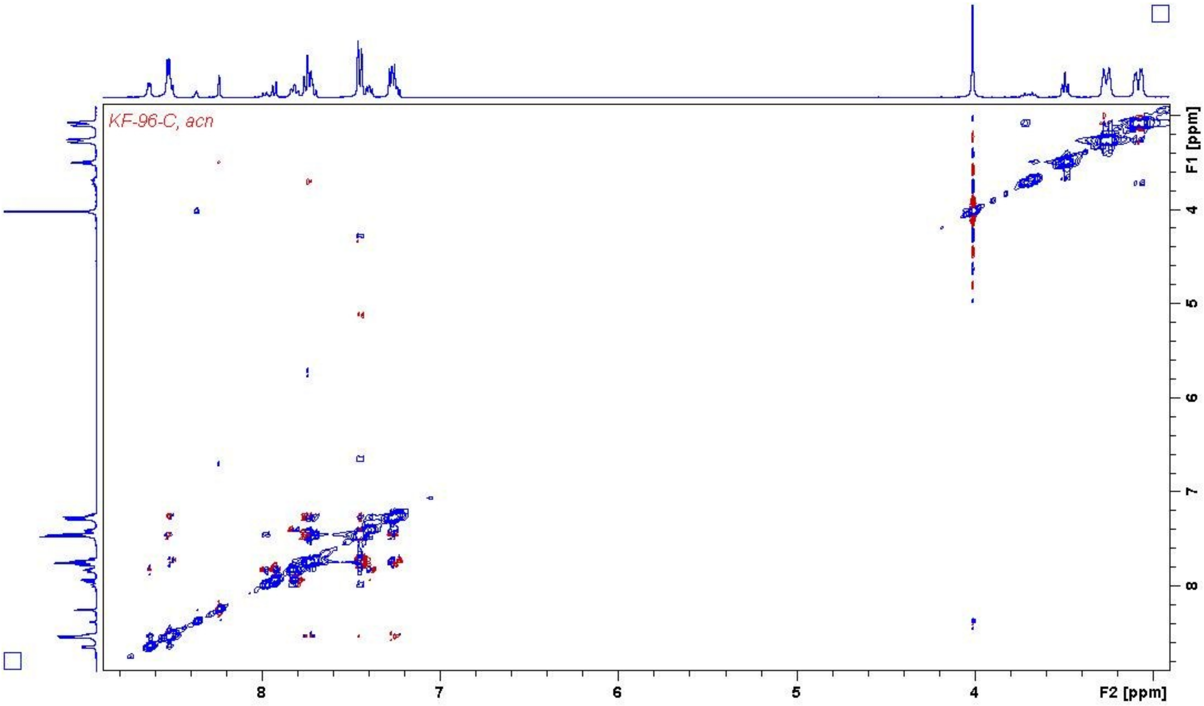

Figure 31, ^{1}H-NOESY spectrum of spermidine reacted with 1 eq. ***PYRAL*** *in CD_3CN. Chemical exchange crosspeaks between the aminal (4.01 ppm, 92 %) and the imine form (8.37 ppm, 8 %) proves the intramolecular dynamic equilibrium.*

4.5.2 Syntheses

4.5.2.1 tert-butyl bis(2-aminoethyl)carbamate hydrochloride

Bis(3-aminopropyl)amine (0.2 ml, 1.85 mmol) was stirred with SALAL (0.39 ml, 3.703 mmol) in 12 ml of acetonitrile for 2 hours. Boc anhydride (0.40 g, 1.85 mmol) was added and the reaction mixture was stirred overnight at room temperature. To deprotect the product, benzyloxyamine hydrochloride (0.59 g, 3.70 mmol) was added and stirred for 2 hours. The solvent was evaporated and to the residue CH_2Cl_2 (20 ml) and water (20 ml) were added, water phase was separated, organic phase was extracted with water and combined aqueous phases were once washed with CH_2Cl_2. Water was evaporated and the product was isolated in sufficient purity as a hydrochloride (0.37 g, 84 %).

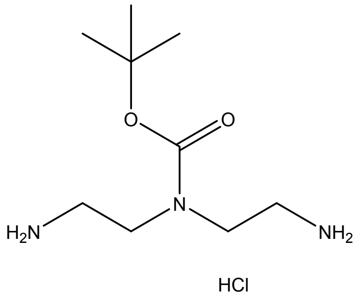

m.p. 196-197 °C

^{1}H-NMR (400 MHz, MeOH-d$_4$, 25 °C): 3.61 (t, J=6.3 Hz, 4 H, $NH_2C\mathbf{H_2}$), 3.16 (t, J= 6.3 Hz, 4 H, $C\mathbf{H_2}$), 1.54 (s, 9 H, $C\mathbf{H_3}$) ppm.

^{13}C-NMR (100 MHz, MeOH-d$_4$, 25 °C): 156.05, 81.64, 45.13, 38.04, 27.24 ppm.

MS (ESI, positive mode, 4500 V): 204 (M^+ + H)

HRMS (ESI, positive mode, 4500 V): calc. (M^+ + H): 204.1707 meas. (M^+ + H): 204.1710

4.5.2.2 tert-butyl bis(3-aminopropyl)carbamate hydrochloride

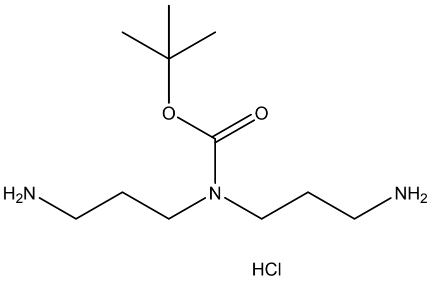

Bis(3-aminopropyl)amine (0.2 ml, 1.43 mmol) was stirred with SALAL (0.30 ml, 2.86 mmol) in 12 ml of acetonitrile for 2 hours. Boc-anhydride (0.31 g, 1.43 mmol) was added and the reaction mixture was stirred overnight at room temperature. To deprotect the product, benzyloxyamine hydrochloride (0.46 g, 3.70 mmol) was added and stirred for 2 hours. The solvent was evaporated and to the residue CH_2Cl_2 (20 ml) and water (20 ml) were added, water phase was separated, organic phase was extracted with water and combined aqueous phases were once washed with CH_2Cl_2. Water was evaporated and the white product was isolated in sufficient purity as a hydrochloride (0.34 g, 89 %).

m.p. 215.5-217 °C

^{1}H-NMR (400 MHz, MeOH-d_4, 25 °C): 3.38 (br, 4 H, $NH_2C\mathbf{H}_2$), 2.97 (t, J= 6.68 Hz, 4 H, $NC\mathbf{H}_2$), 1.96 (m, J= 7.12 Hz, 4 H, $C\mathbf{H}_2$), 1.52 (s, 9 H, $C\mathbf{H}_3$)

^{13}C-NMR (100 MHz, MeOH-d_4, 25 °C): 156.05, 81.64, 45.13, 38.04, 27.24

MS (ESI, positive mode, 4500 V): 232 (M^+ + H)

HRMS (ESI, positive mode, 4500 V): calc. (M^+ + H): 232.2020 meas. (M^+ + H): 232.2046

4.5.2.3 benzyl (2-((2-((2-aminoethyl)amino)ethyl)amino)-2-oxoethyl)carbamate hydrochloride

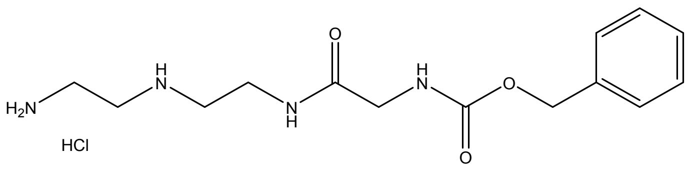

Diethylenetriamine (0.1 ml, 0.93 mmol) was dissolved in 20 ml of acetonitrile and PYRAL (0.088 ml, 0.93 mmol) was added and the mixture was equilibrated for 2 hours. CbzGlyONp (0.306 g, 0.93 mmol) was added and the reaction mixture was stirred overnight. To deprotect the polyamine, hydroxylamine was added (50 % solution in water, 0.057 ml, 0.93 mmol) and stirring continued for 2 hours. The solvent was evaporated and the mixture was separated using reversed phase chromatography (gradient H2O:ACN 99:1→5:95 with 1 ml of 0.15 M HCl added with load). The product was isolated as white solid (0.21 g, 69 %).

m.p. decomposes at 227 °C

^{1}H-NMR (400 MHz, D_2O, 25 °C): 7.37 (m, 5 H, Ar**H**), 5.07 (s, 2 H, $C\mathbf{H}_2Ph$), 3.79 (s, 2 H, $COC\mathbf{H}_2NH$), 3.53 (t, 2 H, J=5.8 Hz, $C\mathbf{H}_2$), 3.32-3.34 (m, 4 H, $C\mathbf{H}_2$), 3.23 (t, 2 H, J=5.8 Hz, $C\mathbf{H}_2$) ppm.

^{13}C-NMR (100 MHz, D_2O, 25 °C): 173.49, 158.64, 136.19, 128.84, 128.58, 127.88, 67.45, 47.98, 44.41, 43.71, 35.86, 35.53 ppm.

MS (ESI, positive mode, 4500 V): 295 (M^+ + H)

HRMS (ESI, positive mode, 4500 V): calc. (M^+ + H): 295.1765 meas. (M^+ + H): 295.1765

4.5.2.4 benzyl (3-((3-((3-aminopropyl)amino)propyl)amino)-3-oxoethyl)carbamate

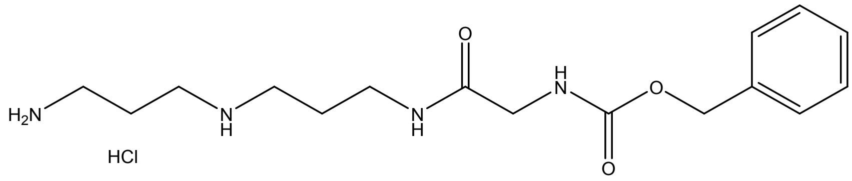

Bis(3-aminopropyl)amine (0.1 ml, 0.71 mmol) was dissolved in 20 ml of acetonitrile and PYRAL (0.068 ml, 0.71 mmol) was added and the mixture was equilibrated for 2 hours. CbzGlyONp (0.236 g, 0.71 mmol) was added and the reaction mixture was stirred overnight. To deprotect the polyamine, hydroxylamine was added (50 % solution in water, 0.044 ml, 0.71 mmol) and stirring continued for 2 hours. The solvent was evaporated and the mixture was separated using reversed phase chromatography (gradient H2O:ACN 99:1→5:95 with 1 ml of 0.15 M HCl added with the load). The product was isolated as white solid (0.16 g, 65 %).

m.p. decomposes at 224 °C

^{1}H-NMR (400 MHz, MeOH-d_4, 25 °C): 7.30-7.43 (m, 5 H, Ar**H**), 5.15 (s, 2 H, PhC**H**$_2$), 3.81 (s, 2 H, COC**H**$_2$NH), 3.39 (t, 2 H, J=6.8 Hz, NC**H**$_2$), 3.03-3.15 (m, 6 H, NC**H**$_2$), 2.12 (m, 2 H, J=7.5 Hz, C**H**$_2$), 1.94 (m, 2 H, J=6.6 Hz, C**H**$_2$) ppm.

^{13}C-NMR (100 MHz, MeOH-d_4, 25 °C): 172.38, 157.81, 136.67, 128.12, 127.69, 127.47, 66.52, 44.88, 44.51, 43.65, 36.47, 35.20, 26.17, 23.95 ppm.

MS (ESI, positive mode, 4500 V): 323 (M^+ + H)

HRMS (ESI, positive mode, 4500 V): calc. (M^+ + H): 323.2078 meas. (M^+ + Na): 323.2093

4.5.2.5 benzyl(2-(2-(((benzyloxy)carbonyl)amino)acetamido)ethyl)(2-(4-nitrobenzamido)ethyl)carbamate (D1)

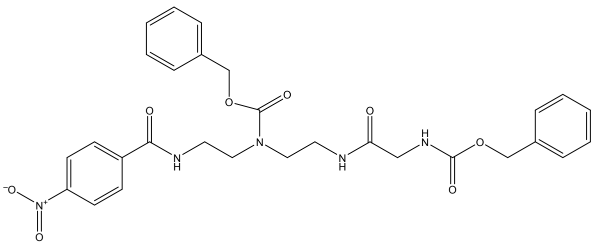

Diethylenetriamine (0.1 ml, 0.93 mmol) was dissolved in 20 ml of acetonitrile and PYRAL (0.081 ml, 0.93 mmol) was added to protect one of the primary and the secondary aminogroups. After 2 hours of stirring, Cbz-Gly-ONp (0.306 g, 0.93 mmol) was added, the solution turned yellow and the mixture was stirred overnight. To deprotect the intermediate, benzyloxyamine (0.108 ml, 0.93 mmol) was added and the reaction mixture was stirred for 3 hours. To protect the other primary aminogroup, SALAL (0.099 ml, 0.93 mmol) was added and after 2 hours the second electrophile (CbzCl, 0.132 ml, 0.926 mmol) was added. Et_3N (0.129 ml, 0.93 mmol) was also supplemented to uptake the emerging hydrochloric acid. After 16 hours, the primary aminogroup was deprotected by benzyloxyamine (0.108 ml, 0.93 mmol) and after additional 2 hours of stirring the last electrophile together with base was added (4-nitrobenzoyl chloride: 0.172 g, 0.93 mmol, Et_3N: 0.129 ml, 0.93 mmol). The suspension was stirred overnight, the the solvent was evaporated and the residue was treated with CH_2Cl_2. The precipitate was filtered off and the filtrate was evaporated and separated using column chromatography (Al_2O_3,

first CH_2Cl_2, then CH_2Cl_2:MeOH 100:1). The product was obtained as slightly yellow glass (0.18 g, 34 %).

R_f (Al_2O_3-neutral, CH_2Cl_2:MeOH 100:1) = 0.47

^{1}H-NMR (400 MHz, ACN-d_3, 70 °C): 8.24 (d, 2 H, J=8.3 Hz, Ar**H**), 7.95 (d, 2 H, J=8.3 Hz, Ar**H**), 7.51 (br, 1 H, N**H**), 7.27-7.45 (m, 10 H, Ar**H**), 6.85 (br, 1 H, N**H**), 5.86 (br, 1 H, Ar**H**), 5.11 (br, 4 H, PhC**H**$_2$), 3.71 (br, 2 H, COC**H**$_2$NH), 3.52-3.66 (m, 4 H, C**H**$_2$), 3.36-3.50 (m, 4 H, C**H**$_2$) ppm.

^{13}C-NMR (100 MHz, ACN-d_3, 70 °C): 169.66, 165.56, 156.87, 156.59, 149.74, 140.49, 137.22, 137.16, 128.40, 128.38, 128.32, 127.86, 127.77, 127.67, 127.57, 127.53, 123.47, 66.86, 66.41, 47.56, 44.31, 38.96, 38.21 ppm.

MS (ESI, positive mode, 4500 V): 578 (M^+ + H)

HRMS (ESI, positive mode, 4500 V): calc. (M^+ + Na): 600.2065 meas. (M^+ + Na): 600.2072

4.5.2.6 benzyl(3-(2-(((benzyloxy)carbonyl)amino)acetamido)propyl)(3-(4-nitrobenzamido)propyl)carbamate (D2)

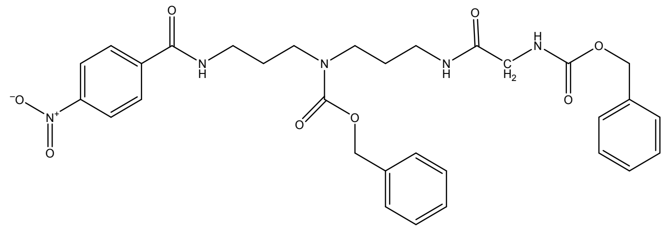

Bis(3-aminopropyl)amine (0.1 ml, 0.71 mmol) was dissolved in 20 ml of acetonitrile and PYRAL (0.068 ml, 0.71 mmol) was added to protect one of the primary and the secondary aminogroups. After 2 hours of stirring, Cbz-Gly-ONp (0.2361 g, 0.71 mmol) was added, the solution turned yellow and the mixture was stirred overnight. To deprotect the intermediate, benzyloxyamine (0.083 ml, 0.71 mmol) was added and the reaction mixture was stirred for 3 hours. To protect the other primary aminogroup, SALAL (0.076 ml, 0.71 mmol) was added and after 2 hours the second electrophile (CbzCl, 0.102 ml, 0.71 mmol) was added. Et_3N (0.100 ml, 0.71 mmol) was also supplemented to uptake the emerging hydrochloric acid. After 16 hours, the primary aminogroup was deprotected by benzyloxyamine (0.083 ml, 0.71 mmol) and after additional 2 hours of stirring the last electrophile together with base was added (4-nitrobenzoyl chloride: 0.133 g, 0.71 mmol, Et_3N: 0.100 ml, 0.71 mmol). The suspension was stirred overnight, the the solvent was evaporated and the residue was treated with CH_2Cl_2. The precipitate was filtered off and the filtrate was evaporated and separated using column chromatography (Al_2O_3, first CH_2Cl_2, then CH_2Cl_2:MeOH 100:1). The product was obtained as slightly yellow glass (0.1 g, 23 %).

R_f (Al_2O_3-neutral, CH_2Cl_2:MeOH 100:1) = 0.45

^{1}H-NMR (400 MHz, DMSO-d_6, 70 °C): 8.58 (br, 1 H, N**H**), 8.28 (d, J= 9.1 Hz, 2 H, Ar), 8.05 (d, J= 9.1 ppm, 2 H, Ar), 7.65 (br, 1 H, N**H**), 7.26-7.38 (m, 10 H, Ph), 7.13 (br, 1 H, N**H**), 5.09 (s, 2 H, C**H**$_2$Ph), 5.05 (s, 2 H, C**H**$_2$Ph), 3.60 (d, J= 5.9 Hz, 2 H, C**H**$_2$NH), 3.23-3.35 (m, 6 H, C**H**$_2$N), 3.06-3.12 (m, 2 H, C**H**$_2$N), 1.82 (m, J=6.6 Hz, 2 H, C**H**$_2$), 1.68 (m, J= 7.4 Hz, 2 H, C**H**$_2$) ppm.

^{13}C-NMR (100 MHz, DMSO-d_6, 70 °C, 25 °C): 169.32, 165.12, 155.90, 149.65, 140.96, 137.66, 137.13, 129.05, 128.80, 128.73, 128.14, 128.09, 128.01, 127.76, 123.83, 66.67, 66.03, 45.38, 45.26, 44.40, 37.85, 36.86, 28.85, 28.55 ppm.

MS (ESI, positive mode, 4500 V): 606 (M^+ + H)

HRMS (ESI, positive mode, 4500 V): calc. (M^+ + Na): 628.2378 meas. (M^+ + Na): 628.2381

4.5.2.7 benzyl(4-(2-(((benzyloxy)carbonyl)amino)acetamido)butyl)(3-(4-nitrobenzamido)propyl)carbamate (D3)

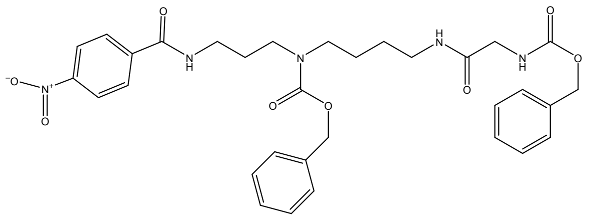

Spermidine (0.1 ml, 0.637 mmol) was dissolved in 20 ml of acetonitrile and PYRAL (0.0606 ml, 0.64 mmol) was added to protect one of the primary and the secondary aminogroups. After 2 hours of stirring, Cbz-Gly-ONp (0.2103 g, 0.64 mmol) was added, the solution turned yellow and the mixture was stirred overnight. To deprotect the intermediate, benzyloxyamine (0.074 ml, 0.64 mmol) was added and the reaction mixture was stirred for 3 hours. To protect the other primary aminogroup, SALAL (0.068 ml, 0.64 mmol) was added and after 2 hours the second electrophile (CbzCl, 0.091 ml, 0.64 mmol) was added. Et_3N (0.089 ml, 0.64 mmol) was also supplemented to uptake the emerging hydrochloric acid. After 16 hours, the primary aminogroup was deprotected by benzyloxyamine (0.074 ml, 0.64 mmol) and after additional 2 hours of stirring the last electrophile together with base was added (4-nitrobenzoyl chloride: 0.118 g, 0.64 mmol, Et_3N: 0.089 ml, 0.64 mmol). The suspension was stirred overnight, the the solvent was evaporated and the residue was treated with CH_2Cl_2. The precipitate was filtered off and the filtrate was evaporated and separated using column chromatography (Al_2O_3, first CH_2Cl_2, then CH_2Cl_2:MeOH 100:1). The product was obtained as slightly yellow glass (0.12 g, 31 %).

R_f (Al_2O_3-neutral, CH_2Cl_2:MeOH 100:1) = 0.40

^1H-NMR (500 MHz, DMSO-d_6, 25 °C): 8.79 (br, 1 H, N**H**), 8.30 (br, 2 H, Ar**H**), 8.04 (br, 2 H, Ar**H**), 7.84 (br, 1 H, N**H**), 7.42 (t, 1 H, J=5.8 Hz, N**H**), 7.23-7.39 (m, 10 H, Ar**H**), 4.97-5.09 (m, 4 H, PhC**H$_2$**), 3.56 (d, 2 H, J=5.7 Hz, COC**H$_2$**NH), 3.14-3.31 (m, 6H, C**H$_2$**N), 3.04 (br, 2 H, C**H$_2$**N), 1.77 (br, 2 H, C**H$_2$**), 1.47 (br, 2 H, C**H$_2$**), 1.35 (br, 2 H, C**H$_2$**) ppm.

^{13}C-NMR (125 MHz, DMSO-d_6,, 25 °C): 169.31, 165.04, 156.92, 155.81, 149.40, 140.72, 137.52, 137.51, 129.10, 128.88, 128.82, 128.26, 128.19, 127.86, 124.00, 66.47, 65.91, 46.67*, 44.95*, 44.01, 38.63*, 37.62, 28.50*, 28.85, 25.78* ppm.

*broad

MS (ESI, positive mode, 4500 V): 620 (M^+ + H)

HRMS (ESI, positive mode, 4500 V): calc. (M^+ + Na): 642.2534 meas. (M^+ + Na): 642.2556

5. References

(1) Gottlieb, H. E.; Kotlyar, V.; Nudelman, A. *J. Org. Chem.* **1997**, *62* (21), 7512–7515.
(2) Lumry, R.; Smith, E. L.; Glantz, R. R. *J. Am. Chem. Soc.* **1951**, *73* (9), 4330–4340.
(3) Sarma, R. J.; Otto, S.; Nitschke, J. R. *Chem. - Eur. J.* **2007**, *13* (34), 9542–9546.
(4) Kovaříček, P.; Lehn, J.-M. *J. Am. Chem. Soc.* **2012**, *134* (22), 9446–9455.
(5) Bednarz, S.; Bogdal, D. *Int. J. Chem. Kinet.* **2009**, *41* (9), 589–598.
(6) Kovaříček, P.; Lehn, J.-M. *Chem. – Eur. J.* **2015**, *21* (26), 9380-9384
(7) Cordes, E. H.; Jencks, W. P. *J. Am. Chem. Soc.* **1962**, *84*, 832–837.
(8) Hopgood, D.; Leussing, D. L. *J. Am. Chem. Soc.* **1969**, *91* (14), 3740–3750.
